# Add-on effects of Chinese herbal medicine external application (FZHFZY) to topical urea for mild-to-moderate psoriasis vulgaris: Protocol for a double-blinded randomized controlled pilot trial embedded with a qualitative study

**DOI:** 10.1371/journal.pone.0297834

**Published:** 2024-03-21

**Authors:** Junyue Wang, Claire Shuiqing Zhang, Anthony Lin Zhang, Jingjie Yu, Hao Deng, Haiming Chen, Charlie Changli Xue, Chuanjian Lu

**Affiliations:** 1 The China-Australia International Research Centre for Chinese Medicine, School of Health and Biomedical Sciences, RMIT University, Melbourne, Victoria, Australia; 2 The Second Affiliated Hospital of Guangzhou University of Chinese Medicine, Guangdong Provincial Hospital of Chinese Medicine and Guangdong Provincial Academy of Chinese Medical Sciences, Guangzhou, China; 3 Guangdong-Hong Kong-Macau Joint Lab on Chinese Medicine and Immune Disease Research, Guangzhou University of Chinese Medicine, Guangzhou, China; 4 State Key Laboratory of Dampness Syndrome of Chinese Medicine, The Second Affiliated Hospital of Guangzhou University of Chinese Medicine, Guangzhou, China; Xiamen University - Malaysia Campus: Xiamen University - Malaysia, MALAYSIA

## Abstract

Psoriasis vulgaris is a chronic dermatological disease with a high global prevalence. It significantly reduces patients’ quality of life and is associated with a substantial economic burden. Conventional therapies for mild-to-moderate psoriasis are often associated with insufficient long-term symptomatic relief and side effects. Chinese herbal medicine (CHM) is commonly used for psoriasis management. A CHM formula, namely *Fu zheng he fu zhi yang* (FZHFZY), has shown promising treatment effects in clinical practice when used as a bath therapy. However, its efficacy and safety has not been evaluated by a rigorous randomized controlled trial (RCT). Therefore, we designed a double-blinded pilot RCT embedded with a qualitative study on CHM formula FZHFZY plus topical urea for mild-to-moderate psoriasis vulgaris to advance the evidence development and practice of CHM external application for psoriasis. This will be a mixed-method design consisting of a pilot RCT and a qualitative study. The pilot RCT is a two-arm, parallel, placebo-controlled, double-blinded trial. Sixty eligible participants will be randomized at a 1:1 ratio to receive eight weeks’ treatment of either FZHFZY plus 10% urea cream, or placebo plus 10% urea cream, with 12-week follow-up visits after the treatment phase. The CHM or placebo will be administered externally as a bath therapy. Outcome measures include trial feasibility, efficacy and safety. The primary efficacy outcome will be Psoriasis Area Severity Index (PASI). Secondary efficacy outcomes include Physician Global Assessment, PASI-75, PASI-50, Body Surface Area, Dermatology Life Quality Index, Skindex-16, itch visual analogue scale and relapse. The qualitative study will be conducted to collect participants’ feedback on CHM external application and their experience with the pilot RCT. This study will advance the evidence-based clinical practice of using CHM for psoriasis vulgaris and then to support translation of findings into clinical practice in the future.

**Trial registration number:** ChiCTR2200064092.

## Introduction

Psoriasis is a chronic, recurrent inflammatory condition caused by immune stimulation of epidermal keratinocytes [1]. The cumulative global prevalence of psoriasis was reported to be between 0.09% and 11.4% in 2016 by WHO [2]. In 2017, the Global Burden of Disease Study ranked psoriasis as the tenth most prevalent skin disease (0.88%) and the second largest contributor to disability-adjusted life years (0.22%) among all skin diseases [3]. Life-long psoriasis symptoms and associated comorbidities can significantly impair patients’ quality of life (QoL) and cause substantial economic burden [2, 4]. Psoriasis vulgaris, characterized by raised, well-demarcated, erythematous oval plaques with adherent silvery scales, is the most common form of the disease accounting for more than 80% of total psoriasis cases [5].

Most psoriasis vulgaris patients are of mild-to-moderate severity [6], which is usually treated with topical agents and phototherapies in clinical practice. However, these therapies are often associated with concerning adverse effects. For example, although topical corticosteroids and vitamin D analogues are recommended as effective treatments with Grade A strength [7], corticosteroids may cause skin atrophy, striae, folliculitis, telangiectasia, purpura, tachyphylaxis and rebound [8]; while topical vitamin D analogue causes burning, pruritus, oedema, peeling, dryness and erythema in 35% of patients [7]. Narrowband ultraviolet light B (NB-UVB) phototherapy and targeted UVB phototherapy are also recommended for adults with mild-to-moderate psoriasis [9]. The NB-UVB is associated with a risk of skin cancer and cataracts [9], and targeted UVB phototherapy usually causes painful erythema and blistering [10]. Therefore, patients often seek complementary therapies to manage psoriasis vulgaris.

Chinese herbal medicine (CHM) applies a unique holistic theory to guide individualized treatments, and has been practiced for thousands of years [11]. Research evidence generated by systematic reviews of randomized controlled trials (RCTs) showed that externally applied CHM, used as an add-on therapy to conventional therapies, can improve psoriasis vulgaris symptoms [12–18]. Evidence from *in vitro* and *in vivo* studies also indicated that externally applied CHM plays an important role in psoriasis treatment by increasing the amount of granulocyte colony-stimulating factor [19] and inhibiting human neutrophil activation [20]. Of note, the latest American Academy of Dermatology clinical guideline on psoriasis states that CHM, externally applied as a bath therapy, appeared to improve psoriasis patients’ response to conventional treatments, although it is difficult to interpret and replicate the results since most clinical trials lacked standardization and the CHM formula constituents were unclear [7].

The CHM formula *Fu zheng he fu zhi yang* (FZHFZY) was developed by Prof. Chuanjian Lu, an experienced clinical dermatologist at the Guangdong Provincial Hospital of Chinese Medicine (GPHCM). The FZHFZY has been used as an external application in clinical practice to manage psoriasis symptoms for around 15 years [21]. As observed in real-world practice, FZHFZY produces therapeutic effects on psoriasis vulgaris by promoting skin lesion healing and relieving pruritus. However, the efficacy and safety of this formula has not been evaluated by a rigorous RCT. Therefore, we designed this double-blinded, placebo-controlled RCT protocol, and will conduct a pilot RCT to collect preliminary data and explore the protocol’s feasibility. We will also collect participants’ feedback on CHM therapy for psoriasis vulgaris and their experience with the pilot trial through an embedded qualitative study.

The aims of this study are to explore the feasibility and acceptability of the trial protocol qualitatively and quantitatively, and to collect preliminary data on the efficacy and safety of using CHM formula FZHFZY as an additional treatment alongside a topical urea cream in patients with mild-to-moderate psoriasis vulgaris. The specific objectives are to: 1) evaluate the feasibility of the study design, including its setting, eligibility criteria, interventions, outcome measures, participant timeline and recruitment strategies; 2) explore participants’ blinding credibility and their acceptability on the trial; 3) collect preliminary data on the efficacy of using CHM formula FZHFZY as an additional treatment to inform the sample size for a future, full-scale RCT; 4) explore the safety of the proposed interventions; and 5) obtain participants’ feedback on FZHFZY and their experience with the pilot RCT.

A protocol is commonly defined as “a comprehensive plan outlining the details of a study”. Making a protocol publicly accessible enhances the transparency of research and allows researchers and potential participants to gain knowledge about ongoing trials. Protocol availability also facilitates systematic reviewers, sponsors, regulators and other stakeholders to comprehend the scientific rigor of the study design and results. It allows them to compare the intended research objectives with the actual description in trial reports, thereby assessing potential reporting bias [22]. Publishing the trial protocol holds significant value in preventing redundant research, promoting explicit research conduct and analysis, as well as informing ongoing clinical trials.

## Methods

### Study design

The pilot RCT is designed as a two-arm, parallel, randomized, placebo-controlled, double-blinded trial. Sixty eligible participants will be randomized at a 1:1 ratio to receive eight weeks of treatment with either CHM FZHFZY granules as a bath therapy plus 10% urea cream, or placebo granules as a bath therapy plus 10% urea cream. They will then undergo a 12-week follow-up phase with urea cream only. The RCT protocol will follow the recommended elements in the Standard Protocol Items: Recommendations for Interventional Trials (SPIRIT) checklist (S1 File) [23].

A qualitative component will be embedded in the pilot RCT. We will recruit up to 30 participants from those who have completed the RCT. These participants will complete semi-structured interviews within four weeks of completing the RCT.

**Fig 1:**
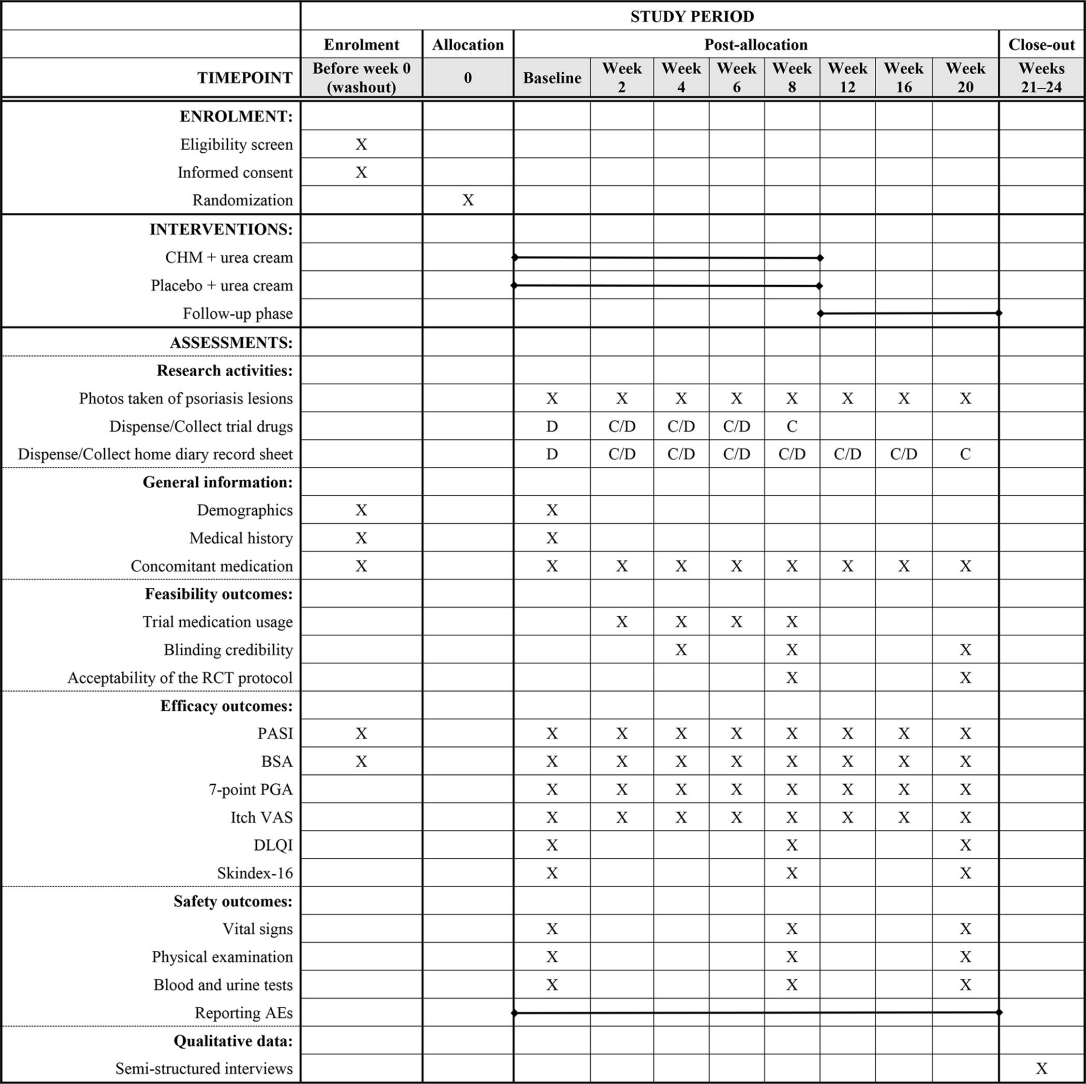
SPIRIT schedule of enrolment, interventions, and assessments. The figure shows the SPIRIT schedule of enrolment, interventions, and assessments for this mixed-method clinical study, consisting of a 20-week pilot RCT and a qualitative study after the RCT within 4 weeks. The pilot RCT includes a washout phase, an 8-week treatment phase and a 12-week follow-up phase. Data collection includes quantitative data of trial feasibility, efficacy and safety outcomes, as well as qualitative data from semi-structured interviews. AE, adverse event; BSA, Body Surface Area; C, collect; D, dispense; DLQI, Dermatology Life Quality Index; PASI, Psoriasis Area and Severity Index; PGA, Physician’s Global Assessment; VAS, visual analogue scale.

**Fig 2:**
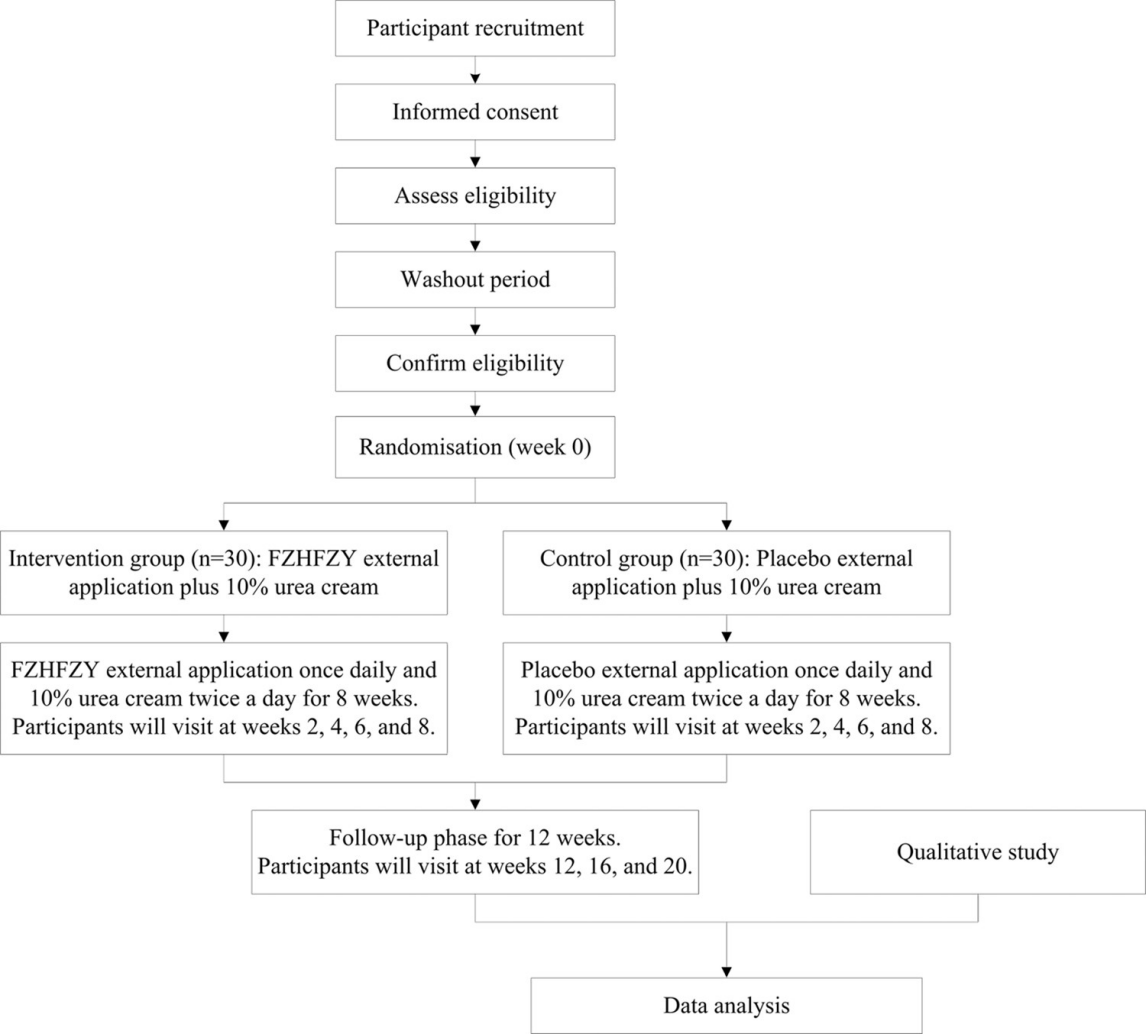
Flowchart of the trial procedure. The figure shows the flowchart of the clinical study procedure consisting of a pilot RCT and a qualitative study. Sixty eligible participants will be randomized at a 1:1 ratio to receive eight weeks of treatment with either CHM FZHFZY granules as a bath therapy plus 10% urea cream, or placebo granules as a bath therapy plus 10% urea cream; they will then undergo a 12-week follow-up phase with urea cream only. Up to 30 participants completing the pilot RCT will participate in a qualitative study.

The SPIRIT schedule of tests and procedures for this study can be found in Fig 1. The flowchart is presented in Fig 2.

### Pilot RCT

#### Setting and participants

This RCT will be conducted at GPHCM, Guangzhou, China. GPHCM is the largest Chinese medicine hospital in China which provides both Western medicine and Chinese medicine therapies. There are around 6.8 million outpatient visits at GPHCM each year. The total number of psoriasis vulgaris patients visiting GPHCM is more than 2,000 annually.

In this pilot RCT, participants with mild-to-moderate psoriasis vulgaris will be recruited through advertising posters (S2 File) and face-to-face consultations at the GPHCM dermatology outpatient department. Physicians (e.g., dermatologists or immunologists) may also recommend their patients contact researchers about participating in the trial. Potential participants will be invited to an initial assessment (first visit) through screening forms (S3 File). Eligible participants will be included in the RCT after providing written informed consent.

#### Selection criteria for the pilot RCT

Inclusion criteria.

- clinically diagnosed with psoriasis vulgaris, a clinical condition characterized by distinct, sharply defined bright red plaques on the skin that are typically covered by silvery white scales, as stated in the SIGN guidelines (SIGN, Scottish Intercollegiate Guidelines Network) [24]
- PASI < 10 or BSA < 10% [7, 25]
- aged between 18 and 65 years, with no restriction placed on gender
- provided written informed consent with adhering to bath administrations (S4 File).

Exclusion criteria.

- pregnant and/or lactating
- uncontrolled or severe systemic diseases, including cardiovascular, respiratory, hematological or psychiatric diseases, or a history of cancer

- allergy to the medications used in this study
- participating in other clinical trial(s), or have participated in other clinical trial(s) in the previous month
- having not completed a required washout period of other psoriasis therapies (see Table 1 for detailed washout requirements of each therapy).

**Table 1:** Psoriasis therapies and washout requirements.

Therapies	Washout requirements
Topical agents (including corticosteroids, calcineurin inhibitors, vitamin D analogues, tazarotene, salicylic acid, anthralin and coal tar) [7]	Two weeks
Antimicrobials, systemic nonbiologic therapies (including methotrexate, cyclosporine, acitretin and apremilast) and phototherapies (including NB-UVB, BB-UVB and PUVA) [9, 26, 27]	Four weeks
Biologics [28]	Five times of a half-life period of biologics [29, 30]: • Etanercept 3.5 × 5 days • Infliximab 10 × 5 days • Adalimumab 14 × 5 days • Ustekinumab 21 × 5 days • Guselkumab 18 × 5 days • Secukinumab 27 × 5 days • Ixekizumab 13 × 5 days

Note: BB-UVB, Broadband ultraviolet B; NB-UVB, Narrowband ultraviolet light B; PUVA, psoralen combined with ultraviolet A.

#### Randomization and allocation concealment

At the baseline visit, 60 eligible participants will be randomly assigned at a 1:1 ratio to either the CHM or placebo group. The random allocation sequence will be generated through a permuted-block randomization method with varied block sizes using SAS software (version 9.2, SAS Institute, Inc, Cary, NC, USA). An independent researcher from the Key Unit of Methodology in Clinical Research at GPHCM will be the only person with access to the group allocation of each randomization code. They will prepare a randomization schedule and label the packages of trial medication (CHM or placebo) with randomization codes. After eligibility screening and informed consent, trial investigators will enter eligible participant’s information to an online randomization program which is developed by the Key Unit of Methodology in Clinical Research to obtain a randomization code for the participant. A research assistant who is not involved in recruitment and outcome assessment will then dispense the corresponding medication to the participant.

#### Blinding

The randomization schedule will be concealed until completing the whole trial and data analyses. The participants, trial investigators, outcome assessors and research assistants will not know the group allocation until the end of the entire trial. Trial medications of CHM granule and the matched placebo granule will be produced identical in terms of their color, appearance, smell, solubility and particle size. Packaging and labelling of trial medications will be conducted by independent personnel out of trial investigators. Blinding credibility test will be conducted at week 4, 8 and 20; each participant will be asked whether they believe they were given placebo, CHM or unsure. The randomization code can be broken if necessary to allow for appropriate management of a patient in a medical emergency. In such scenarios, the principal investigator would need to approve breaking the code, the situation would be reported to the Ethics Committee, with the reason, date and result of breaking the randomization code recorded in the participants’ case report forms. If a participant’s treatment is unblinded by the investigator, the participant’s involvement in the study will end.

#### Intervention and control

*FZHFZY formula*. The CHM formula, FZHFZY, consists of seven herbs: *Cynanchum paniculatum* (*Bge*.) Kitag. (*xu chang qing*) 30 g, *Dictamnus dasycarpus* Turcz. (*bai xian pi*) 30 g, *Cnidium monnieri* (L.) Cuss. (*she chuang zi*) 20 g, *Smilax glabra* Roxb. (*tu fu ling*) 30 g, *Rehmannia glutinosa* Libosch. (*shu di huang*) 30 g, *Angelica sinensis* (Oliv.) Diels. (*dang gui*) 20 g and *Punica granatum* L. (*shi liu pi*) 30 g [21].

FZHFZY granules are produced by Tianjiang Pharmaceutical Co, Ltd (Jiangyin, Jiangsu Province, China), a manufacturer holding a Chinese Good Manufacturing Practice certificate. These seven herbs at a ratio of 3:3:2:3:3:2:3 will be mixed, boiled, filtered and pressure spray-dried to form granules. The granules will be packaged in single-dose sachets (11.0 cm × 15.5 cm), weighing 100 g each.

The trial medication, FZHFZY granules underwent analysis using high-performance liquid chromatography (HPLC) which revealed the presence of nine active compounds adenosine, ferulic acid, neoastilbin, astilbin, isoastilbin, neoisoastilbin, isomaculosidine, paeonol, and osthole. Two main active compounds, paeonol and astilbin, have been found effective in managing psoriasis. Previous studies have shown that paeonol can regulate the expression of gene autophagy-related 5 to manage psoriasis *in vitro* [31], and inhibit the maturation and activation of dendritic cells, leading to the amelioration of imiquimod-induced psoriasis-like skin lesions *in vivo* [32]. Similarly, topical application of astilbin has been found to inhibit the dendritic cell-Th17 inflammation axis and improve imiquimod-induced psoriasis-like skin lesions in SKH-1 mice [33]. Additionally, astilbin has been found to reduce reactive oxygen species accumulation and vascular endothelial growth factor expression through Nrf2 in psoriasis-like skin diseases [34]. Therefore, it is important to determine the accurate concentration of paeonol and astilbin in FZHFZY granules. The HPLC analysis revealed that the concentration of paeonol and astilbin in FZHFZY granules was not less than 1.56 mg/g and 0.27 mg/g, respectively. Quantitative analysis of active compounds and the HPLC fingerprint of FZHFZY granules have been provided in the S5 File.

*Placebo*. The placebo will be produced by the same manufacturer to match the FZHFZY granules as closely as possible in terms of their colour, appearance, smell, solubility and particle size. The placebo granules consist of maltodextrin, tartrazine, sunset yellow, caramel pigment and bitterant. It will have no active ingredients.

*Treatments*. Participants will use the FZHFZY formula or placebo at home once a day for eight weeks. The following administration instructions and precautions will apply to both the FZHFZY and placebo groups.

*Administration instructions*. Participants will dissolve one sachet of FZHFZY or placebo granules in warm water at 35–38°C [35–37] with 60 liters in a container. They will be instructed to keep their lesions under the water for 15–20 minutes each time [37–39]. An exact temperature and duration of the bath therapy is unable to be defined due to limited information provided in current clinical guidelines. Therefore, a range of bath temperature and duration based on previous clinical trials and the climate conditions of the trial site is established for this pilot RCT. Participants will be advised to check the bath temperature using a water thermometer and be strongly encouraged to set an alarm to ensure that they will follow trial instructions. The amount of bath water will be adjusted according to the size of the container and lesion location with the fixed concentration of trial medications. Where a whole-body bath is not needed, participants may reduce the amount of bath water but follow a fixed concentration.

*Administration precautions*. Participants will be advised to keep the water below chest height if they are in a semi-reclining or sitting position in a whole-body bath. They will need to avoid being cold and drinking alcohol during the trial. If they have a cold or fever, skin damage caused by injuries, or any allergic reaction to the CHM/placebo, they will be advised to stop the bath therapy and seek suggestions from the trial investigator.

*Co-intervention*. Participants in each group will also be given 10% urea cream produced by Shanghai Scond Pharmaceutical Co, Ltd (Shanghai Province, China) to apply topically on their lesions twice a day. They will be advised to follow the fingertip unit measurement method. One fingertip unit is the amount of medication (about 0.5 grams) that covers the tip of an adult index finger to the first crease, and is sufficient to cover both sides of an adult hand [40, 41]. Participants will be instructed to apply the urea cream after the bath therapy, while the skin is still moist. In addition, participants will receive cetirizine hydrochloride tablets produced by Chengdu Leer Pharmaceutical Co, Ltd (Chengdu, Sichuan Province, China) to relieve severe itchiness caused by psoriasis if it affects sleep.

#### Outcome measures

*Trial feasibility*. Feasibility studies are used to determine whether an intervention is appropriate for further testing [42]. There are four different feasibility outcomes for a pilot study, consisting of stop (full RCT not feasible), continue but modify protocol (feasible with modification), continue without modification but monitor closely (feasible with close monitoring), and continue without modification (feasible as is) [43]. In this pilot RCT, the recruitment rate and time required to recruit the target number of participants will be used to assess the feasibility of the study setting, eligible criteria and recruitment strategies. Blinding credibility will be assessed by asking participants to which group they believe they have been assigned. The frequency of missing data will be used to assess the feasibility of data collection. Participant adherence will be assessed by research investigators according to times of participants visit, records of participant’s home diary at the treatment duration (S6 File), and trial medication usage. Participants will be asked to return unused trial medications at the next clinic visit. We will then calculate the compliance based on used trial medication recorded in the participant’s home diary and returned trial medication. An 11-point ordinal scale score will be used to quantitatively evaluate the participants’ satisfaction of the trial [44].

*Efficacy outcomes*. The primary efficacy outcome will be the change of Psoriasis Area and Severity Index (PASI) from baseline to week 8 [45], this will also be used to calculate the sample size for a full-scale RCT.

Secondary efficacy outcomes are the:

- number of participants who achieve a score of 0 (clear) or 1 (almost clear) on a 7-point static Physician Global Assessment (PGA) at week 8 [45]
- number of participants who improve by at least 75% from baseline PASI (PASI-75) at week 8 [46]
- number of participants who improve by at least 50% from baseline PASI (PASI-50) at weeks 8 [46]

- change of Body Surface Area (BSA) from baseline to week 8 [47]
- change of itch visual analogue scale (VAS) from baseline to week 8 [48]
- change of Dermatology Life Quality Index (DLQI) from baseline to week 8 [49]

- change of Skindex-16 score from baseline to week 8 [50]
- relapse rate at week 20 –‘relapse’ is defined as a loss of 50% of PASI improvement from baseline in participants who achieved PASI-50 at week 8 [51].

*Safety outcomes*. Safety assessments will consist of the frequency of adverse events (AEs) and serious AEs during the trial period, monitored by clinical laboratory tests (i.e. full blood count, urinalysis, and liver and kidney function tests) (S1 Table), vital signs (i.e. heart rate, body temperature, respiratory rate and blood pressure), and physical examinations at weeks 0, 8 and 20. The total number of AEs of both groups will be counted, with each AE standardized using preferred terms recommended by the Medical Dictionary for Regulatory Activities (MedDRA) version 22.0 [52]. The severity of AEs will be categorized based on the Common Terminology Criteria for Adverse Events (CTCAE) version 5.0 [53]. The causality of AEs will be determined according to the WHO-UMC system [54].

#### Data collection methods

Data will be collected and recorded in case report forms from participants (S7 File). Eligible participants will attend face-to-face assessments at the dermatology outpatient department of GPHCM at eight time points: weeks 0, 2, 4, 6, 8, 12, 16 and 20. The trial investigators will consistently inquire and remind each participant about the specific details of bath administration during each follow-up timepoint throughout the treatment phase. Participants will also need to return trial medications and complete patient-reported outcomes forms during treatment visits (S8 File), as well as complete home diaries during the RCT (S6 and S9 Files). The schedule of data collection is outlined in Fig 1.

Participants will visit the hospital at an appointed time, and be exempt from consultation and examination fees. They may withdraw from the study at any time for any reason. If they wish to withdraw, they will be invited to complete outcome assessments and provide medication usage information.

#### Data management

All physicians, outcome assessors and research assistants will receive training about outcome assessments before the trial starts. Trial investigators will be given a written protocol and required to follow standard operation procedures outlined in the protocol. All data will be entered into a pre-designed, password-protected dataset by personnel blinded to group allocation. Data will be entered regularly throughout the RCT, using a double-checking method. Any corrections to data written in the case report forms will be initialed and dated. The dataset will be locked after all data has been cleaned. Participants’ personal information will be de-identified using unique study codes. A document linking the study codes to the participants’ identities will be stored as a password-protected file. Only study investigators will have access to the passwords. Research data will be kept for a minimum of 15 years after publication of journal articles [55].

#### Data analyses

*Trial feasibility*. Feasibility outcomes will be analyzed both quantitatively and qualitatively. Data related to feasibility outcomes will be collected throughout the trial and the qualitative research, involving keeping records of recruitment numbers, tracking adherence to the intervention protocol, documenting participant retention, and gathering feedback from participants regarding their acceptability and satisfaction of the trial.

The recruitment rate, time required to recruit the target number of participants, frequency of missing data, and participants’ adherence will be calculated and compared with previously published RCTs on psoriasis. This analysis will help in modifying and implementing a future full-scale trial. The credibility of blinding, based on participants’ guesses of group allocation, and participants’ satisfaction with the trial will be analyzed using a *Chi*-square test.

Furthermore, participants’ feedback on the pilot RCT and the CHM bath therapies will be collected through a qualitative study. The data analysis methods for the qualitative study are described in the section on “Embedded Qualitative Research”.

*Analyses on efficacy and safety data*. An independent statistician will perform statistical analyses using PASW Statistics 25.0 software (IBM SPSS Inc, Armonk, New York, USA). Participants’ demographic data and other baseline characteristics will be summarized and compared between two groups. Efficacy data analyses will be based on an intention-to-treat (ITT) population, defined as “participants who have received at least one session of trial medication and one efficacy assessment”. Missing data will be replaced by a last observation carried forward approach. Safety evaluation will be based on a safety-analysis-set population, defined as “participants who have received at least one session of trial medication”.

Statistical tests will be two-sided, with a *P* value of < 0.05 considered statistically significant. Dichotomous data (i.e., PGA, PASI-75, PASI-50 at week 8, responder rate at week 20) will be presented using frequencies and percentages, and compared using a *Chi*-square test or Fisher’s exact test. Continuous variables will be presented as mean and standard deviation if data are normally distributed; or median and inter-quartile range if data are not normally distributed. The superiority comparison between two groups will be presented by 95% confidence interval method. T-test, Mann-Whitney U test or analysis of covariance (ANCOVA) will be used to compare continuous variables between two groups, including the changes of PASI, BSA, VAS, DLQI, and Skindex-16, as well as the medication usage data. When baseline data are balanced, an independent t-test will be used if data are normally distributed; or a Mann-Whitney U test will be used if data are not normally distributed. ANCOVA will be used to adjust for baseline, if baseline imbalance occurs. The percentage changes of PASI, BSA, VAS, DLQI, and Skindex-16 will be presented to indicate clinically relevant information.

Regarding to the safety data, the frequency of AEs and serious AEs will be compared between the two groups using a *Chi*-square test or Fisher’s exact test. In addition, a McNemar test will be employed to compare the severity of AEs categorized by CTCAE before and after treatment within each group.

#### Sample size calculation for the pilot RCT

This pilot study aims to assess the feasibility of the trial design, as well as to provide preliminary data for sample size calculation in a future full-scale trial. We acknowledge the significance of sample size justification in clinical trial planning, not only for the main trial but also for any preliminary pilot trial. However, it is important to consider that pilot trials often have imprecise estimates of the standard deviation parameter [56]. Previous research suggests approximate rules for sample size calculation in pilot trials when the standardized effect size is unknown. According to these rules, pilot trial sample sizes per treatment arm are proposed as 75, 25, 15, and 10 for standardized effect sizes that are extra small (≤ 0.1), small (0.2), medium (0.5), or large (0.8), respectively [56]. Additionally, another research recommended that ‘it is advisable in many circumstances at a high level of confidence to recruit at least 50 participants in a two-arm pilot RCT’ [57].

Considering our clinical observations and adopting a conservative approach, we have determined that a sample size of 25 participants per group is required for this pilot trial. Taking into account a potential 15% loss to follow-up, the pilot study aims to recruit a total of 60 participants.

#### Trial monitoring and quality control

The Data Monitoring Committee from GPHCM will consist of independent experts in clinical pharmacology, dermatology and statistics. This committee will assess the safety data and critical efficacy outcomes, and make recommendations about continuing, modifying or terminating the study. The trial investigator will regularly check data quality. Auditors from the Guangdong International Clinical Research Centre of Chinese Medicine (Guangzhou, China) will oversee data quality throughout the RCT. The Department of Science and Research at GPHCM and the Department of Science and Technology of Guangdong Province in China will audit and inspect the trial.

### Embedded qualitative research

Participants who have successfully completed the pilot RCT and filled out the acceptability questionnaire will be invited for interview as the embedded qualitative study. To ensure diversity among participants, purposive sampling will be employed based on factors such as age, gender, marital state, education level, employment status, geographical location, history and severity of psoriasis vulgaris, comorbidity, response to treatment, AEs, treatment adherence and satisfaction scores collected from the pilot RCT. The allocation of participants into experimental groups will remain unknown during the selection process. It is anticipated that approximately 30 participants will be recruited for the qualitative study, although the final number will be determined by data saturation, which occurs when no new information is obtained from further interviews [58]. All participants will be fully informed about this qualitative study and will provide informed consent prior to their inclusion (S10 File).

Interviews can be conducted through teleconference or face-to-face in a quiet clinic room at GPHCM, depending on participants’ preference. These interviews will take place within four weeks of the participants completing the pilot RCT. To ensure a structured approach, we will develop an interview guide based on a review of relevant academic literature that addresses similar questions (S11 File) [59–63]. Before using the guide, we will pilot it to ensure its effectiveness. During the interviews, participants will be encouraged to freely express their experience with CHM bath therapy for psoriasis vulgaris and their involvement in the pilot RCT. The interview guide and questions will be used flexibly to accommodate the pace and experiences of each participant. Each interview is expected to last between 1 and 1.5 hours. We will take notes during the interviews and summarize them at the conclusion of each session. With the participants’ consent, the interviews will be audio-recorded and transcribed verbatim.

We will utilize thematic analysis based on an inductive approach to analyze qualitative data [64, 65]. This analytical approach consists of six phases [66]: 1) familiarization: reading and re-reading transcripts and field notes to gain familiarity with the data; 2) generating codes; 3) constructing candidate themes; 4) revising themes; 5) defining themes; and 6) producing the report. By following this process, a series of themes and sub-themes will be generated based on grounded theory. All interviews will be transcribed in full and verbatim in the original language (Chinese). The transcripts will be de-identified, coded and uploaded to NVivo 12 software (QSR International Version 12, 2018) for data management and data coding [67]. Line-by-line coding will be used to label themes in the transcripts. Preliminary data analysis will be conducted to determine if any revisions to the interview guide are necessary after each interview.

### Ethics and dissemination

The present study has received ethics approval from the Ethics Committee of GPHCM (approval number: BF2022-189-01) on 22 July 2022 and has been registered with the RMIT University Human Ethics Advisory Network (review reference: 2022-25746-18453) on 01 September 2022. Identifier of the protocol date and version is 20220708/001 approved by the ethics committee (S12 File). The study adheres to the ethical principles outlined in the Declaration of Helsinki, Ethical Guidelines for Medical Research on Humans, and Good Clinical Practice guidelines. The study protocol has been registered with the Chinese Clinical Trial Registry (No. ChiCTR2200064092) on 26 September 2022. Any necessary modifications to the protocol will require formal amendment, which must first be approved by the Ethics Committee of GPHCM prior to implementation. The pilot study protocol and results will be disseminated through peer-reviewed journals.

## Discussion

Psoriasis vulgaris is a prevalent, chronic, dermatological inflammatory disease. The FZHFZY formula has been externally used as a bath therapy for the management of psoriasis in a real-world clinical setting. A pre-clinical study identified 13 bioactive compounds from FZHFZY using the method of three cell lines fishing combined with liquid chromatography-mass spectrometry analysis. It showed that rehmannioside D, rehmannioside A, astilbin and neoisoastilbin of these 13 bioactive compounds could significantly suppress HUVEC cells migration compared with control, which indicated that they might possess antiangiogenesis activity to manage psoriasis [21]. Furthermore, another experimental study indicated that FZHFZY inhibited proliferation and improved epidermal differentiation in IL-17A/IL-22/IFN-γ/TNF-α-induced HaCaT cells, as well as the formular could regulate epidermal differentiation and inhibit phosphorylation of the Akt/mTORC1/S6K1 pathway in the skin of mice with imiquimod-induced psoriasis [68]. Hence, FZHFZY is a promising external treatment, as shown in clinical observation and pre-clinical research. Rigorously designed RCTs are needed to confirm its efficacy and safety.

We designed this RCT as a double-blinded, placebo-controlled trial to evaluate the efficacy of CHM as a bath therapy. A placebo should be designed to be indistinguishable from all sensory specification compared with the trial medication without pharmaceutical activity [69]. Chinese herbal medicine preparations carry special macroscopic, sensory characteristics including appearance, color, smell and taste [70, 71]. In this trial, the CHM and placebo are made as granules. They have been tested by the manufactory and proved that they are indistinguishable in terms of the color, appearance, smell, solubility and particle size. This information will be provided to all participants before their treatment begins. In addition, blinding credibility tests will be performed during the trial. The results of blinding assessment will be interpreted using a blinding index [72, 73].

Feasibility and pilot studies are recommended to develop and evaluate complex interventions in the early phase, according to United Kingdom Medical Research Council guidance [74]. Qualitative approaches can help to explore reasons for the findings, examine the appropriateness of the underlying theory, and steer researchers towards interventions more likely to be effective after a trial [75]. Qualitative or mixed methods are increasingly being used within feasibility studies for RCTs to explore whether researchers could recruit participants to a trial and measure participant acceptance of the intervention [76]. Therefore, this study is designed as a mixed-method feasibility study, consisting of a pilot RCT with qualitative interviews embedded.

In assessing the severity of psoriasis and the response to treatment, PASI, BSA, and PGA are the most commonly used clinical outcome measures [77]. The BSA and PGA are recommended in clinical practice, while PASI is the most widely used tool in clinical trials [7]. Therefore, we selected PASI as the primary efficacy outcome measure, and PGA and BSA as the secondary outcome measures. PASI-75 and PASI-50 will be calculated, since current clinical guidelines commonly use PASI-75 as the goal and PASI-50 as the minimum target of psoriasis treatments [46, 78]. It should be noted that, although the percentage change from baseline is frequently reported in clinical trials, this outcome is considered statistically inefficient [79]. It has been advised that trialists wishing to report percentage change should first using another statistical analysis method, such as ANCOVA, to test significance and calculate confidence intervals, and then convert results to percentage change by using baseline mean and post-treatment scores [79].

Relapse is defined as a loss of 50% of PASI improvement from baseline in patients who achieve PASI-50 at the end of treatment [51]. Psoriasis relapse is a major concern about current therapies, because psoriasis lesions often reoccur after treatment ends [80, 81]. A previous systematic review suggested that using CHM externally in conjunction with Western medicine therapies could reduce the relapse rate of psoriasis, however, no definite conclusion could be made due to the low quality of studies included in the review [18]. In this study, we will assess the relapse rate to explore the long-term effects of FZHFZY. The DLQI is most often used to measure QoL of psoriasis [7], however, it lacks sensitivity in mild symptoms [82–84]. In this case, Skindex-16 (a single-page version developed from Skindex-29) focusing on how much patients are bothered will be used to supplement the QoL data [50, 85, 86]. Itch VAS is the most commonly used pruritus assessment tool, with 60–90% of people with psoriasis experiencing pruritus [87, 88]. The above-mentioned instruments have reproducible inter-rater and intra-rater reliability and validity [45, 47, 48, 77, 84, 88–97]. Therefore, they will be used as secondary efficacy outcomes in this trial.

Psoriasis is an inflammatory dermatosis characterized by increased trans-epidermal water loss, which is a marker of permeability barrier function [98–100]. Basic skin care improves epidermal barrier function and can be used as an essential method for psoriasis treatment [101]. Non-medicated moisturizers are available in several formulations (e.g., creams, ointments, lotions and gels). They can be used as part of a general treatment regimen for patients with psoriasis to help reduce itching and desquamation [7]. There are no known contraindications for these moisturizers, unless there is hypersensitivity to their ingredients [7]. Urea cream is a common non-medicated moisturizer, usually used as an additive, especially in cases of significant scaling, as it facilitates penetration of topical anti-inflammatory agents [101]. Therefore, topical urea cream will be used alongside FZHFZY in this trial, because it not only relieves psoriasis symptoms, which can improve recruitment for the trial, but also will help us to evaluate the effect of FZHFZY as the cream has no active ingredients.

## Conclusion

This pilot study will assess the feasibility of the trial protocol and collect preliminary data on the efficacy and safety of using CHM formula FZHFZY as an additional treatment alongside topical urea cream for mild-to-moderate psoriasis vulgaris. Results from this pilot study will be used to determine the sample size and design of a full-scale RCT.

## Supporting information

S1 Table Abnormal laboratory indicators in terms of the severity of adverse events. (DOCX)

S1 File SPIRIT 2013 checklist. (DOCX)

S2 File Advertisement flyer. (PDF)

S3 File Screening form. (PDF)

S4 File Informed consent form of the pilot RCT. (DOCX)

S5 File Quantitative analysis of active compounds and the HPLC fingerprint of FZHFZY granules. (DOCX)

S6 File Patient home diary during the treatment period. (PDF)

S7 File Case report form. (PDF)

S8 File Patient-reported outcomes form. (PDF)

S9 File Patient home diary during the follow-up period. (PDF)

S10 File Informed consent form of the qualitative study. (DOCX)

S11 File Interview guide and questions. (PDF)

S12 File Study protocol submitted to the ethics committee. (PDF)

S1 Data (PDF)

## Abbreviations

AE: adverse event
ANCOVA: analysis of covariance
BSA: Body Surface Area
CHM: Chinese herbal medicine
CTCAE: Common Terminology Criteria for Adverse Events
DLQI: Dermatology Life Quality Index
FZHFZY: *Fu zheng he fu zhi yang*
GPHCM: Guangdong Provincial Hospital of Chinese Medicine
HPLC: high-performance liquid chromatography
ITT: intention-to-treat
MedDRA: Medical Dictionary for Regulatory Activities
NB-UVB: Narrowband ultraviolet light B
PASI: Psoriasis Area and Severity Index
PASI-50: PASI score decreases more than 50% from baseline
PASI-75: PASI score decreases more than 75% from baseline
PGA: Physician’s Global Assessment
QoL: quality of life
RCT: randomized controlled trial
SPIRIT: Standard Protocol Items: Recommendations for Interventional Trials
VAS: visual analogue scale

## References

[1] Nestle FO, Kaplan DH, Barker J. Psoriasis. N Engl J Med. 2009;361(5):496–509. doi: 10.1056/NEJMra0804595 . 19641206

[2] World Health Organization. Global report on psoriasis. Geneva: World Health Organization; 2016.

[3] Mehrmal S, Uppal P, Giesey RL, Delost GR. Identifying the prevalence and disability-adjusted life years of the most common dermatoses worldwide. J Am Acad Dermatol. 2020;82(1):258–9. Epub 20191001. doi: 10.1016/j.jaad.2019.09.066 . 31585146

[4] Elmets CA, Leonardi CL, Davis DMR, Gelfand JM, Lichten J, Mehta NN, et al. Joint AAD-NPF guidelines of care for the management and treatment of psoriasis with awareness and attention to comorbidities. J Am Acad Dermatol. 2019;80(4):1073–113. Epub 20190213. doi: 10.1016/j.jaad.2018.11.058 . 30772097

[5] Armstrong AW, Read C. Pathophysiology, Clinical Presentation, and Treatment of Psoriasis: A Review. JAMA. 2020;323(19):1945–60. doi: 10.1001/jama.2020.4006 . 32427307

[6] Yeung H, Takeshita J, Mehta NN, Kimmel SE, Ogdie A, Margolis DJ, et al. Psoriasis severity and the prevalence of major medical comorbidity: a population-based study. JAMA Dermatol. 2013;149(10):1173–9. doi: 10.1001/jamadermatol.2013.5015 ; PubMed Central PMCID: PMC3800487. 23925466 PMC3800487

[7] Elmets CA, Korman NJ, Prater EF, Wong EB, Rupani RN, Kivelevitch D, et al. Joint AAD-NPF Guidelines of care for the management and treatment of psoriasis with topical therapy and alternative medicine modalities for psoriasis severity measures. J Am Acad Dermatol. 2021;84(2):432–70. Epub 20200730. doi: 10.1016/j.jaad.2020.07.087 . 32738429

[8] Abraham A, Roga G. Topical steroid-damaged skin. Indian J Dermatol. 2014;59(5):456–9. doi: 10.4103/0019-5154.139872 ; PubMed Central PMCID: PMC4171912. 25284849 PMC4171912

[9] Elmets CA, Lim HW, Stoff B, Connor C, Cordoro KM, Lebwohl M, et al. Joint American Academy of Dermatology-National Psoriasis Foundation guidelines of care for the management and treatment of psoriasis with phototherapy. J Am Acad Dermatol. 2019;81(3):775–804. Epub 20190725. doi: 10.1016/j.jaad.2019.04.042 . 31351884

[10] Almutawa F, Thalib L, Hekman D, Sun Q, Hamzavi I, Lim HW. Efficacy of localized phototherapy and photodynamic therapy for psoriasis: a systematic review and meta-analysis. Photodermatol Photoimmunol Photomed. 2015;31(1):5–14. Epub 20131220. doi: 10.1111/phpp.12092 . 24283358

[11] Zhang CS, Yu J. Psoriasis Vulgaris. Charlie Changli Xue, Lu C, editors: World Scientific; 2017.

[12] Duan X, Cheng Y, Chu L. Efficacy and safety of Chinese herb bath plus narrow-band UVB for psoriasis vulgaris: a systematic review. Chin J Dermatovenereol. 2013;27:192–5.

[13] Guan J, Yuan S, Wu H, Na R, Wu X, Wang X, et al. Effectiveness and safety of traditional Chinese medical bath therapy combined with ultraviolet irradiation in the treatment of psoriasis: A systematic review and meta-analysis of randomized controlled trials. PLoS One. 2017;12(3):e0173276. Epub 20170321. doi: 10.1371/journal.pone.0173276 ; PubMed Central PMCID: PMC5360218. 28323822 PMC5360218

[14] Wu Y, Gao Y, Zhu X. Efficacy and safety evaluation of traditional Chinese medicine bath combined with NB-UVB in the treatment of psoriasis vulgaris. Asia Pac Tradit Med. 2019;15:167–72.

[15] Xu J, Yang M, Chen L. A meta-analysis of the efficacy and quality of life of Chinese herb bath in the treatment of psoriasis vulgaris. Lishizhen Med Mater Med Res. 2019;30:2028–32.

[16] Yu JJ, Zhang CS, Zhang AL, May B, Xue CC, Lu C. Add-on effect of chinese herbal medicine bath to phototherapy for psoriasis vulgaris: a systematic review. Evid Based Complement Alternat Med. 2013;2013:673078. Epub 20130725. doi: 10.1155/2013/673078 ; PubMed Central PMCID: PMC3745880. 23983796 PMC3745880

[17] Lei H, Chen M, Zhang N, Guo X, Yuan X, Tang L, et al. A systematic review and meta-analysis on the efficacy and safety of traditional Chinese medicine bath in the treatment of psoriasis vulgaris. Ann Palliat Med. 2021;10(10):10674–83. doi: 10.21037/apm-21-2386 . 34763428

[18] Yi L, Xun LI, Zi W, Xiaoran Z, Haiyan H, Lingling LI. Efficacy and safety of external application of Chinese herbal medicine for psoriasis vulgaris: a systematic review of randomized controlled trials. J Tradit Chin Med. 2022;42(4):493–504. doi: 10.19852/j.cnki.jtcm.20220617.001 . 35848965 PMC9924682

[19] Guo J, Liu J. Effect of white mange mixture in a murine model of psoriasis. Exp Ther Med. 2019;18(2):881–7. Epub 20190604. doi: 10.3892/etm.2019.7641 ; PubMed Central PMCID: PMC6639981. 31384318 PMC6639981

[20] Chiang CC, Cheng WJ, Lin CY, Lai KH, Ju SC, Lee C, et al. Kan-Lu-Hsiao-Tu-Tan, a traditional Chinese medicine formula, inhibits human neutrophil activation and ameliorates imiquimod-induced psoriasis-like skin inflammation. J Ethnopharmacol. 2020;246:112246. Epub 20190917. doi: 10.1016/j.jep.2019.112246 . 31539577

[21] Chen L, Chen H, Lu Y, Han L, Wang S, Liu M, et al. Decoding active components in a formulation of multiple herbs for treatment of psoriasis based on three cell lines fishing and liquid chromatography-mass spectrometry analysis. J Pharm Biomed Anal. 2020;186:113331. Epub 20200425. doi: 10.1016/j.jpba.2020.113331 . 32380350

[22] Li T, Boutron I, Al-Shahi Salman R, Cobo E, Flemyng E, Grimshaw JM, et al. Review and publication of protocol submissions to Trials—what have we learned in 10 years? Trials. 2016;18(1):34. Epub 2017/01/25. doi: 10.1186/s13063-016-1743-0 ; PubMed Central PMCID: PMC5256548. 28114958 PMC5256548

[23] Chan AW, Tetzlaff JM, Altman DG, Laupacis A, Gøtzsche PC, Krleža-Jerić K, et al. SPIRIT 2013 statement: defining standard protocol items for clinical trials. Ann Intern Med. 2013;158(3):200–7. doi: 10.7326/0003-4819-158-3-201302050-00583 ; PubMed Central PMCID: PMC5114123. 23295957 PMC5114123

[24] Scottish Intercollegiate Guidelines Network. Diagnosis and management of psoriasis and psoriatic arthritis in adults Edinburgh: SIGN; 2010. Available from: http://www.sign.ac.uk.

[25] Committee on Psoriasis, Chinese Society of Dermatology. Guideline for the diagnosis and treatment of psoriasis in China (2018 complete edition). Chin J Dermatol. 2019;(10):667–710.

[26] Liu Y, Liu Y, Du Z, Zhang L, Chen J, Shen Z, et al. Skin microbiota analysis-inspired development of novel anti-infectives. Microbiome. 2020;8(1):85. Epub 20200605. doi: 10.1186/s40168-020-00866-1 ; PubMed Central PMCID: PMC7275423. 32503672 PMC7275423

[27] Menter A, Gelfand JM, Connor C, Armstrong AW, Cordoro KM, Davis DMR, et al. Joint American Academy of Dermatology-National Psoriasis Foundation guidelines of care for the management of psoriasis with systemic nonbiologic therapies. J Am Acad Dermatol. 2020;82(6):1445–86. Epub 20200228. doi: 10.1016/j.jaad.2020.02.044 . 32119894

[28] Menter A, Strober BE, Kaplan DH, Kivelevitch D, Prater EF, Stoff B, et al. Joint AAD-NPF guidelines of care for the management and treatment of psoriasis with biologics. J Am Acad Dermatol. 2019;80(4):1029–72. Epub 20190213. doi: 10.1016/j.jaad.2018.11.057 . 30772098

[29] Warren RB, Mrowietz U, von Kiedrowski R, Niesmann J, Wilsmann-Theis D, Ghoreschi K, et al. An intensified dosing schedule of subcutaneous methotrexate in patients with moderate to severe plaque-type psoriasis (METOP): a 52 week, multicentre, randomised, double-blind, placebo-controlled, phase 3 trial. Lancet. 2017;389(10068):528–37. Epub 20161222. doi: 10.1016/S0140-6736(16)32127-4 . 28012564

[30] Chinese Society of Dermatology, China Dermatologist Association, Dermatology and Venereology Specialized Committee of Chinese Association of Integrative Medicine. Guidelines for the treatment of psoriasis with biologic agents in China (2021). Chin J Dermatol. 2021;54(12):1033–47.

[31] Zhang Q, Shi H, Zhang J, Jiang C, Zhou C. The paeonol target gene autophagy-related 5 has a potential therapeutic value in psoriasis treatment. PeerJ. 2021;9:e11278. Epub 2021/06/12. doi: 10.7717/peerj.11278 ; PubMed Central PMCID: PMC8162242. 34113484 PMC8162242

[32] Meng Y, Wang M, Xie X, Di T, Zhao J, Lin Y, et al. Paeonol ameliorates imiquimod-induced psoriasis-like skin lesions in BALB/c mice by inhibiting the maturation and activation of dendritic cells. Int J Mol Med. 2017;39(5):1101–10. Epub 2017/03/25. doi: 10.3892/ijmm.2017.2930 ; PubMed Central PMCID: PMC5403289. 28339016 PMC5403289

[33] Xu Q, Liu Z, Cao Z, Shi Y, Yang N, Cao G, et al. Topical astilbin ameliorates imiquimod-induced psoriasis-like skin lesions in SKH-1 mice via suppression dendritic cell-Th17 inflammation axis. J Cell Mol Med. 2022;26(4):1281–92. Epub 2022/01/14. doi: 10.1111/jcmm.17184 ; PubMed Central PMCID: PMC8831981. 35023281 PMC8831981

[34] Wang W, Yuhai, Wang H, Chasuna Bagenna. Astilbin reduces ROS accumulation and VEGF expression through Nrf2 in psoriasis-like skin disease. Biol Res. 2019;52(1):49. Epub 2019/09/08. doi: 10.1186/s40659-019-0255-2 ; PubMed Central PMCID: PMC6729080. 31492195 PMC6729080

[35] Cui B, Sun Y, Liu W, Liao G. Clinical Efficacy of Narrow Band Ultraviolet B in Combined with Yuyin Recipe in Treating Psoriasis Vulgaris. Chinese Journal of Integrated Traditional and Western Medicine. 2008;(04):355–7. 18543493

[36] Li N. Clinical observation of Yuyin formula combined with NB-UVB for psoriasis vulgaris. China Health Industry. 2014;11(20):190–1. doi: 10.16659/j.cnki.1672-5654.2014.20.097

[37] Wang H, Hu Y, Li T, Yu A, Zhang H, Chen L. Efficacy of traditional Chinese medicine bath combined with narrow band ultraviolet B and its relation with serum 25-hydroxyvitamin D in psoriasis vulgaris of blood heat syndrome. Hebei Journal of Traditional Chinese Medicine. 2022;44(01):47–50.

[38] Wu B, Chen X, Xia D, Chen L, Lu Y, Zhang D, et al. Clinical observation of Chinese herbal medicine bath therapy combined with NB-UVB for psoriasis vulgaris. Chinese Journal of Dermatovenereology of Integrated Traditional and Western Medicine. 2011;10(05):304–5.

[39] Zhang J, Liu G, Zhang X, Li X, Zhou L, Wang C, et al. Clinical Observation on Treatment of Psoriasis Vulgaris at Resting Stage with Tonghe Medicated Bath Prescription Combined with NB-UVB. Chinese Journal of Dermatovenereology of Integrated Traditional and Western Medicine. 2019;18(05):417–20.

[40] Long CC, Finlay AY. The finger-tip unit—a new practical measure. Clin Exp Dermatol. 1991;16(6):444–7. doi: 10.1111/j.1365-2230.1991.tb01232.x . 1806320

[41] Finlay AY, Edwards PH, Harding KG. "Fingertip unit" in dermatology. Lancet. 1989;2(8655):155. doi: 10.1016/s0140-6736(89)90204-3 . 2567912

[42] Bowen DJ, Kreuter M, Spring B, Cofta-Woerpel L, Linnan L, Weiner D, et al. How we design feasibility studies. Am J Prev Med. 2009;36(5):452–7. doi: 10.1016/j.amepre.2009.02.002 ; PubMed Central PMCID: PMC2859314. 19362699 PMC2859314

[43] Thabane L, Ma J, Chu R, Cheng J, Ismaila A, Rios LP, et al. A tutorial on pilot studies: the what, why and how. BMC Med Res Methodol. 2010;10:1. Epub 20100106. doi: 10.1186/1471-2288-10-1 ; PubMed Central PMCID: PMC2824145. 20053272 PMC2824145

[44] Kleiss I, Kortlever J, Karyampudi P, Ring D, Brown L, Reichel L, et al. A Comparison of 4 Single-Question Measures of Patient Satisfaction. Journal of clinical outcomes management: JCOM. 2020;27:41–8.

[45] Berth-Jones J, Grotzinger K, Rainville C, Pham B, Huang J, Daly S, et al. A study examining inter- and intrarater reliability of three scales for measuring severity of psoriasis: Psoriasis Area and Severity Index, Physician’s Global Assessment and Lattice System Physician’s Global Assessment. Br J Dermatol. 2006;155(4):707–13. doi: 10.1111/j.1365-2133.2006.07389.x . 16965419

[46] Nast A, Boehncke WH, Mrowietz U, Ockenfels HM, Philipp S, Reich K, et al. S3—Guidelines on the treatment of psoriasis vulgaris (English version). Update. J Dtsch Dermatol Ges. 2012;10 Suppl 2:S1–95. doi: 10.1111/j.1610-0387.2012.07919.x . 22386073

[47] Bożek A, Reich A. The reliability of three psoriasis assessment tools: Psoriasis area and severity index, body surface area and physician global assessment. Adv Clin Exp Med. 2017;26(5):851–6. doi: 10.17219/acem/69804 . 29068583

[48] Phan NQ, Blome C, Fritz F, Gerss J, Reich A, Ebata T, et al. Assessment of pruritus intensity: prospective study on validity and reliability of the visual analogue scale, numerical rating scale and verbal rating scale in 471 patients with chronic pruritus. Acta Derm Venereol. 2012;92(5):502–7. doi: 10.2340/00015555-1246 . 22170091

[49] Mazzotti E, Picardi A, Sampogna F, Sera F, Pasquini P, Abeni D. Sensitivity of the Dermatology Life Quality Index to clinical change in patients with psoriasis. Br J Dermatol. 2003;149(2):318–22. doi: 10.1046/j.1365-2133.2003.05378.x . 12932238

[50] Chren MM. The Skindex instruments to measure the effects of skin disease on quality of life. Dermatol Clin. 2012;30(2):231–6, xiii. Epub 20111220. doi: 10.1016/j.det.2011.11.003 ; PubMed Central PMCID: PMC3269028. 22284137 PMC3269028

[51] Carey W, Glazer S, Gottlieb AB, Lebwohl M, Leonardi C, Menter A, et al. Relapse, rebound, and psoriasis adverse events: an advisory group report. J Am Acad Dermatol. 2006;54(4 Suppl 1):S171–81. doi: 10.1016/j.jaad.2005.10.029 . 16488339

[52] The International Council for Harmonisation of Technical Requirements for Pharmaceuticals for Human Use (ICH) organisation. The Medical Dictionary for Regulatory Activities (MedDRA) system organ classes 2016. Available from: https://www.meddra.org/About%20MedDRA%20/%20Evolution%20/%2027th-system-organ-class.

[53] U.S. DEPARTMENT OF HEALTH AND HUMAN SERVICES. Common Terminology Criteria for Adverse Events (CTCAE) Version 5.0 November 27, 2017. Available from: https://ctep.cancer.gov/protocoldevelopment/electronic_applications/docs/ctcae_v5_quick_reference_5x7.pdf.

[54] World Health Organization. The use of the WHO-UMC system for standardised case causality assessment 2013. Available from: https://www.who.int/publications/m/item/WHO-causality-assessment.

[55] National Health and Medical Research Council, Australian Research Council, Universities Australia. Australian code for the responsible conduct of research Australia 2018 [cited 2022 November 16]. Available from: https://www.nhmrc.gov.au/about-us/publications/australian-code-responsible-conduct-research-2018.

[56] Whitehead AL, Julious SA, Cooper CL, Campbell MJ. Estimating the sample size for a pilot randomised trial to minimise the overall trial sample size for the external pilot and main trial for a continuous outcome variable. Stat Methods Med Res. 2016;25(3):1057–73. Epub 2015/06/21. doi: 10.1177/0962280215588241 ; PubMed Central PMCID: PMC4876429. 26092476 PMC4876429

[57] Sim J, Lewis M. The size of a pilot study for a clinical trial should be calculated in relation to considerations of precision and efficiency. J Clin Epidemiol. 2012;65(3):301–8. Epub 20111209. doi: 10.1016/j.jclinepi.2011.07.011 . 22169081

[58] Morse JM. The Significance of Saturation. Qualitative health research. 1995;5(2):147–9. doi: 10.1177/104973239500500201

[59] Sugg HVR, Frost J, Richards DA. Morita Therapy for depression (Morita Trial): an embedded qualitative study of acceptability. BMJ Open. 2019;9(2):e023873. doi: 10.1136/bmjopen-2018-023873 31147359 PMC6549637

[60] Devall A, Chu J, Beeson L, Hardy P, Cheed V, Sun Y, et al. Mifepristone and misoprostol versus placebo and misoprostol for resolution of miscarriage in women diagnosed with missed miscarriage: the MifeMiso RCT. Health Technology Assessment. 2021;25(68):1–114. doi: 10.3310/hta25680 34821547

[61] Chen X, Jiang W, Lin X, Lundborg CS, Wen Z, Lu W, et al. Effect of an exercise-based cardiac rehabilitation program "Baduanjin Eight-Silken-Movements with self-efficacy building" for heart failure (BESMILE-HF study): study protocol for a randomized controlled trial. Trials. 2018;19:150. doi: 10.1186/s13063-018-2531-9 29490680 PMC5831846

[62] Bobbink P, Larkin PJ, Probst S. Experiences of Venous Leg Ulcer persons following an individualised nurse-led education: protocol for a qualitative study using a constructivist grounded theory approach. BMJ Open. 2020;10:e042605. doi: 10.1136/bmjopen-2020-042605 33243816 PMC7692966

[63] Coyle ME, Yu JJ, Zhang AL, Jones L, Xue CC, Lu C. Patient experiences of using Chinese herbal medicine for psoriasis vulgaris and chronic urticaria: a qualitative study. J Dermatolog Treat. 2020;31(4):352–8. Epub 20190902. doi: 10.1080/09546634.2019.1591580 . 30897010

[64] Chapman AL, Hadfield M, Chapman CJ. Qualitative research in healthcare: an introduction to grounded theory using thematic analysis. The Journal of the Royal College of Physicians of Edinburgh. 2015;45(3):201–5. doi: 10.4997/JRCPE.2015.305 26517098

[65] Braun V, Clarke V. Using thematic analysis in psychology. Qualitative research in psychology. 2006;3(2):77–101. doi: 10.1191/1478088706qp063oa

[66] Liamputtong P. Handbook of research methods in health social sciences. Singapore: Springer; 2019.

[67] Bazeley P, Jackson K. Qualitative data analysis with NVivo. Second Edition. ed. Los Angeles: SAGE; 2013.

[68] Lu Y, Chen H, Zhang J, Tang B, Zhang H, Ma C, et al. Fuzhenghefuzhiyang Formula (FZHFZY) Improves Epidermal Differentiation via Suppression of the Akt/mTORC1/S6K1 Signalling Pathway in Psoriatic Models. Front Pharmacol. 2021;12:650816. Epub 20210811. doi: 10.3389/fphar.2021.650816 ; PubMed Central PMCID: PMC8386017. 34456715 PMC8386017

[69] Laursen DRT, Hansen C, Paludan-Müller AS, Hróbjartsson A. Active placebo versus standard placebo control interventions in pharmacological randomised trials. Cochrane Database of Systematic Reviews. 2020;(7). doi: 10.1002/14651858.MR000055 MR000055. PMC9989326 36877132

[70] Guo N, Wu F, Wu M, Wang Y, Lang Q, Lin X, et al. Progress in the design and quality control of placeboes for clinical trials of traditional Chinese medicine. J Integr Med. 2022;20(3):204–12. Epub 20220221. doi: 10.1016/j.joim.2022.02.005 . 35248517

[71] Qi GD, We DA, Chung LP, Fai CK. Placebos used in clinical trials for Chinese herbal medicine. Recent Pat Inflamm Allergy Drug Discov. 2008;2(2):123–7. doi: 10.2174/187221308784543700 . 19076001

[72] Bang H, Ni L, Davis CE. Assessment of blinding in clinical trials. Control Clin Trials. 2004;25(2):143–56. doi: 10.1016/j.cct.2003.10.016 . 15020033

[73] Freed B, Assall OP, Panagiotakis G, Bang H, Park JJ, Moroz A, et al. Assessing blinding in trials of psychiatric disorders: a meta-analysis based on blinding index. Psychiatry Res. 2014;219(2):241–7. Epub 20140522. doi: 10.1016/j.psychres.2014.05.023 ; PubMed Central PMCID: PMC4183143. 24930582 PMC4183143

[74] Craig P, Dieppe P, Macintyre S, Michie S, Nazareth I, Petticrew M. Developing and evaluating complex interventions: the new Medical Research Council guidance. Bmj. 2008;337:a1655. Epub 20080929. doi: 10.1136/bmj.a1655 ; PubMed Central PMCID: PMC2769032. 18824488 PMC2769032

[75] Lewin S, Glenton C, Oxman AD. Use of qualitative methods alongside randomised controlled trials of complex healthcare interventions: methodological study. Bmj. 2009;339:b3496. Epub 20090910. doi: 10.1136/bmj.b3496 ; PubMed Central PMCID: PMC2741564. 19744976 PMC2741564

[76] Soliman EZ, Mendis S, Dissanayake WP, Somasundaram NP, Gunaratne PS, Jayasingne IK, et al. A Polypill for primary prevention of cardiovascular disease: a feasibility study of the World Health Organization. Trials. 2011;12:3. Epub 20110105. doi: 10.1186/1745-6215-12-3 ; PubMed Central PMCID: PMC3023675. 21205325 PMC3023675

[77] American Academy of Dermatology. Measure 410 (Psoriasis: Clinical Response to Systemic Medications) 2019 [cited 2022 January 20]. Available from: https://www.aad.org/member/practice/mips/measures/410a.

[78] National Institute for Health and Clinical Excellence Guidance. Psoriasis: Assessment and Management of Psoriasis London 2012 [cited 2022 November 16]. Available from: https://www.nice.org.uk/guidance/cg153.

[79] Vickers AJ. The use of percentage change from baseline as an outcome in a controlled trial is statistically inefficient: a simulation study. BMC Med Res Methodol. 2001;1:6. Epub 2001/07/19. doi: 10.1186/1471-2288-1-6 ; PubMed Central PMCID: PMC34605. 11459516 PMC34605

[80] Tian D, Lai Y. The Relapse of Psoriasis: Mechanisms and Mysteries. JID Innov. 2022;2(3):100116. Epub 20220309. doi: 10.1016/j.xjidi.2022.100116 ; PubMed Central PMCID: PMC9121322. 35601055 PMC9121322

[81] Masson Regnault M, Shourick J, Jendoubi F, Tauber M, Paul C. Time to Relapse After Discontinuing Systemic Treatment for Psoriasis: A Systematic Review. Am J Clin Dermatol. 2022:1–15. Epub 20220430. doi: 10.1007/s40257-022-00679-y ; PubMed Central PMCID: PMC9055370. 35489008 PMC9055370

[82] Chren MM, Lasek RJ, Quinn LM, Covinsky KE. Convergent and discriminant validity of a generic and a disease-specific instrument to measure quality of life in patients with skin disease. J Invest Dermatol. 1997;108(1):103–7. doi: 10.1111/1523-1747.ep12285650 . 8980297

[83] Chren MM, Lasek RJ, Flocke SA, Zyzanski SJ. Improved discriminative and evaluative capability of a refined version of Skindex, a quality-of-life instrument for patients with skin diseases. Arch Dermatol. 1997;133(11):1433–40. . 9371029

[84] Chren MM, Lasek RJ, Quinn LM, Mostow EN, Zyzanski SJ. Skindex, a quality-of-life measure for patients with skin disease: reliability, validity, and responsiveness. J Invest Dermatol. 1996;107(5):707–13. doi: 10.1111/1523-1747.ep12365600 . 8875954

[85] Chren MM, Lasek RJ, Sahay AP, Sands LP. Measurement properties of Skindex-16: a brief quality-of-life measure for patients with skin diseases. J Cutan Med Surg. 2001;5(2):105–10. Epub 20010321. doi: 10.1007/BF02737863 . 11443481

[86] Szabó Á, Brodszky V, Rencz F. A comparative study on the measurement properties of Dermatology Life Quality Index (DLQI), DLQI-Relevant and Skindex-16. Br J Dermatol. 2022;186(3):485–95. Epub 20211124. doi: 10.1111/bjd.20765 . 34724199

[87] Komiya E, Tominaga M, Kamata Y, Suga Y, Takamori K. Molecular and Cellular Mechanisms of Itch in Psoriasis. Int J Mol Sci. 2020;21(21):8406. Epub 20201109. doi: 10.3390/ijms21218406 ; PubMed Central PMCID: PMC7664892. 33182442 PMC7664892

[88] Reich A, Heisig M, Phan NQ, Taneda K, Takamori K, Takeuchi S, et al. Visual analogue scale: evaluation of the instrument for the assessment of pruritus. Acta Derm Venereol. 2012;92(5):497–501. doi: 10.2340/00015555-1265 . 22102095

[89] Berth-Jones J, Thompson J, Papp K. A study examining inter-rater and intrarater reliability of a novel instrument for assessment of psoriasis: the Copenhagen Psoriasis Severity Index. Br J Dermatol. 2008;159(2):407–12. Epub 20080628. doi: 10.1111/j.1365-2133.2008.08680.x . 18565187

[90] Cabrera S, Chinniah N, Lock N, Cains GD, Woods J. Inter-observer reliability of the PASI in a clinical setting. Australas J Dermatol. 2015;56(2):100–2. Epub 20150305. doi: 10.1111/ajd.12280 . 25753553

[91] Charman CR, Venn AJ, Williams HC. Measurement of body surface area involvement in atopic eczema: an impossible task? Br J Dermatol. 1999;140(1):109–11. doi: 10.1046/j.1365-2133.1999.02617.x . 10215778

[92] Langley RG, Ellis CN. Evaluating psoriasis with Psoriasis Area and Severity Index, Psoriasis Global Assessment, and Lattice System Physician’s Global Assessment. J Am Acad Dermatol. 2004;51(4):563–9. doi: 10.1016/j.jaad.2004.04.012 . 15389191

[93] Cappelleri JC, Bushmakin AG, Harness J, Mamolo C. Psychometric validation of the physician global assessment scale for assessing severity of psoriasis disease activity. Qual Life Res. 2013;22(9):2489–99. Epub 20130309. doi: 10.1007/s11136-013-0384-y . 23475691

[94] Storck M, Sandmann S, Bruland P, Pereira MP, Steinke S, Riepe C, et al. Pruritus Intensity Scales across Europe: a prospective validation study. J Eur Acad Dermatol Venereol. 2021;35(5):1176–85. Epub 20210203. doi: 10.1111/jdv.17111 . 33411947

[95] Amatya B, Wennersten G, Nordlind K. Patients’ perspective of pruritus in chronic plaque psoriasis: a questionnaire-based study. J Eur Acad Dermatol Venereol. 2008;22(7):822–6. Epub 20080415. doi: 10.1111/j.1468-3083.2008.02591.x . 18422545

[96] Pedersen CB, McHorney CA, Larsen LS, Lophaven KW, Moeller AH, Reaney M. Reliability and validity of the Psoriasis Itch Visual Analog Scale in psoriasis vulgaris. J Dermatolog Treat. 2017;28(3):213–20. Epub 20160905. doi: 10.1080/09546634.2016.1215405 . 27454156

[97] Kimball AB, Naegeli AN, Edson-Heredia E, Lin CY, Gaich C, Nikaï E, et al. Psychometric properties of the Itch Numeric Rating Scale in patients with moderate-to-severe plaque psoriasis. Br J Dermatol. 2016;175(1):157–62. Epub 20160508. doi: 10.1111/bjd.14464 . 26852717

[98] Fölster-Holst R. Eczematous disorders in adolescents. Hautarzt. 2016;67(4):287–92. doi: 10.1007/s00105-016-3764-8 . 26857132

[99] Grice K, Sattar H, Baker H, Sharratt M. The relationship of transepidermal water loss to skin temperature in psoriasis and eczema. J Invest Dermatol. 1975;64(5):313–5. doi: 10.1111/1523-1747.ep12512258 . 1141706

[100] Hagemann I, Proksch E. Topical treatment by urea reduces epidermal hyperproliferation and induces differentiation in psoriasis. Acta Derm Venereol. 1996;76(5):353–6. doi: 10.2340/0001555576353356 . 8891006

[101] Eisert L, Augustin M, Bach S, Dittmann M, Eiler R, Fölster-Holst R, et al. S2k guidelines for the treatment of psoriasis in children and adolescents—Short version part 1. J Dtsch Dermatol Ges. 2019;17(8):856–70. doi: 10.1111/ddg.13907 . 31437363

