# Supplementary material for: Add-on effects of Chinese herbal medicine external application (FZHFZY) to topical urea for mild-to-moderate psoriasis vulgaris: Protocol for a double-blinded randomized controlled pilot trial embedded with a qualitative study
Source: PLoS One. 2024 Mar 21;19(3):e0297834. doi: 10.1371/journal.pone.0297834 (PMC10956750; doi:10.1371/journal.pone.0297834)
Supplement: S1 Table — (DOCX) [file pone.0297834.s001.docx]

**S1 Table. Abnormal laboratory indicators in terms of the severity of adverse events ^a^**

| **Laboratory Test** | **Abnormal laboratory indicator** | **Grade 1** | **Grade 2** | **Grade 3** | **Grade 4** | **Grade 5** |
| --- | --- | --- | --- | --- | --- | --- |
| **Full blood count** | White blood cells decreased | < LLN – 3,000 mm^–3^; < LLN – 3.0 × 10^9^ L^–1^ | < 3,000 – 2,000 mm^–3^; < 3.0 – 2.0 × 10^9^ L^–1^ | < 2,000 – 1,000 mm^–3^; < 2.0 – 1.0 × 10^9^ L^–1^ | < 1,000 mm^–3^; < 1.0 × 10^9^ L^–1^ | NA |
|  | Neutrophil count decreased | < LLN – 1500 mm^–3^; < LLN – 1.5 × 10^9^ L^–1^ | < 1500 – 1000 mm^–3^; < 1.5 – 1.0 × 10^9^ L^–1^ | < 1000 – 500 mm^–3^; < 1.0 – 0.5 × 10^9^ L^–1^ | < 500 mm^–3^; <0.5 × 10^9^ L^–1^ | NA |
|  | Lymphocyte count decreased | < LLN – 800 mm^–3^; < LLN – 0.8 × 10^9^ L^–1^ | < 800 – 500 mm^–3^; < 0.8 – 0.5 × 10^9^ L^–1^ | < 500 – 200 mm^–3^; < 0.5 – 0.2 × 10^9^ L^–1^ | < 200 mm^–3^; < 0.2 × 10^9^ L^–1^ | NA |
|  | Lymphocyte count increased | NA | > 4,000 mm^–3^ – 20,000 mm^–3^ | > 20,000 mm^–3^ | NA | NA |
|  | Hemoglobin decreased | < LLN – 10.0 g/dL; < LLN – 6.2 mmol/L; < LLN – 100 g/L | < 10.0 – 8.0 g/dL; < 6.2 – 4.9 mmol/L; < 100 – 80 g/L | < 8.0 g/dL; < 4.9 mmol/L; < 80 g/L | NA | NA |
|  | Hemoglobin increased | Increase in > 0 – 2 g/dL | Increase in > 2 – 4 g/dL | Increase in > 4 g/dL | NA | NA |
|  | Platelet count decreased | < LLN – 75,000 mm^–3^; < LLN – 75.0 × 10^9^ L^–1^ | < 75,000 – 50,000 mm^–3^; < 75.0 – 50.0 × 10^9^ L^–1^ | < 50,000 – 25,000 mm^–3^; < 50.0 – 25.0 × 10^9^ L^–1^ | < 25,000 mm^–3^; < 25.0 × 10^9^ L^–1^ | NA |
| **Urinalysis** | Proteinuria | 1+ proteinuria; urinary protein ≥ ULN – < 1.0 g/24 hrs | 2+ and 3+ proteinuria; urinary protein 1.0 – < 3.5 g/24 hrs | 4+ proteinuria; urinary protein ≥ 3.5 g/24 hrs | NA | NA |
|  | Glucosuria | Present | NA | NA | NA | NA |
|  | Hemoglobinuria | Asymptomatic; clinical or diagnostic observations only; intervention not indicated | NA | NA | NA | NA |
| **Liver function test** | Alanine aminotransferase increased | > ULN – 3.0 × ULN if baseline was normal; 1.5 – 3.0 × baseline if baseline was abnormal | > 3.0 – 5.0 × ULN if baseline was normal; > 3.0 – 5.0 × baseline if baseline was abnormal | > 5.0 – 20.0 × ULN if baseline was normal; > 5.0 – 20.0 × baseline if baseline was abnormal | > 20.0 × ULN if baseline was normal; > 20.0 × baseline if baseline was abnormal | NA |
|  | Aspartate aminotransferase increased | > ULN – 3.0 × ULN if baseline was normal; 1.5 – 3.0 × baseline if baseline was abnormal | > 3.0 – 5.0 × ULN if baseline was normal; > 3.0 – 5.0 × baseline if baseline was abnormal | > 5.0 – 20.0 × ULN if baseline was normal; > 5.0 – 20.0 × baseline if baseline was abnormal | > 20.0 × ULN if baseline was normal; > 20.0 × baseline if baseline was abnormal | NA |
|  | Gamma-glutamyl transferase increased | > ULN – 2.5 × ULN if baseline was normal; 2.0 – 2.5 × baseline if baseline was abnormal | > 2.5 – 5.0 × ULN if baseline was normal; > 2.5 – 5.0 × baseline if baseline was abnormal | > 5.0 – 20.0 × ULN if baseline was normal; > 5.0 – 20.0 × baseline if baseline was abnormal | > 20.0 × ULN if baseline was normal; > 20.0 × baseline if baseline was abnormal | NA |
| **Kidney function test** | Creatinine increased | > ULN – 1.5 × ULN | > 1.5 – 3.0 × baseline; > 1.5 – 3.0 × ULN | > 3.0 × baseline; > 3.0 – 6.0 × ULN | > 6.0 × ULN | NA |
|  | eGFR (estimated Glomerular Filtration Rate) | < LLN – 60 mL/min/1.73 m^2^ | 59 – 30 mL/min/1.73 m^2^ | 29 – 15 mL/min/1.73 m^2^ | < 15 mL/min/1.73 m^2^ | NA |

LLN, lower limit of normal; NA, not available; ULN, upper limit of normal.

**^a^** Grade refers to the severity of the adverse event. The CTCAE 5.0 (Common Terminology Criteria for Adverse Events, version 5.0) displays Grades 1 through 5 with unique clinical descriptions of severity for each adverse event based on this general guideline:

- Grade 1 Mild; asymptomatic or mild symptoms; clinical or diagnostic observations only; intervention not indicated.
- Grade 2 Moderate; minimal, local or non-invasive intervention indicated; limiting age-appropriate instrumental Activities of Daily Living (ADL)*.
- Grade 3 Severe or medically significant but not immediately life-threatening; hospitalization or prolongation of hospitalization indicated; disabling; limiting self-care ADL**.
- Grade 4 Life-threatening consequences; urgent intervention indicated.
- Grade 5 Death related to AE.

Activities of Daily Living (ADL)

*Instrumental ADL refer to preparing meals, shopping for groceries or clothes, using the telephone, managing money, etc.

**Self-care ADL refer to bathing, dressing and undressing, feeding self, using the toilet, taking medications, and not bedridden.
