# Supplementary material for: Add-on effects of Chinese herbal medicine external application (FZHFZY) to topical urea for mild-to-moderate psoriasis vulgaris: Protocol for a double-blinded randomized controlled pilot trial embedded with a qualitative study
Source: PLoS One. 2024 Mar 21;19(3):e0297834. doi: 10.1371/journal.pone.0297834 (PMC10956750; doi:10.1371/journal.pone.0297834)
Supplement: S2 File — (PDF) [file pone.0297834.s003.pdf]

S2 File. Advertisement flyer

Researchers at the RMIT University and the Guangdong Provincial Hospital of Chinese Medicine (GPHCM) are conducting a clinical trial entitled “Add-on Chinese herbal medicine external application (FZHFZY) to topical urea for mild-to-moderate psoriasis vulgaris: a pilot randomised controlled trial embedded with a qualitative interview”. This clinical trial has been approved by the Ethics Committee of GPHCM (No. BF 2022-189-01) and registered with the RMIT University Human Ethics Advisory Network (No. 2022-25746-18453). This trial will be conducted in the dermatology outpatient clinic at the GPHCM. Now, we are looking for volunteers with psoriasis vulgaris to participate in the trial.

You may participate in this trial if you are diagnosed with mild to moderate psoriasis vulgaris and your age is between 18 and 65 years. However, if you meet any of the exclusion criteria, you will not be included because it may cause potential risks: 1) currently are pregnant and lactating, 2) have uncontrolled or severe diseases, 3) are allergic to the medications used in this study; 4) currently are participating in or have participated in other clinical trial(s) in the previous month; 5) are not able or unwilling to stop using other treatments for psoriasis during the trial. If you or your friends are interested in the clinical trial, please contact us. We will assess your condition and let you know if you are eligible for the trial.

Dr. Junyue Wang

Ph:

Research organisations: RMIT University & Guangdong Provincial Hospital of Chinese Medicine

Address: No. 111, Dade Road, Yuexiu District, Guangzhou, Guangdong Province

Date:
