# Supplementary material for: Add-on effects of Chinese herbal medicine external application (FZHFZY) to topical urea for mild-to-moderate psoriasis vulgaris: Protocol for a double-blinded randomized controlled pilot trial embedded with a qualitative study
Source: PLoS One. 2024 Mar 21;19(3):e0297834. doi: 10.1371/journal.pone.0297834 (PMC10956750; doi:10.1371/journal.pone.0297834)
Supplement: S3 File — (PDF) [file pone.0297834.s004.pdf]

S3 File. Screening form

Screening form

Serial number of screening: |__|__|__|

ID card of GPHCM: |__|__|__|__||__|__|__|__||__|__|__|

<table>
<tr><td>Name</td><td></td><td>Gender</td><td colspan="2">☐Male ☐Female</td><td>Date of birth</td><td colspan="2"></td></tr>
<tr><td>Address</td><td colspan="4"></td><td>Telephone</td><td colspan="2"></td></tr>
<tr><td colspan="3">PASI score Pre-washout period: ___________ After washout period: ___________</td><td colspan="5">BSA score Pre-washout period: ___________ After washout period: ___________</td></tr>
<tr><td rowspan="4">Skin lesions</td><td colspan="5">Erythematous plaques</td><td>☐Yes</td><td>☐No</td></tr>
<tr><td colspan="5">Silvery scales</td><td>☐Yes</td><td>☐No</td></tr>
<tr><td colspan="5">Candle wax phenomenon</td><td>☐Yes</td><td>☐No</td></tr>
<tr><td colspan="5">Auspitz</td><td>☐Yes</td><td>☐No</td></tr>
<tr><td>Pathological diagnosis</td><td colspan="5">The pathological diagnosis was psoriasis</td><td>☐Yes</td><td>☐No</td></tr>
</table>

Run-in the wash-out period:

☐Yes, pervious psoriasis therapies:

☐Topical agents:____________, Cease: ☐No, ☐Yes,_______days

☐Systemic nonbiologic therapies:_____________, Cease: ☐No, ☐Yes,_______days

☐Phototherapies:____________, Cease: ☐No, ☐Yes,_______days

☐Systemics:________________, Cease: ☐No, ☐Yes,_______days

☐Other:________________, Cease: ☐No, ☐Yes,_______days

Wash-out requirements: period:___________days, the end date: ________________

☐No

☐Run-in without the washout period

Signature of the researcher: ________________ **Date:** ___________________

Pre-washout period assessment

Psoriasis Area and Severity Index, PASI				
	**Head (h)**	**Upper limbs (u)**	**Trunk (t)**	**Lower limbs (l)**
**Erythema (E)**	☐0 ☐1 ☐2 ☐3 ☐4	☐0 ☐1 ☐2 ☐3 ☐4	☐0 ☐1 ☐2 ☐3 ☐4	☐0 ☐1 ☐2 ☐3 ☐4
**Thickness (D)**	☐0 ☐1 ☐2 ☐3 ☐4	☐0 ☐1 ☐2 ☐3 ☐4	☐0 ☐1 ☐2 ☐3 ☐4	☐0 ☐1 ☐2 ☐3 ☐4
**Scaling (I)**	☐0 ☐1 ☐2 ☐3 ☐4	☐0 ☐1 ☐2 ☐3 ☐4	☐0 ☐1 ☐2 ☐3 ☐4	☐0 ☐1 ☐2 ☐3 ☐4
**Lesion Score Sum (A)**	☐0 = 0%<br>☐1 = 1–9%<br>☐2 = 10–29%<br>☐3 = 30–49%<br>☐4 = 50–69%<br>☐5 = 70–89%<br>☐6 = 90–100%	☐0 = 0%<br>☐1 = 1–9%<br>☐2 = 10–29%<br>☐3 = 30–49%<br>☐4 = 50–69%<br>☐5 = 70–89%<br>☐6 = 90–100%	☐0 = 0%<br>☐1 = 1–9%<br>☐2 = 10–29%<br>☐3 = 30–49%<br>☐4 = 50–69%<br>☐5 = 70–89%<br>☐6 = 90–100%	☐0 = 0%<br>☐1 = 1–9%<br>☐2 = 10–29%<br>☐3 = 30–49%<br>☐4 = 50–69%<br>☐5 = 70–89%<br>☐6 = 90–100%
**Palm areas (one palm ≈ 1% of BSA)**	one palm ≈ 11.1% of head and neck area	one palm ≈ 5.6% of the upper extremities	1 palm ≈ 3.7% of the trunk	1 palm ≈ 2.2% of lower extremities
	______Palms	______Palms	______Palms	______Palms
**BSA**	% (palms of h + palms of u + palms of t + palms of l)			
**Subtotals**	0.1 * Ah (Eh + Ih + Dh) =	0.2 * Au (Eu + Iu + Du) =	0.3 * At (Et+ It + Dt) =	0.4 * Al (El + Il + Dl) =
**PASI totals**				
PASI = 0.1 * Ah (Eh + Ih + Dh) + 0.2 * Au (Eu + Iu + Du) + 0.3 * At (Et+ It + Dt) + 0.4 * Al (El + Il + Dl)				
Signature of scorer: ____________________			Date:__________________	

After washout period assessment

Psoriasis Area and Severity Index, PASI				
	**Head (h)**	**Upper limbs (u)**	**Trunk (t)**	**Lower limbs (l)**
**Erythema (E)**	☐0<br>☐3<br>☐1<br>☐4<br>☐2	☐0<br>☐3<br>☐1<br>☐4<br>☐2	☐0<br>☐3<br>☐1<br>☐4<br>☐2	☐0<br>☐3<br>☐1<br>☐4<br>☐2
**Thickness (D)**	☐0<br>☐3<br>☐1<br>☐4<br>☐2	☐0<br>☐3<br>☐1<br>☐4<br>☐2	☐0<br>☐3<br>☐1<br>☐4<br>☐2	☐0<br>☐3<br>☐1<br>☐4<br>☐2
**Scaling (I)**	☐0<br>☐3<br>☐1<br>☐4<br>☐2	☐0<br>☐3<br>☐1<br>☐4<br>☐2	☐0<br>☐3<br>☐1<br>☐4<br>☐2	☐0<br>☐3<br>☐1<br>☐4<br>☐2
**Lesion Score Sum (A)**	☐0 = 0%<br>☐1 = 1–9%<br>☐2 = 10–29%<br>☐3 = 30–49%<br>☐4 = 50–69%<br>☐5 = 70–89%<br>☐6 = 90–100%	☐0 = 0%<br>☐1 = 1–9%<br>☐2 = 10–29%<br>☐3 = 30–49%<br>☐4 = 50–69%<br>☐5 = 70–89%<br>☐6 = 90–100%	☐0 = 0%<br>☐1 = 1–9%<br>☐2 = 10–29%<br>☐3 = 30–49%<br>☐4 = 50–69%<br>☐5 = 70–89%<br>☐6 = 90–100%	☐0 = 0%<br>☐1 = 1–9%<br>☐2 = 10–29%<br>☐3 = 30–49%<br>☐4 = 50–69%<br>☐5 = 70–89%<br>☐6 = 90–100%
**Palm areas**<br>**(one palm ≈ 1% of BSA)**	one palm ≈ 11.1% of head and neck area	one palm ≈ 5.6% of the upper extremities	1 palm ≈ 3.7% of the trunk	1 palm ≈ 2.2% of lower extremities
	______Palms	______Palms	______Palms	______Palms
**BSA**	% (palms of h + palms of u + palms of t + palms of l)			
**Subtotals**	0.1 * Ah (Eh + Ih + Dh) =	0.2 * Au (Eu + Iu + Du) =	0.3 * At (Et+ It + Dt) =	0.4 * Al (El + Il + Dl) =
**PASI totals**				
PASI = 0.1 * Ah (Eh + Ih + Dh) + 0.2 * Au (Eu + Iu + Du) + 0.3 * At (Et+ It + Dt) + 0.4 * Al (El + Il + Dl)				

Static Physician Global Assessment, sPGA	
☐0 Clear	No signs of psoriasis (post inflammatory hyperpigmentation may be present)
☐1 Almost clear	Intermediate between mild and clear
☐2 Mild	Slight plaque elevation, scaling, and/or erythema
☐3 Mild to moderate	Intermediate between moderate and mild
☐4 Moderate	Moderate plaque elevation, scaling, and/or erythema
☐5 Moderate to Severe	Marked plaque elevation, scaling, and/or erythema
☐6 Severe	Very marked plaque elevation, scaling, and/or erythema

Signature of scorer: ____________________ Date:___________________

Selection Criteria

Inclusion criteria

	Yes	No
Clinically diagnosed as psoriasis vulgaris	☐	☐
PASI scores ＜ 10 or BSA ＜ 10%	☐	☐
Aged between 18 and 65	☐	☐
Written informed consent is provided	☐	☐

Exclusion criteria

	Yes	No
Currently are pregnant and lactating patients	☐	☐
Currently have uncontrolled or severe diseases, such as cardiovascular, respiratory, digestive, urinary, haematological or psychiatric diseases; have any known malignancy or a history of malignancy	☐	☐
Are allergic to the medications used in this study	☐	☐
Currently are participating in or have participated in other clinical trial(s) in the previous month	☐	☐
Not complete a wash-out period of other psoriasis therapies as listed below:	☐	☐

Therapies	Washout requirements
Topical agents: Glucocorticoids, Calcineurin inhibitors (e.g. Tacrolimus, Pimecrolimus), Vitamin D analogues (e.g. Calcipotriol, Tacalcitol), Retinoids (e.g. tazarotene), combination treatments (eg. Compound Clobetasol Propionate, Carpotriol betamethasone), Keratin promoter (e.g. 2% – 5% coal tar, 3% salicylic acid, 0.1% – 0.5% anthralin, 5% ichthammol), Keratolytic (e.g. 5% – 10% salicylic acid, 0.1% Retinoids), anthralin, and so on	two weeks
Antimicrobials; Systemic nonbiologic therapies: methotrexate, cyclosporine, acitretin, azathioprine, leflunomide, Mycophenolate Mofetil and so on; Phototherapies: NB-UVB, PUVA, 308 Excimer laser and so on	four weeks
Biologics and their half-lives approved by China now: Etanercept 3.5 days, Infliximab 10 days, Adalimumab 14 days, Ustekinumab 21 days, Guselkumab 18 days, Secukinumab 27 days, Ixekizumab 13 days	Five times of the half-life period of biologics

Whether the patient is enrolled

☐Yes, Date:__________________

Serial number of enrolments:__________

Randomisation code:____________

☐No, Reason: ☐PASI or BSA is not appropriate

☐Other:____________

Signature of the researcher: ____________ Date:________________
