# Supplementary material for: Add-on effects of Chinese herbal medicine external application (FZHFZY) to topical urea for mild-to-moderate psoriasis vulgaris: Protocol for a double-blinded randomized controlled pilot trial embedded with a qualitative study
Source: PLoS One. 2024 Mar 21;19(3):e0297834. doi: 10.1371/journal.pone.0297834 (PMC10956750; doi:10.1371/journal.pone.0297834)
Supplement: S4 File — (DOCX) [file pone.0297834.s005.docx]

**S4 File. Informed consent of the pilot RCT**

**Dear sir/ Madam,**

You are diagnosed with psoriasis vulgaris.

You are invited to take part in this research project entitled “Add-on Chinese herbal medicine (CHM) external application (FZHFZY) for mild-to-moderate psoriasis vulgaris: a pilot randomised placebo-controlled trial”. The aim of the pilot trial is to assess the feasibility of the study protocol and preliminary estimate the add-on effect and safety of CHM external application FZHFZY to topical urea cream for patients with mild-to-moderate psoriasis vulgaris.

This informed consent form provides details of the research project. Knowing what is involved will help you decide if you want to participate in the research. Please read this information carefully. If there is anything you would like to know more about, please feel free to ask questions. Before making any decision, you may consult with your medical doctor or others.

**Introduction**

1. **Background**

Current conventional therapies for the management of psoriasis vulgaris are often associated with insufficient long-term symptomatic relief, high cost and unwanted side effects. There has been certain evidence from clinical studies and systematic reviews supporting the use of CHM external application for the treatment of psoriasis vulgaris, however, a lack of standardised clinical trials and unknown herbal constituents in CHM formulas make it difficult to interpret and replicate the results. The CHM formula *Fu zheng he fu zhi yang* (FZHFZY) developed by Prof. Chuanjian Lu, an experienced clinician of Chinese medicine in dermatology, has been used as an external application for over decades to manage psoriasis symptoms. The safety and potential effects of this formula has been proved by pre-clinical studies and clinical observational studies prior to this randomised controlled trial.

This will be a two-arm, parallel, randomised, placebo-controlled, double-blind, pilot trial. It aims to assess the feasibility of the study setting, eligible criteria, intervention, outcome measurements, participant timeline and recruitment strategies, as well as obtain initial estimates of the add-on efficacy and safety in the treatment group. Results from the pilot study will help us to optimise the study design.

The research is a part of Dr. Junyue Wang’s PhD project. The project is supervised by Prof. Charlie Changli Xue, Prof. Chuanjian Lu, Prof. Anthony Lin Zhang and Dr. Claire Shuiqing Zhang.

This research has been initiated by the researcher Prof. Charlie Changli Xue and Prof. Chuanjian Lu.

This research is funded by the Department of Science and Technology of Guangdong Province. The PhD scholarship is provided by the School of Health and Biomedical Sciences, RMIT University.

This trial will be conducted in the dermatology outpatient clinic at the Guangdong Provincial Hospital of Chinese Medicine (GPHCM). It will recruit 60 eligible participants.

The project has been approved by the Ethics Committee of the GPHCM (No. BF2022-189-01) and registered with the RMIT University Human Ethics Advisory Network (No. 2022-25746-18453).

1. **The eligible criteria of the study.**

You may participate in the clinical trial if you are diagnosed with mild to moderate psoriasis vulgaris, your age is between 18 and 65 years, and the signed informed consent form is provided.

However, if you meet any of the exclusion criteria, you will not be included because it may cause potential risks: 1) currently are pregnant and lactating, 2) have uncontrolled or severe diseases, 3) are allergic to the medications used in this study; 4) currently are participating in or have participated in other clinical trial(s) in the previous month; 5) are not able or unwilling to stop using other treatments for psoriasis during the trial.

We will assess your condition and let you know if you are eligible for the trial.

1. **If I agree to participate, what will I be required to do?**
   1. You will complete these items before you are involved in the clinical trial. (First visit — screening: 30 minutes)

If you agree to participate in the trial, the researcher will assess your eligibility of the trial according to your medical history, the severity of psoriasis and concomitant medications. In addition, you will be required to cease other treatments for your psoriasis including topical medications, systemic medications, phototherapies and biologics.

After completing the wash-out period, you will undertake some chemical examinations, including blood count, urinalysis, hepatic and renal functions and electrocardiograms, to assess the eligibility and understand your condition before treatments. For these chemical examinations, you will provide your blood (two 4 ml-EDTA tubes and two drying tubes) and urine (one 10 ml-urine tube).

- 1. If you are eligible for the study, you will be required to do the following.

Before receiving the treatments, you will be randomly assigned to the intervention group or the control group. The intervention group is CHM granules plus 10% urea cream. The control group is placebo granules plus 10% urea cream. You will also be provided with cetirizine hydrochloride tablets as an additional management for unbearable itch caused by your psoriasis. Each participant has the same chance to be assigned to one of above-mentioned groups. Both of you and the researcher will not know which group you will be assigned to. This is a 20-week trial period, including an eight-week treatment phase and a 12-week follow-up phase.

For the CHM bath therapy, please dissolve one package of the granule in warm water (at 35–38 °C) with 60 litres in a container. You may adjust the amount of water according to the size of your bathtub and the location of your skin lesion with a fixed concentration. Please note that the height of liquid should not exceed your chest when you are in a semi-recumbent or sitting position if a whole-body bath is required. The bathing duration is between 15 and 20 minutes once a day for eight weeks at home. The water temperature will be checked by a water thermometer, and the bath duration of each session will be set an alarm to follow it. In addition, you will apply 10% urea cream topically on psoriasis lesions twice a day following the fingertip unit (FTU) method. One FTU is measured as the amount (about 0.5 grams) of medication that covers from the tip of your finger to the first crease and the amount is sufficient to cover both sides.

During the trial, you will be required to visit us for re-examination and data collection, as follows:

Second visit — Baseline (30 minutes to 1 hour): You will fill in a few questionnaires related to psoriasis symptoms and quality of life. The researcher will record your condition, assess the severity of psoriasis, and photographs the representative skin lesions. These photos will not reveal your facial identity and private parts, and then will be coded so they are not identifiable. We will collect your biological specimens for future research on psoriasis disease. It will collect blood (two 5 ml-EDTA tubes, two 5 ml-procoagulant tubes, and one 2.5 ml-BD tube), urine (two 10 ml-urine tubes), skin microbiota (four microtubes), stool (two stool tubes) and oral cavity microbiota (two microtubes). If you reject to provide these samples, please notify the research team. You can still participate in the trial and it will not affect your involvement.

From the third to fifth visit — Week 2, 4, 6 (15 minutes): You will fill in a few questionnaires related to psoriasis symptoms. The researcher will record your condition, assess the severity of psoriasis, and photographs the representative skin lesions.

Sixth visit — Week 8 (30 minutes to 1 hour): You have completed finish the treatment phase. You will fill in a few questionnaires related to psoriasis symptoms and quality of life. The researcher will record your condition, evaluate the severity of psoriasis, and photographs the representative skin lesions. In addition, you will undertake some chemical examinations, including blood count, urinalysis, hepatic and renal functions, to assess your condition after treatments. For these chemical examinations, you will provide your blood (two 4 ml-EDTA tubes and two drying tubes) and urine (one 10 ml-urine tube). We will also collect some biological specimens for future research on psoriasis disease. It will collect blood (two 5 ml-EDTA tubes and two 5 ml-procoagulant tubes), urine (two 10 ml-urine tubes) and skin microbiota (four microtubes). If you reject to provide these samples, please notify the research team. You can still participate in the trial and it will not affect your involvement.

From the seventh to eighth visit — Week 12, 16 (15 minutes): You will come to the hospital for assessments same as the third visit.

Ninth visit — Week 20 (30 minutes to 1 hour): You finish the follow-up phase. You will come to the hospital for examinations same as the sixth visit.

- 1. Other requirements

You will keep a home diary which contains a few questions about your psoriasis symptoms and concomitant medications. You will also provide any unused CHM or urea cream at each visit during the treatment phase.

You will be required to stop using other treatments for psoriasis during the trial. If you want to apply for other treatments for psoriasis, please contact the researcher in advance.

Suggestions on diet and daily life: keep light diet, avoid getting cold.

- 1. Reasons of termination in the study

If you are diagnosed with other type psoriasis (erythrodermic psoriasis, psoriatic arthritis, or pustular psoriasis) by the researchers in the trial, the study will be terminated.

If you suffer from serious adverse events during the trial, the study will be terminated.

1. **What are the potential benefits of taking part in this pilot RCT?**

The consultation fee for you to visit the dermatologist is waived during the trial phase. You will be assigned with a dermatologist who is included in the research team during the whole procedure of this trial.

You will have a 50% chance to receive the CHM treatment provided by this trial. You may find this therapy is convenient and beneficial for your psoriasis and then keep using it in the future. On the other hand, your participation in this trial will make contribution to the development of a promising and effective therapy for psoriasis to benefit other patients.

If you are in the placebo group, after the completion of the study, the research team will provide you with the real CHM for an eight-week usage for free.

You will receive a detailed and comprehensive assessment of psoriasis and examinations regularly, which will provide you with dynamic results related to the disease.

1. **What are the possible risks of taking part in this pilot RCT?**

Although the CHM bath therapy is a relatively safe treatment with a low risk of side effects, you may suffer from skin irritation (e.g. skin erythema, itching), chest tightness or shortness of breath during the bath. These symptoms may disappear if you stop the bath immediately. You can contact the researcher to seek advice. We will give your detailed suggestions.

If you suffer from any unexpected condition no matter whether it is related to the trial medications or not, please contact the researcher immediately, we will give your detailed suggestions.

It will be time consuming for you to visit the hospital multiple times. We will prioritise your appointment as a trial participant to reduce the waiting time.

You may feel uncomfortable when undertaking some chemical examinations, same as you are undergoing other medical examinations.

In addition, it is possible the trial medications do not provide sufficient treatment effects as you expected. Please feel free to discuss this with the researcher. The research team may cease the trial if we find the treatments are ineffective or cause other harms, then we will provide the best available treatment plan to you according to your condition.

1. **What is the cost?**

The researcher team will pay for your consultation fee, chemical examinations fee, and trial medications fee during the trial, including CHM/ placebo granules, 10% urea cream, and cetirizine hydrochloride tablets. You will be reimbursed for A$60 to cover the cost of travelling to the hospital.

If you suffer from serious adverse effects caused by the trial medications, we will pay for medical costs and provide financial recompense according to laws.

The cost of other diseases will not be covered by the trial.

1. **What will happen to the information I provide?**

Research data will be kept securely at a password-protected computer, and case report forms will be locked in a cabinet of GPHCM for a minimum of 15 years after publication before being destroyed. Only study investigators will have access to your information. If you suffer from serious adverse events, the Ethics Committee of the GPHCM will check your research data. If the medical record in the trial is used in other studies, we will get ethics approval. Any information obtained from the study that can identify you will remain confidential; we will protect your privacy unless required by law.

The results of the research will be included in the PhD thesis of Dr. Junyue Wang. Once approved, it will be submitted to the RMIT University Research Repository. The finding from the trial will be published in international peer-reviewed journals and will be presented at conferences. You will not be identified in any publications from the study. Published research data is displayed as group data rather than data related to specific individuals. If you are interested in the results, we can provide you with published journal articles.

1. **Who should I contact if I have any questions?**

Any questions relating to the study can be directed to Dr. Junyue Wang at XXX and XXX.

Any complaints relating to the involvement of the study, please contact the Ethics committee of the GPHCM (Tel. XXX).

If there is any factor that influences your willingness to take part in the study, the researcher will contact you immediately.

1. **Can I choose to participate in or withdraw from the study?**

You have the right to withdraw from the study at any time for any reason, which will not impact your relationship with the researcher and the quality of medical care provided by the hospital.

Your researcher may terminate your participation in this study at any time considering your best interest.

If you do not participate in the study or withdraw from the study, there are many alternative treatments, including CHM and conventional therapies.

If you withdraw from the study for any reason, you may be required to provide feedback about the use of trial medications and to complete relevant examinations, which will benefit the care of your health.

1. **What happens to** **the biological specimens after the study?**

If you agree to provide your biological specimens, we will collect your blood, urine and faeces, skin flora and oral flora during the trial. All collected specimens will be de-identified to protect your privacy unless required by law. These specimens will be stored in the GPHCM for 10 years after the end of the study and will be disposed after the preservation period. You have the right to request to dispose the unused biological specimens during the preservation period. These specimens may be used to explore the mechanism of psoriasis in the future and tested in different laboratories as needed. In these cases, we will re-submit the ethics application to the Ethics Committee of GPHCM. These specimens can be used after getting the ethics approval.

1. **What should I do now?**

Please read this information carefully. If there is anything you would like to know more about, please feel free to ask questions.

Before making any decision, you may consult with your medical doctor or others.

Thank you for reading the above information. If you decide to participate in the trial, please contact Dr. Junyue Wang who will make all the study arrangements for you.

You will be given a copy of this informed consent form to keep.

**Signature page for informed consent**

**Project title:** Add-on effects of Chinese herbal medicine (CHM) external application (FZHFZY) for mild-to-moderate psoriasis vulgaris: a pilot randomised placebo-controlled trial

**Sponsor:** Department of Science and Technology of Guangdong Province

**No. Ethics Approval:** BF 2022-189-01

**Consent**

I have read the above information and have had an opportunity to ask questions and discuss with the researcher. All my questions have been thoroughly answered.

I understand the potential benefits and risks of the project. I freely agree to participate in the study and ensure I have enough time to think about it. I understand that:

- I can contact the researcher for more information at any time.
- I am free to withdraw from the project at any time. I will not be subject to discrimination or revenge if I do not participate in or withdraw from the study, and the quality of medical care provided by the hospital will not be affected.

I agree that if I withdraw from the study, I will provide my feedback on the medications, complete the relevant examinations, which will help the researcher to collect data and benefit the care of my health.

If I take other treatments for psoriasis, I will ask the researcher in advance or tell the researcher truth after using.

I agree that the staff from the Therapeutic Goods Administration and the Ethics committee will have access to my research data.

I understand that I will be given a signed copy of this informed consent form to keep.

Finally, I agree with participating in the clinical trial.

Signature of participant: Date: Tel.:

For the biological specimens, I 🞎agree/🞎disagree with providing specimens of blood, urine and faeces, skin flora and oral flora for future research and storing them in the GPHCM for 10 years after the end of the study.

Signature of participant: Date:

I acknowledge that I have provided details of the project, including the potential benefits and risks. I will give a signed copy of this informed consent form to the participant.

Signature of researcher: Date: Tel.:

Telephone number of the Ethics committee of the GPHCM: XXX.
