# Supplementary material for: Add-on effects of Chinese herbal medicine external application (FZHFZY) to topical urea for mild-to-moderate psoriasis vulgaris: Protocol for a double-blinded randomized controlled pilot trial embedded with a qualitative study
Source: PLoS One. 2024 Mar 21;19(3):e0297834. doi: 10.1371/journal.pone.0297834 (PMC10956750; doi:10.1371/journal.pone.0297834)
Supplement: S5 File — (DOCX) [file pone.0297834.s006.docx]

**S5 File. Quantitative analysis of active compounds and the HPLC fingerprint of FZHFZY granules**

# **Part 1. Chemical analysis and HPLC fingerprint of FZHFZY granules**

## **Method**

The chemical composition of FZHFZY granules was analysed by high-performance liquid chromatography (HPLC).

## **Chromatographic conditions and system suitability test chromatographic conditions**

The column was Waters Xbrige BEH C18 (100 mm × 2.1 mm, 1.7 μm). The mobile phase consisted of solvent A (acetonitrile) and solvent B (0.1% glacial acetic acid solution) with gradient elution (Table 1). The flow rate was 0.3 mL·min^–1^. The column temperature was 30 ℃. The UV detection wavelength was set at 291 nm.

**Table 1. Details of gradient elution**

| Duration (minutes) | Solvent A (%) | Solvent B (%) |
| --- | --- | --- |
| 0 ~ 3 | 3 | 97 |
| 3 ~ 8 | 3 → 15 | 97 → 85 |
| 8 ~ 18 | 15 → 45 | 85 → 55 |
| 18 ~ 21 | 45 → 80 | 55 → 20 |
| 21 ~ 22 | 80 → 3 | 20 → 97 |

## **The preparation of the FZHFZY solution**

The sample of FZHFZY granules was accurately weighed at 0.5 g and put into a conical flask with a stopper. Twenty-five mL of methanol/water (80/20, v/v) was added into the container and then weighed again. The sample was ultrasonicated for 30 minutes with a 250 W power and 40 kHz frequency. When the sample cooled down, the methanol/water (80/20, v/v) would be added to supply the decreased weight. The sample solution could be obtained after shaking and filtration.

## **The preparation of the control** **solution**

Astilbin sample was accurately weighed and added into methanol/water (80/20, v/v) to make a control solution with 15 µg astilbin per millilitre. The control solution could be obtained after shaking.

## **Determination**

One μL FZHFZY solution and 1 μL control solution were injected into HPLC respectively for determination.

## **Results**

Nine characteristic peaks were identified in the HPLC fingerprint of the FZHFZY solution and peak S represented the control solution under the developed HPLC method. (Fig. 1). Peaks 1 – 9 are corresponding to adenosine, ferulic acid, neoastilbin, astilbin, isoastilbin, neoisoastilbin, isomaculosidine, paeonol and osthole. Chemical structures of identified compounds in FZHFZY are presented, corresponding to the peak numbers indicated in the chromatogram in Fig. 2. The relative retention duration of each characteristic peak compared to the peak was calculated, and the relative retention duration should be within ± 15% of the specified value. The specified value about the relative retention duration of each characteristic peak is 0.21 (peak 1), 0.94 (peak 2), 0.98 (peak 3), 1.00 (peak 4), 1.04 (peak 5), 1.05 (peak 6), 1.34 (peak 7), 1.38 (peak 8), 1.75 (peak 9).


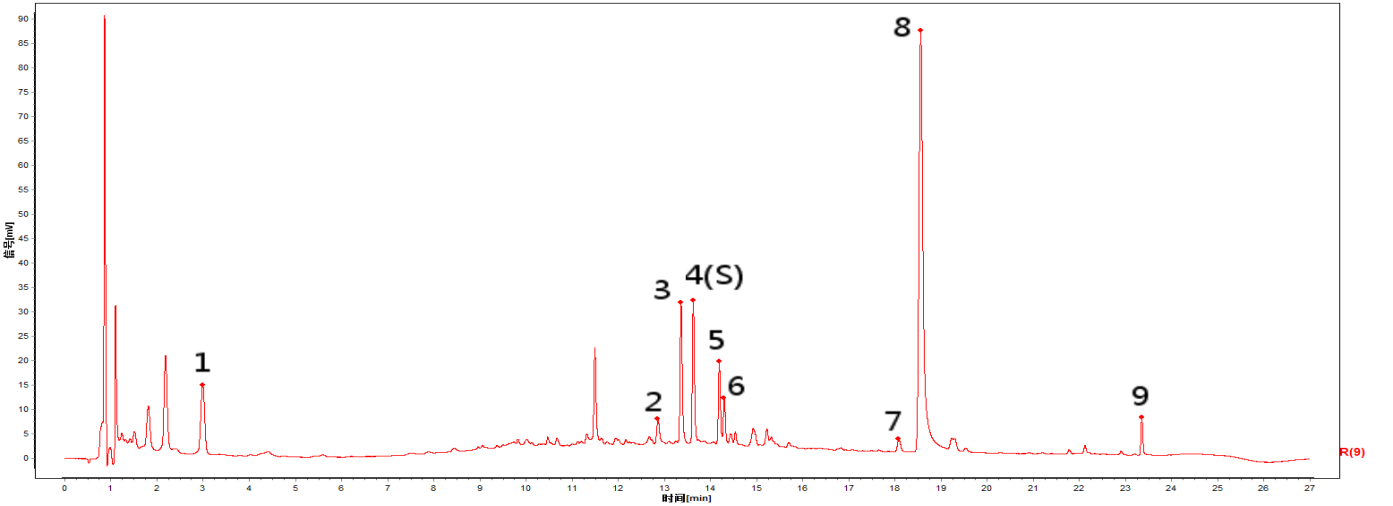


**Fig. 1. HPLC fingerprint of FZHFZY granules.** This figure shows the HPLC fingerprints of FZHFZY and the control solution. Nine characteristic peaks were identified in the HPLC fingerprint of the FZHFZY solution and peak S represented the control solution under the developed HPLC method. Peak 1 is adenosine; Peak 2 is ferulic acid; Peak 3 is neoastilbin; Peak 4 (S) is astilbin; Peak 5 is isoastilbin; Peak 6 is neoisoastilbin; Peak 7 is isomaculosidine; Peak 8 is paeonol; Peak 9 is osthole. The minimum peak area of the integral parameter of the HPLC fingerprint of the control solution is 0.1.


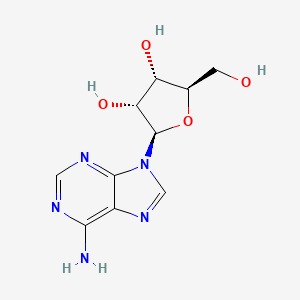

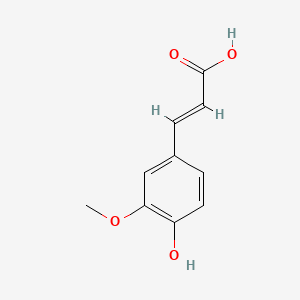

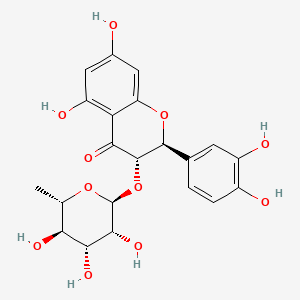


1. Adenosine 2. Ferulic acid 3. Neoastilbin


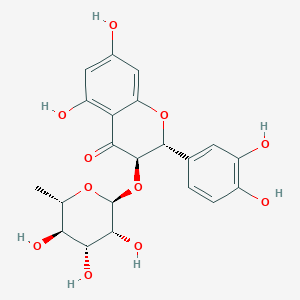

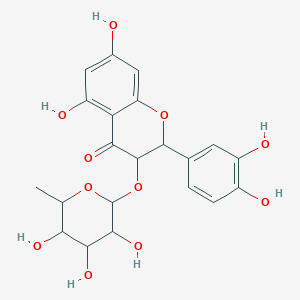

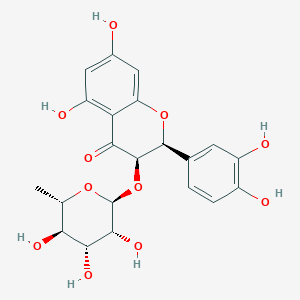


4. Astilbin 5. Isoastilbin 6. Neoisoastilbin


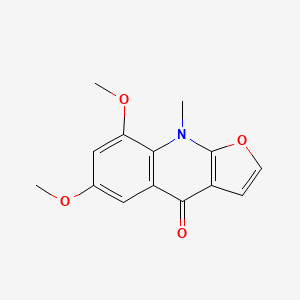

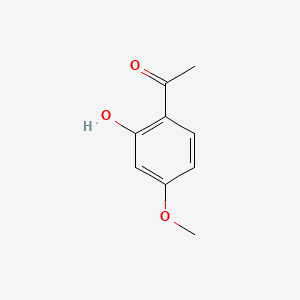

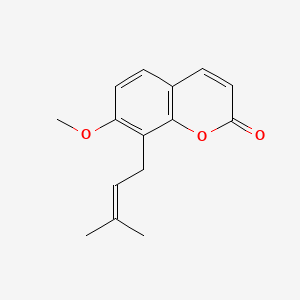


7. Isomaculosidine 8. Paeonol 9. Osthole

**Fig. 2. Chemical structures of identified compounds in the HPLC fingerprint of FZHFZY.** This figure shows the chemical structures of identified compounds in FZHFZY, corresponding to the peak numbers indicated in the HPLC fingerprint. The chemical structure depiction is cited from the National Centre for Biotechnology Information (<https://pubchem.ncbi.nlm.nih.gov/>, assessed 29 June 2023).

# **Part 2. Quantitative analysis of active compounds** **in FZHFZY granules**

Paeonol and astilbin, active compounds of FZHFZY granules, have been proven to be effective for the management of psoriasis. Paeonol can regulate the expression of gene autophagy-related 5 to manage psoriasis in vitro (Zhang, 2021). Paeonol also inhibited the maturation and activation of dendritic cells to ameliorate imiquimod-induced psoriasis-like skin lesions (Meng, 2017). Topical astilbin inhibited the dendritic cell-Th17 inflammation axis and then ameliorated imiquimod-induced psoriasis-like skin lesions in SKH-1 mice (Xu, 2022). In addition, the astilbin can reduce reactive oxygen species (ROS) accumulation and vascular endothelial growth factor (VEGF) expression through Nrf2 in psoriasis-like skin disease (Wang, 2019). Therefore, accurate active compound concentrations of paeonol and astilbin in FZHFZY granules will be determined.

## **Quantitative analysis of paeonol in FZHFZY granules**

### **Method**

The accurate active compound concentration of paeonol in FZHFZY granules was determined by HPLC.

### **Chromatographic conditions and system suitability test chromatographic conditions**

The column was Waters Xbrige BEH C18. The mobile phase consisted of methanol/water (45/55, v/v). The UV detection wavelength was set at 274 nm. The number of theoretical plates should not be less than 4,000 based on the calculation of the paeonol peak.

### **The preparation of the FZHFZY solution**

The sample of FZHFZY granules was accurately weighed at 0.5 g and put into a conical flask with a stopper. Twenty-five mL of methanol/water (80/20, v/v) was added into the container and then weighed again. The sample was ultrasonicated for 30 minutes with a 250 W power and 40 kHz frequency. When the sample cooled down, the methanol/water (80/20, v/v) would be added to supply the decreased weight. The sample solution could be obtained after shaking and filtration.

### **The preparation of the control solution**

The paeonol sample was accurately weighed and added into methanol/water (80/20, v/v) to make a control solution with 70 µg paeonol per millilitre. The control solution could be obtained after shaking.

### **Determination**

One μL FZHFZY solution and 1 μL control solution were injected into HPLC respectively for determination.

### **Results**

The HPLC fingerprint of paeonol was present in Fig 3. The accurate active compound concentration of paeonol in FZHFZY granules was not less than 1.56 mg/g.

A

B

C

**Fig. 3. The HPLC fingerprint of paeonol (A), FZHFZY deficient *Cynanchum paniculatum* (*Bge*.) Kitag. (*xu chang qing*) (B) and FZHFZY granules sample (C).**

## **Quantitative analysis of astilbin in FZHFZY granules**

### **Method**

The accurate active compound concentration of astilbin in FZHFZY granules was determined by HPLC.

### **Chromatographic conditions and system suitability test chromatographic conditions**

The column was Waters Xbrige BEH C18. The mobile phase consisted of solvent A (acetonitrile) and solvent B (0.1% glacial acetic acid solution) with gradient elution (Table 2). The flow rate was 0.3 mL·min^–1^. The UV detection wavelength was set at 291 nm. The number of theoretical plates should not be less than 200,000 based on the calculation of the astilbin peak.

**Table 2. Details of gradient elution**

| Duration (minutes) | Solvent A (%) | Solvent B (%) |
| --- | --- | --- |
| 0 ~ 3 | 3 | 97 |
| 3 ~ 8 | 3 → 15 | 97 → 85 |
| 8 ~ 18 | 15 → 45 | 85 → 55 |
| 18 ~ 21 | 45 → 80 | 55 → 20 |
| 21 ~ 22 | 80 → 3 | 20 → 97 |

### **The preparation of the FZHFZY solution**

The sample of FZHFZY granules was accurately weighed at 0.5 g and put into a conical flask with a stopper. Twenty-five mL of methanol/water (80/20, v/v) was added into the container and then weighed again. The sample was ultrasonicated for 30 minutes with a 250 W power and 40 kHz frequency. When the sample cooled down, the methanol/water (80/20, v/v) would be added to supply the decreased weight. The sample solution could be obtained after shaking and filtration.

### **The preparation of the control solution**

Astilbin sample was accurately weighed and added into methanol/water (80/20, v/v) to make a control solution with 15 µg astilbin per millilitre. The control solution could be obtained after shaking.

### **Determination**

One μL FZHFZY solution and 1 μL control solution were injected into HPLC respectively for determination.

### **Results**

The HPLC fingerprint of astilbin was present in Fig 4. The accurate active compound concentration of astilbin in FZHFZY granules was not less than 0.27 mg/g.

A

B

C

**Fig. 4. The HPLC fingerprint of astilbin (A), FZHFZY deficient *Smilax glabra* Roxb. (*tu fu ling*) (B) and FZHFZY granules sample (C)**

# **References:**

Meng Y, Wang M, Xie X, et al. Paeonol ameliorates imiquimod-induced psoriasis-like skin lesions in BALB/c mice by inhibiting the maturation and activation of dendritic cells. Int J Mol Med. 2017;39(5):1101-1110. doi:10.3892/ijmm.2017.2930.

Wang W, Yuhai, Wang H, Chasuna, Bagenna. Astilbin reduces ROS accumulation and VEGF expression through Nrf2 in psoriasis-like skin disease. Biol Res. 2019;52(1):49. Published 2019 Sep 6. doi:10.1186/s40659-019-0255-2.

Xu Q, Liu Z, Cao Z, et al. Topical astilbin ameliorates imiquimod-induced psoriasis-like skin lesions in SKH-1 mice via suppression dendritic cell-Th17 inflammation axis. J Cell Mol Med. 2022;26(4):1281-1292. doi:10.1111/jcmm.17184.

Zhang Q, Shi H, Zhang J, Jiang C, Zhou C. The paeonol target gene autophagy-related 5 has a potential therapeutic value in psoriasis treatment. PeerJ. 2021;9:e11278. Published 2021 May 25. doi:10.7717/peerj.11278.
