# Supplementary material for: Add-on effects of Chinese herbal medicine external application (FZHFZY) to topical urea for mild-to-moderate psoriasis vulgaris: Protocol for a double-blinded randomized controlled pilot trial embedded with a qualitative study
Source: PLoS One. 2024 Mar 21;19(3):e0297834. doi: 10.1371/journal.pone.0297834 (PMC10956750; doi:10.1371/journal.pone.0297834)
Supplement: S6 File — (PDF) [file pone.0297834.s007.pdf]

Add-on Chinese herbal medicine (CHM) external application (FZHFZY) for mild-to-moderate psoriasis vulgaris: a pilot randomised placebo-controlled trial

Patient Home Diary (the treatment period)

Serial number of the enrolment : |__|__|__|

Randomisation code : |__|__|__|

Abbreviation of the patient’s name : |__|__|__|__|

The researcher : ________________________

Guangdong Provincial Hospital of Chinese Medicine

This page is filled in by the researcher.

Patient home diary completing guidelines:

1. Please record the home diary from the date of using trial medications.
2. Please record any symptoms during the treatment period. If there are severe conditions, please contact the researcher immediately.
3. The home diary of the patient with psoriasis vulgairs is kept by the patients. **Please bring it back to the hospital during each visit.**
4. Please apply these instructions in your daily life during the trial:
   - Have a good rest, avoid cold, avoid suffering from inflammatory diseases such as acute pharyngitis and tonsillitis
   - Avoid alcohol, ensure enough sleep, and maintain good mood
   - Keep the skin clean and avoid bathing in hot water.

Administration instructions of trial medications:

1. Please dissolve one package of the granule in warm water (at 35–38 °C) with 60 litres in a container. You may adjust the amount of water according to the size of your bathtub and the location of your skin lesion with a fixed concentration. Please note that the height of liquid should not exceed your chest when you are in a semi-recumbent or sitting position if a whole-body bath is required. The bathing duration is between 15 and 20 minutes once a day for eight weeks at home. The water temperature can be checked by a water thermometer, and the bath duration of each session can be set an alarm to follow it.
2. You will apply 10% urea cream topically on psoriasis lesions twice a day following the fingertip unit (FTU) method. One FTU is measured as the amount (about 0.5 grams) of medication that covers from the tip of your finger to the first crease and the amount is sufficient to cover both sides.
3. You may be provided with cetirizine hydrochloride tablets for managing unbearable itch caused by psoriasis by the research team. Take one tablet (10 mg) at a time at night. Please document the time of dosage in the diary if you have taken this medication.

Administration precautions of trial medications:

1. The external application as whole-body bath or topical bath, as well as the amount of water and dosage will depend on the size of your bathtub and the location of skin lesion, as well as the acceptability of patients.
2. Take a bath after cleansing the skin. The right liquid temperature is between 35°C to 38°C according to individual preference.
3. The height of liquid should not exceed your chest when you are in a semi-recumbent or sitting position if a whole-body bath is required.
4. Pay attention to ventilation when bathing the whole body, it is not suitable for closed space.
5. Avoid getting cold and drinking alcohol, prevent fall injury before and after bathing.
6. Please suspend the bath with cold and fever.
7. Please suspend the bath for women during menstruation.
8. If you have observed any allergic reaction, please stop the bath therapy and contact the researcher immediately for further instruction.
9. If there is any skin damage caused by injuries, please stop the bath therapy and contact the researcher immediately for further instruction.
10. If you feel unwell during the bath, e.g. skin irritation, chest tightness and shortness of breath, please discontinue it.

Abbreviation of the patient's name |__|__|__|__| Serial number of the enrolment |__|__|__|

The treatment period

Baseline time:_________________   Start using trial medications: _________________

	Week 1							Week 2						
Date/month														
Used CHM bath?														
Used topical urea cream?														
Did you have any other symptoms?														
If you had other symptoms, did you take any treatment? Please specify.														
Psoriasis symptoms aggravation or improvement?	(record date and details):													
Psoriasis symptoms aggravation or improvement?	(potential factors):													

- If the date is "1st February", please indicate "1/2" in the table. Please record medication information for two weeks in the form. If you cannot return to the hospital on the date as the appointment, you can return to the hospital within 3 days before and after the appointment.
- If you have used CHM bath and urea cream on the same day, please put a "√" in the corresponding box; if not, put a "×" in the corresponding box.
- If you had any other symptoms than psoriasis, please put a "√" in the corresponding box, and record your treatment if there is any. Otherwise put a "×" in the box.
- If other diseases require long-term treatment, please fill in the "Concomitant medication form" on the back.
- If your psoriasis symptoms worsened/improved, record the date of occurrence, details and possible influencing factors in the corresponding box of the table.

Abbreviation of the patient's name |__|__|__|__| Serial number of the enrolment |__|__|__|

Concomitant medication form

Trade name or generic name	Usage daily	Disease	Start date	End date	Ongoing

- If the medication is still in use at the visit, the "end date" box is not required to be filled in, please put a " √ " in the "ongoing" box. If the medication has been discontinued at the visit, please provide the "end date" and put a "×" in the "ongoing" box.

Abbreviation of the patient's name |__|__|__|__| Serial number of the enrolment |__|__|__|

The treatment period

	Week 3							Week 4						
Date/month														
Used CHM bath?														
Used topical urea cream?														
Did you have any other symptoms?														
If you had other symptoms, did you take any treatment? Please specify.														
Psoriasis symptoms aggravation or improvement?	(record date and details):													
Psoriasis symptoms aggravation or improvement?	(potential factors):													

- If the date is "1st February", please indicate "1/2" in the table. Please record medication information for two weeks in the form. If you cannot return to the hospital on the date as the appointment, you can return to the hospital within 3 days before and after the appointment.
- If you have used CHM bath and urea cream on the same day, please put a "√" in the corresponding box; if not, put a "×" in the corresponding box.
- If you had any other symptoms than psoriasis, please put a "√" in the corresponding box, and record your treatment if there is any. Otherwise put a "×" in the box.
- If other diseases require long-term treatment, please fill in the "Concomitant medication form" on the back.
- If your psoriasis symptoms worsened/improved, record the date of occurrence, details and possible influencing factors in the corresponding box of the table.

Abbreviation of the patient's name |__|__|__|__| Serial number of the enrolment |__|__|__|

Concomitant medication form

Trade name or generic name	Usage daily	Disease	Start date	End date	Ongoing

- If the medication is still in use at the visit, the "end date" box is not required to be filled in, please put a " √ " in the "ongoing" box. If the medication has been discontinued at the visit, please provide the "end date" and put a "×" in the "ongoing" box.

Abbreviation of the patient’s name |__|__|__|__| Serial number of the enrolment |__|__|__|

The treatment period

	Week 5							Week 6						
Date/month														
Used CHM bath?														
Used topical urea cream?														
Did you have any other symptoms?														
If you had other symptoms, did you take any treatment? Please specify.														
Psoriasis symptoms aggravation or improvement?	(record date and details):													
Psoriasis symptoms aggravation or improvement?	(potential factors):													

- If the date is "1st February", please indicate "1/2" in the table. Please record medication information for two weeks in the form. If you cannot return to the hospital on the date as the appointment, you can return to the hospital within 3 days before and after the appointment.
- If you have used CHM bath and urea cream on the same day, please put a "√" in the corresponding box; if not, put a "×" in the corresponding box.
- If you had any other symptoms than psoriasis, please put a "√" in the corresponding box, and record your treatment if there is any. Otherwise put a "×" in the box.
- If other diseases require long-term treatment, please fill in the "Concomitant medication form" on the back.
- If your psoriasis symptoms worsened/improved, record the date of occurrence, details and possible influencing factors in the corresponding box of the table.

Abbreviation of the patient’s name |__|__|__|__| Serial number of the enrolment |__|__|__|

Concomitant medication form

Trade name or generic name	Usage daily	Disease	Start date	End date	Ongoing

- If the medication is still in use at the visit, the "end date" box is not required to be filled in, please put a " √ " in the "ongoing" box. If the medication has been discontinued at the visit, please provide the "end date" and put a "×" in the "ongoing" box.

Abbreviation of the patient's name |__|__|__|__| Serial number of the enrolment |__|__|__|

The treatment period

Week 7 **Week 8**

Date/month														
Used CHM bath?														
Used topical urea cream?														
Did you have any other symptoms?														
If you had other symptoms, did you take any treatment? Please specify.														
Psoriasis symptoms aggravation or improvement?	(record date and details):													
Psoriasis symptoms aggravation or improvement?	(potential factors):													

- If the date is "1st February", please indicate "1/2" in the table. Please record medication information for two weeks in the form. If you cannot return to the hospital on the date as the appointment, you can return to the hospital within 3 days before and after the appointment.
- If you have used CHM bath and urea cream on the same day, please put a "√" in the corresponding box; if not, put a "×" in the corresponding box.
- If you had any other symptoms than psoriasis, please put a "√" in the corresponding box, and record your treatment if there is any. Otherwise put a "×" in the box.
- If other diseases require long-term treatment, please fill in the "Concomitant medication form" on the back.
- If your psoriasis symptoms worsened/improved, record the date of occurrence, details and possible influencing factors in the corresponding box of the table.

Abbreviation of the patient's name |__|__|__|__|　　Serial number of the enrolment |__|__|__|

Concomitant medication form

Trade name or generic name	Usage daily	Disease	Start date	End date	Ongoing

- If the medication is still in use at the visit, the "end date" box is not required to be filled in, please put a " √ " in the "ongoing" box. If the medication has been discontinued at the visit, please provide the "end date" and put a "×" in the "ongoing" box.
