# Supplementary material for: Add-on effects of Chinese herbal medicine external application (FZHFZY) to topical urea for mild-to-moderate psoriasis vulgaris: Protocol for a double-blinded randomized controlled pilot trial embedded with a qualitative study
Source: PLoS One. 2024 Mar 21;19(3):e0297834. doi: 10.1371/journal.pone.0297834 (PMC10956750; doi:10.1371/journal.pone.0297834)
Supplement: S7 File — (PDF) [file pone.0297834.s008.pdf]

S7 File. Case report form

Add-on effects of Chinese herbal medicine (CHM) external application (FZHFZY) for mild-to-moderate psoriasis vulgaris: a pilot randomised placebo-controlled trial

Case Report Form

**Serial number of the enrolment**	:	|__|__|__|
**Randomisation code**	:	|__|__|__|
**Abbreviation of the patient's name**	:	|__|__|__|__|
**The researcher**	:	________________________

Guangdong Provincial Hospital of Chinese Medicine

Table of Contents

Case report form completing guidelines....1
Selection criteria....2
PASI calculation....3
Attached with the allocation code card here.....3
Record of photographing skin lesions....4
Baseline (Week 0)....5
Week 2....9
Week 4....11
Week 6....13
Week 8....15
Week 12....19
Week 16....21
Week 20....23
Summary of trial completion....27
Records of concomitant medication....28
Adverse event report form....29
Review statement of the case report form....31
Schedule for the trial....32

Please read the information carefully before filling in the case report form.

Case report form completing guidelines

1. Please fill in the case report form using preferably black or black-blue pen. Ensure all entries are accurate and legible to others. If any correction is needed, please draw a horizontal line to cross the original writing and then write the correct entry nearby, record the date and name of the correction. Please see the example given below:
   ~~58.6~~ 56.8, name of the modifier, 2022/10/12
2. Patients' names should be abbreviated with four Pinyin letters (capitalised). If the name consists of two Chinese characters, please use the first two Pinyin letters for each character. In the case of a Chinese character with only one Pinyin letter, please repeat it twice. If the name includes three characters, please use the first one letter for the first two characters and the first two letters for the third character. If the name includes four characters, please use the first one letter for each. Please see the example given below:

Zhang Hong	Z	H	H	O	Li E	L	I	E	E	Li Shu Ming	L	S	M	I	Si Ma Hong Xia	S	M	H	X

3. All "□" will be filled with "√". All "|__|" will be filled with digits. Please fill in the digits on the right if the digits are not enough for boxes and use "0" to fill in space boxes, such as "|_0_|_4_|". All boxes in the form cannot be left blank. If the chemical examinations are not conducted or missed, "ND" should be filled in. If the specific dosage and the time of taking medications are unknown, "UK" should be filled in. Enter "NA" for where information is "not available" or "not applicable".
4. Fill in the adverse events during the trial truthfully. Record the occurrence time, severity degree, duration, managements, and progression of adverse events. If there are severe adverse events, please notify the person immediately.

Chuanjian Lu	Tel: XXX

5. Please conduct the trial strictly according to the study protocol. The research activities and data collection in different phases should be conducted according to the schedule for the trial.

Selection criteria

Inclusion criteria

	Yes	No
Clinically diagnosed as psoriasis vulgaris	☐	☐
PASI scores ＜ 10 or BSA ＜ 10%	☐	☐
Aged between 18 and 65 years	☐	☐
Written informed consent is provided	☐	☐

If anyone of the above items is answered with 'No', the patient could not be recruited in the trial.

Exclusion criteria

	Yes	No
Currently are pregnant and lactating patients	☐	☐
Currently have uncontrolled or severe diseases, such as cardiovascular, respiratory, digestive, urinary, haematological or psychiatric diseases; have any known malignancy or a history of malignancy	☐	☐
Are allergic to the medications used in this study	☐	☐
Currently are participating in or have participated in other clinical trial(s) in the previous month	☐	☐
Not able or unwilling to wash out other psoriasis therapies as listed below:	☐	☐

Therapies	Washout requirements
Topical agents: Glucocorticoids, Calcineurin inhibitors (e.g. Tacrolimus, Pimecrolimus), Vitamin D analogues (e.g. Calcipotriol, Tacalcitol), Retinoids (e.g. tazarotene), combination treatments (eg. Compound Clobetasol Propionate, Carpotriol betamethasone), Keratin promoter (e.g. 2% – 5% coal tar, 3% salicylic acid, 0.1% – 0.5% anthralin, 5% ichthammol), Keratolytic (e.g. 5% – 10% salicylic acid, 0.1% Retinoids), anthralin, and so on	Two weeks
Antimicrobials; Systemic nonbiologic therapies: methotrexate, cyclosporine, acitretin, azathioprine, leflunomide, Mycophenolate Mofetil and so on; Phototherapies: NB-UVB, PUVA, 308 Excimer laser and so on	Four weeks
Biologics and their half-lives approved by China now: Etanercept 3.5 days, Infliximab 10 days, Adalimumab 14 days, Ustekinumab 21 days, Guselkumab 18 days, Secukinumab 27 days, Ixekizumab 13 days	Five times of the half-life period of biologics

If anyone of the above items is answered with 'Yes', the patient could not be recruited in the trial.

Informed consent

Date of signing informed consent:__________________

Attached with the allocation code card here.

PASI calculation

PASI at week 0		
PASI-50		PASI-50 = $PASI_{week\ 0} \times 50\%$
PASI-75		PASI-75 = $PASI_{week\ 0} \times (1 - 75\%)$
The lowest PASI of relapse		$PASI_{week\ 8} + 50\% \times (PASI_{week\ 0} - PASI_{week\ 8})$, for patients who achieve PASI-50 at week 8
The lowest PASI of rebound		$PASI_{week\ 0} \times 125\%$, for patients who achieve PASI-50 at week 8

Record of photographing skin lesions

Date	Visits (Week)	No. of photo	Signature of photographer	Representative skin lesions

Front **Back**

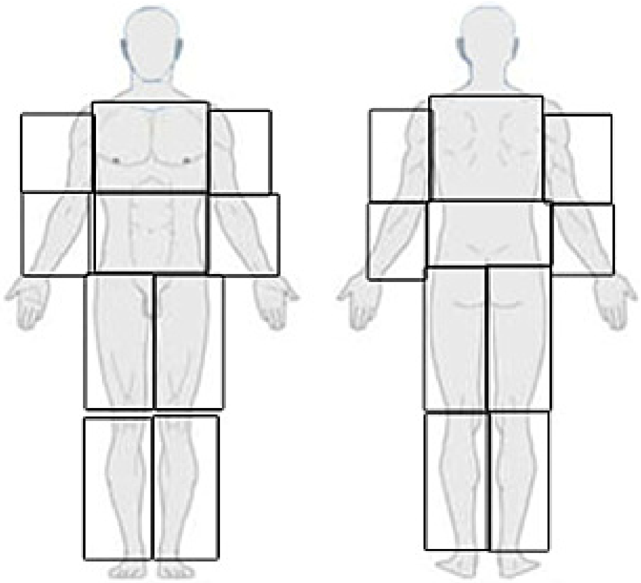

Note: Please mark the representative skin lesion with " √ " in the corresponding box, and record the same skin lesion each time.

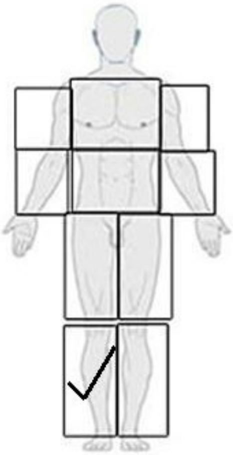

E.g.

Abbreviation of the patient's name |__|__|__|__| Serial number of the enrolment |__|__|__|

Baseline (Week 0)

1 General information

Gender: ☐Male ☐Female

Date of birth: |__|__| (Date) |__|__| (Month) |__|__|__|__| (Year)

Nationality: ☐Han ☐Other_________

Height: |__|__|__|cm **Weight and abdomen circumference**: |__|__|__|.|__|Kg |__|__|__|.|__|cm

Marital status: ☐Married ☐Single ☐Devoiced ☐Widowed

Degree of education: ☐Illiteracy ☐Primary school ☐Junior high school ☐Senior high school or technical secondary school ☐Junior college or bachelor degree ☐Master degree or above

Occupation: ☐Worker ☐Farmer ☐Student ☐Politician ☐Professionals ☐Retired ☐Freelancer ☐ Other: ______

Settlement: ☐Guangdong, China ☐Other:______

Smoking: ☐Yes ☐No

Drinking: ☐Yes ☐No

2 History of allergy: ☐No ☐Yes, please record the following details

Allergy to medication ☐No ☐Yes____

Allergy to food ☐No ☐Yes____

Contact allergy ☐No ☐Yes____

3 Vital signs

Body temperature: |__|__|.|__|℃ Respiratory rate: |__|__| times/min

Heart rate: |__|__|__| Beats/min Blood pressure: |__|__|__|mmHg/|__|__|__|mmHg

4 Physical examination

5 Other diseases and treatments: ☐No ☐Yes, please record the following details

Name of diseases	Active or not	Treatments or not now	Treatments details
	☐ No ☐ Yes	☐ No ☐ Yes	
	☐ No ☐ Yes	☐ No ☐ Yes	
	☐ No ☐ Yes	☐ No ☐ Yes	

Signature of the researcher: ____________ Date: ____________________

6 History of psoriasis

Onset time: |__|__|__|__| Year

Date of initial diagnosis of psoriasis: |__|__|__|__| (Year) Course: |__|__| years or |__|__| months

Pathological diagnosis: ☐ No ☐ Yes, Hospital of pathological diagnosis ____________________

Family history: ☐ No ☐ Unsure ☐ Yes, Relationship with the patients______________________

7 History of treatments

**Topical agents:** ☐ **No** ☐ **Yes, please choose**		Note
☐ Moisturizers: such as Vaseline, Glycerine, Paraffinoma Liquid, Urea cream		
☐ Keratin promoter: such as 2% – 5% coal tar, 3% salicylic acid, 3% – 5% sulphur, 0.1% – 0.5% anthralin, 5% ichthammol		
☐ Keratolytic: such as 5% – 10% salicylic acid, 10% sulphur, 20% urea cream, 5% – 10% lactic acid, 0.1% Retinoids, 10% – 30% ichthammol		
☐ Glucocorticoids: such as hydrocortisone, methylprednisolone, hydrocortisone butyrate, dexamethasone, triamcinolone, mometasone furoate, fluocinolone acetonide, betamethasone valerate, clobetasol propionate, Mometasone furoate		
☐ Retinoids: such as 0.05% – 0.1% tazarotene, 0.1% Adapalene gel		
☐ Vitamin D analogues: such as Calcipotriol, Tacalcitol, Calcitriol		
☐ Calcineurin inhibitors: such as Tacrolimus, Pimecrolimus		
☐ Cytotoxic drugs: 0.05% Chlormethine hydrochloride, Alcoholic solution		
☐ Others: 0.01% – 0.025% Capsaicin ointment, 10% – 15% Camptothecin		
**Phototherapies:** ☐ **No** ☐ **Yes, please choose**		Note
☐ UVA	☐ PUVA	
☐ BB-UVB	☐ NB-UVB	
☐ Excimer laser	☐ Yes, but no details	
**Systemics:** ☐ **No** ☐ **Yes, please choose**		Note
☐ Antibiotics	☐ MTX	
☐ Retinoids	☐ Ciclosporin	
☐ Mycophenolate Mofetil	☐ Hydroxyurea	
☐ Glucocorticoids: such as hydrocortisone, prednisone, prednisolone, methylprednisolone, triancillone, betamethasone, dexamethasone		
☐ Others: such as Tachlimus, Sulfoxycin (sulfoxycin tablet, sulfoxycin enteric coated tablet, sulfoxycin capsule), Levamisole, transfer factors (transfer factor capsule, transfer factor injection), Colcoline		
☐ Biologics: Etanercept, Infliximab, Adalimumab, Ustekinumab, Guselkumab, Secukinumab, Ixekizumab		
☐ Chinese herbal medicine		

Abbreviation of the patient's name |__|__|__|__| Serial number of the enrolment |__|__|__|

**8 Severity of psoriasis**	
BSA:	_________%
PASI:	_________ (Head _____ Upper limbs_____ Trunk _____ Lower limbs ____)
sPGA:	_________ (0-6 point)
DLQI:	_________
Itch VAS:	|__|__|.|__|cm /|__|__|.|__|cm (average/worst)
Skindex-16:	_________

9 Diagnosis and prescription

Diagnosis: Psoriasis vulgaris ☐Other:

Diagnosis in Chinese medicine: *Baibi*

Prescription:

1. CHM external application granules: 14 packages, one package daily
2. 10% Urea cream: |__|__|, twice daily
3. Rescue therapy: ☐ No, ☐ Yes, cetirizine hydrochloride tablets (10 mg) |__|__| tablets
4. Other: ____

Next visit date:_______________   **Signature of the researcher**: _______________

* Electrocardiograms, blood and urine test, fill in itch VAS, DLQI, Skindex-16, take photos for representative skin lesions

* Biological specimens: blood, urine, skin microbiota, stool and oral cavity microbiota

Signature of the researcher: ____________   Date: ________________________

Abbreviation of the patient's name |__|__|__|__| Serial number of the enrolment |__|__|__|

Attached results of chemical examinations at the week 0

Signature of the researcher: ____________ Date: ______________________

Abbreviation of the patient's name |__|__|__|__| Serial number of the enrolment |__|__|__|

Week 2

1 Drop-out

Did the participant drop out?	☐ No<br>☐ Yes, please fill in the form of 'Summary of Trial Completion' (see the page 27)

2 Concomitant medication

Did the participant take any concomitant medication during the period?	☐ No<br>☐ Yes, please fill in the form of 'Records of Concomitant medication' (see the page 28)

3 Adverse events

Is there any adverse event during the period?	☐ No<br>☐ Yes, please fill in the form of 'Reports of Adverse Events' (see the page 29)

4 Trial drugs

Trial drugs use in 2 weeks (|__|__| days):

CHM external application granules actual used: |__|__| packages (use theoretically: |__|__| packages), collected |__|__| packages, compliance rate |__|__|%. *compliance rate = actual used / use theoretically *100%

10% Urea cream: |__|__|

Cetirizine hydrochloride tablet: ☐ No ☐ Yes, used |__|__| tablets

5 Severity of psoriasis

Achieve PASI-50:	☐ Yes ☐ No
Achieve PASI-75:	☐ Yes ☐ No
Itch VAS:	|__|__|.|__|cm /|__|__|.|__|cm (average/worst)

6 Static Physician Global Assessment, sPGA

☐0 Clear	No signs of psoriasis (post inflammatory hyperpigmentation may be present)
☐1 Almost clear	Intermediate between mild and clear
☐2 Mild	Slight plaque elevation, scaling, and/or erythema
☐3 Mild to moderate	Intermediate between moderate and mild
☐4 Moderate	Moderate plaque elevation, scaling, and/or erythema
☐5 Moderate to Severe	Marked plaque elevation, scaling, and/or erythema
☐6 Severe	Very marked plaque elevation, scaling, and/or erythema

Signature of the researcher: ____________ Date: ____________________

7 Psoriasis Area and Severity Index, PASI

	Head (h)	Upper limbs (u)	Trunk (t)	Lower limbs (l)
**Erythema (E)**	☐0 ☐3 ☐1 ☐4 ☐2	☐0 ☐3 ☐1 ☐4 ☐2	☐0 ☐3 ☐1 ☐4 ☐2	☐0 ☐3 ☐1 ☐4 ☐2
**Thickness (D)**	☐0 ☐3 ☐1 ☐4 ☐2	☐0 ☐3 ☐1 ☐4 ☐2	☐0 ☐3 ☐1 ☐4 ☐2	☐0 ☐3 ☐1 ☐4 ☐2
**Scaling (I)**	☐0 ☐3 ☐1 ☐4 ☐2	☐0 ☐3 ☐1 ☐4 ☐2	☐0 ☐3 ☐1 ☐4 ☐2	☐0 ☐3 ☐1 ☐4 ☐2
**Lesion Score Sum (A)**	☐0 = 0%<br>☐1 = 1–9%<br>☐2 = 10–29%<br>☐3 = 30–49%<br>☐4 = 50–69%<br>☐5 = 70–89%<br>☐6 = 90–100%	☐0 = 0%<br>☐1 = 1–9%<br>☐2 = 10–29%<br>☐3 = 30–49%<br>☐4 = 50–69%<br>☐5 = 70–89%<br>☐6 = 90–100%	☐0 = 0%<br>☐1 = 1–9%<br>☐2 = 10–29%<br>☐3 = 30–49%<br>☐4 = 50–69%<br>☐5 = 70–89%<br>☐6 = 90–100%	☐0 = 0%<br>☐1 = 1–9%<br>☐2 = 10–29%<br>☐3 = 30–49%<br>☐4 = 50–69%<br>☐5 = 70–89%<br>☐6 = 90–100%
**Palm areas (one palm ≈ 1% of BSA)**	one palm ≈ 11.1% of head and neck area	one palm ≈ 5.6% of the upper extremities	1 palm ≈ 3.7% of the trunk	1 palm ≈ 2.2% of lower extremities
	______Palms	______Palms	______Palms	______Palms
**BSA**	% (palms of h + palms of u + palms of t + palms of l)			
**Subtotals**	0.1 * Ah (Eh + Ih + Dh) =	0.2 * Au (Eu + Iu + Du) =	0.3 * At (Et+ It + Dt) =	0.4 * Al (El + Il + Dl) =
**PASI totals**				

PASI = 0.1 * Ah (Eh + Ih + Dh) + 0.2 * Au (Eu + Iu + Du) + 0.3 * At (Et+ It + Dt) + 0.4 * Al (El + Il + Dl)

Signature of scorer: ______________

8 Diagnosis and prescription

Diagnosis: Psoriasis vulgaris ☐Other:

Diagnosis in Chinese medicine: ***Baibi***

Prescription:

1. CHM external application granules: 14 packages, one package daily
2. 10% Urea cream: |__|__|, twice daily
3. Rescue therapy: ☐ No, ☐ Yes, cetirizine hydrochloride tablets (10 mg) |__|__| tablets
4. Other: ____

Next visit date:_______________ **Signature of the researcher**: _______________

* Collect trial drugs and diary record sheet * Fill in itch VAS and take photos of representative skin lesions

Abbreviation of the patient's name |__|__|__|__| Serial number of the enrolment |__|__|__|

Week 4

**1 Drop-out**	
Did the participant drop out?	☐ No<br>☐ Yes, please fill in the form of 'Summary of Trial Completion' (see the page 27)
**2 Concomitant medication**	
Did the participant take any concomitant medication during the period?	☐ No<br>☐ Yes, please fill in the form of 'Records of Concomitant medication' (see the page 28)
**3 Adverse events**	
Is there any adverse event during the period?	☐ No<br>☐ Yes, please fill in the form of 'Reports of Adverse Events' (see the page 29)
**4 Blinding credibility**	
Do the participate think the receiving treatment is: ☐ CHM ☐ Placebo ☐ Unsure	
**5 Trial drugs**	
Trial drugs use in 2 weeks (|__|__| days):<br>CHM external application granules actual used: |__|__| packages (use theoretically: |__|__| packages), collected |__|__| packages, compliance rate |__|__|%. *compliance rate = actual used / use theoretically *100%<br>10% Urea cream: |__|__| Cetirizine hydrochloride tablet: ☐ No ☐ Yes, used |__|__| tablets	
**6 Severity of psoriasis**	
Achieve PASI-50:	☐ Yes ☐ No
Achieve PASI-75:	☐ Yes ☐ No
Itch VAS:	|__|__|.|__|cm /|__|__|.|__|cm (average/worst)
**7 Static Physician Global Assessment, sPGA**	
☐0 Clear	No signs of psoriasis (post inflammatory hyperpigmentation may be present)
☐1 Almost clear	Intermediate between mild and clear
☐2 Mild	Slight plaque elevation, scaling, and/or erythema
☐3 Mild to moderate	Intermediate between moderate and mild
☐4 Moderate	Moderate plaque elevation, scaling, and/or erythema
☐5 Moderate to Severe	Marked plaque elevation, scaling, and/or erythema
☐6 Severe	Very marked plaque elevation, scaling, and/or erythema

Signature of the researcher: ____________ Date: ________________

Abbreviation of the patient's name |__|__|__|__| Serial number of the enrolment |__|__|__|

8 Psoriasis Area and Severity Index, PASI

	Head (h)	Upper limbs (u)	Trunk (t)	Lower limbs (l)
**Erythema (E)**	☐0 ☐3 ☐1 ☐4 ☐2	☐0 ☐3 ☐1 ☐4 ☐2	☐0 ☐3 ☐1 ☐4 ☐2	☐0 ☐3 ☐1 ☐4 ☐2
**Thickness (D)**	☐0 ☐3 ☐1 ☐4 ☐2	☐0 ☐3 ☐1 ☐4 ☐2	☐0 ☐3 ☐1 ☐4 ☐2	☐0 ☐3 ☐1 ☐4 ☐2
**Scaling (I)**	☐0 ☐3 ☐1 ☐4 ☐2	☐0 ☐3 ☐1 ☐4 ☐2	☐0 ☐3 ☐1 ☐4 ☐2	☐0 ☐3 ☐1 ☐4 ☐2
**Lesion Score Sum (A)**	☐0 = 0%<br>☐1 = 1–9%<br>☐2 = 10–29%<br>☐3 = 30–49%<br>☐4 = 50–69%<br>☐5 = 70–89%<br>☐6 = 90–100%	☐0 = 0%<br>☐1 = 1–9%<br>☐2 = 10–29%<br>☐3 = 30–49%<br>☐4 = 50–69%<br>☐5 = 70–89%<br>☐6 = 90–100%	☐0 = 0%<br>☐1 = 1–9%<br>☐2 = 10–29%<br>☐3 = 30–49%<br>☐4 = 50–69%<br>☐5 = 70–89%<br>☐6 = 90–100%	☐0 = 0%<br>☐1 = 1–9%<br>☐2 = 10–29%<br>☐3 = 30–49%<br>☐4 = 50–69%<br>☐5 = 70–89%<br>☐6 = 90–100%
**Palm areas (one palm ≈ 1% of BSA)**	one palm ≈ 11.1% of head and neck area	one palm ≈ 5.6% of the upper extremities	1 palm ≈ 3.7% of the trunk	1 palm ≈ 2.2% of lower extremities
	______Palms	______Palms	______Palms	______Palms
**BSA**	% (palms of h + palms of u + palms of t + palms of l)			
**Subtotals**	0.1 * Ah (Eh + Ih + Dh) =	0.2 * Au (Eu + Iu + Du) =	0.3 * At (Et+ It + Dt) =	0.4 * Al (El + Il + Dl) =
**PASI totals**				

PASI = 0.1 * Ah (Eh + Ih + Dh) + 0.2 * Au (Eu + Iu + Du) + 0.3 * At (Et+ It + Dt) + 0.4 * Al (El + Il + Dl)

Signature of scorer: ______________

9 Diagnosis and prescription

Diagnosis: Psoriasis vulgaris ☐Other:

Diagnosis in Chinese medicine: ***Baibi***

Prescription:

1. CHM external application granules: 14 packages, one package daily
2. 10% Urea cream: |__|__|, twice daily
3. Rescue therapy: ☐ No, ☐ Yes, cetirizine hydrochloride tablets (10 mg) |__|__| tablets
4. Other: ____

Next visit date:_______________ **Signature of the researcher**: _______________

* Collect trial drugs and diary record sheet *Fill in itch VAS and take photos for representative skin lesions

Signature of the researcher: _____________ Date: ______________________

Abbreviation of the patient's name |__|__|__|__| Serial number of the enrolment |__|__|__|

Week 6

1 Drop-out

Did the participant drop out?	☐ No<br>☐ Yes, please fill in the form of 'Summary of Trial Completion' (see the page 27)

2 Concomitant medication

Did the participant take any concomitant medication during the period?	☐ No<br>☐ Yes, please fill in the form of 'Records of Concomitant medication' (see the page 28)

3 Adverse events

Is there any adverse event during the period?	☐ No<br>☐ Yes, please fill in the form of 'Reports of Adverse Events' (see the page 29)

4 Trial drugs

Trial drugs use in 2 weeks (|__|__| days)：

CHM external application granules actual used: |__|__| packages (use theoretically: |__|__| packages), collected |__|__| packages, compliance rate |__|__|%. *compliance rate = actual used / use theoretically *100%

10% Urea cream: |__|__|

Cetirizine hydrochloride tablet: ☐ No ☐ Yes, used |__|__| tablets

5 Severity of psoriasis

Achieve PASI-50:	☐ Yes ☐ No
Achieve PASI-75:	☐ Yes ☐ No
Itch VAS：	|__|__|.|__|cm /|__|__|.|__|cm (average/worst)

6 Static Physician Global Assessment, sPGA

☐0 Clear	No signs of psoriasis (post inflammatory hyperpigmentation may be present)
☐1 Almost clear	Intermediate between mild and clear
☐2 Mild	Slight plaque elevation, scaling, and/or erythema
☐3 Mild to moderate	Intermediate between moderate and mild
☐4 Moderate	Moderate plaque elevation, scaling, and/or erythema
☐5 Moderate to Severe	Marked plaque elevation, scaling, and/or erythema
☐6 Severe	Very marked plaque elevation, scaling, and/or erythema

Signature of the researcher: ____________ Date: ____________________

7 Psoriasis Area and Severity Index, PASI				
	**Head (h)**	**Upper limbs (u)**	**Trunk (t)**	**Lower limbs (l)**
**Erythema (E)**	☐0 ☐3 ☐1 ☐4 ☐2	☐0 ☐3 ☐1 ☐4 ☐2	☐0 ☐3 ☐1 ☐4 ☐2	☐0 ☐3 ☐1 ☐4 ☐2
**Thickness (D)**	☐0 ☐3 ☐1 ☐4 ☐2	☐0 ☐3 ☐1 ☐4 ☐2	☐0 ☐3 ☐1 ☐4 ☐2	☐0 ☐3 ☐1 ☐4 ☐2
**Scaling (I)**	☐0 ☐3 ☐1 ☐4 ☐2	☐0 ☐3 ☐1 ☐4 ☐2	☐0 ☐3 ☐1 ☐4 ☐2	☐0 ☐3 ☐1 ☐4 ☐2
**Lesion Score Sum (A)**	☐0 = 0%<br>☐1 = 1–9%<br>☐2 = 10–29%<br>☐3 = 30–49%<br>☐4 = 50–69%<br>☐5 = 70–89%<br>☐6 = 90–100%	☐0 = 0%<br>☐1 = 1–9%<br>☐2 = 10–29%<br>☐3 = 30–49%<br>☐4 = 50–69%<br>☐5 = 70–89%<br>☐6 = 90–100%	☐0 = 0%<br>☐1 = 1–9%<br>☐2 = 10–29%<br>☐3 = 30–49%<br>☐4 = 50–69%<br>☐5 = 70–89%<br>☐6 = 90–100%	☐0 = 0%<br>☐1 = 1–9%<br>☐2 = 10–29%<br>☐3 = 30–49%<br>☐4 = 50–69%<br>☐5 = 70–89%<br>☐6 = 90–100%
**Palm areas (one palm ≈ 1% of BSA)**	one palm ≈ 11.1% of head and neck area	one palm ≈ 5.6% of the upper extremities	1 palm ≈ 3.7% of the trunk	1 palm ≈ 2.2% of lower extremities
	______Palms	______Palms	______Palms	______Palms
**BSA**	% (palms of h + palms of u + palms of t + palms of l)			
**Subtotals**	0.1 * Ah (Eh + Ih + Dh) =	0.2 * Au (Eu + Iu + Du) =	0.3 * At (Et+ It + Dt) =	0.4 * Al (El + Il + Dl) =
**PASI totals**				
PASI = 0.1 * Ah (Eh + Ih + Dh) + 0.2 * Au (Eu + Iu + Du) + 0.3 * At (Et+ It + Dt) + 0.4 * Al (El + Il + Dl)				
**Signature of scorer:** ____________				

8 Diagnosis and prescription

Diagnosis: Psoriasis vulgaris ☐Other:

Diagnosis in Chinese medicine: ***Baibi***

Prescription:

1. CHM external application granules: 14 packages, one package daily
2. 10% Urea cream: |__|__|, twice daily
3. Rescue therapy: ☐ No, ☐ Yes, cetirizine hydrochloride tablets (10 mg) |__|__| tablets
4. Other: ____

Next visit date:______________　　**Signature of the researcher**: ______________

* Collect trial drugs and diary record sheet * Fill in itch VAS and take photos of representative skin lesions

Abbreviation of the patient's name |__|__|__|__| Serial number of the enrolment |__|__|__|

Week 8

1 Drop-out

Did the participant drop out?
☐ No
☐ Yes, please fill in the form of 'Summary of Trial Completion' (see the page 27)

2 Concomitant medication

Did the participant take any concomitant medication during the period?
☐ No
☐ Yes, please fill in the form of 'Records of Concomitant medication' (see the page 28)

3 Adverse events

Is there any adverse event during the period?
☐ No
☐ Yes, please fill in the form of 'Reports of Adverse Events' (see the page 29)

4 Blinding credibility

Do the participate think the receiving treatment is: ☐ CHM ☐ Placebo ☐ Unsure

5 Vital signs

Body temperature: |__|__|.|__|℃ Respiratory rate: |__|__| times/min

Heart rate: |__|__|__| Beats/min Blood pressure: |__|__|__|mmHg/|__|__|__|mmHg

6 Physical examination

7 Trial drugs

Trial drugs use in 2 weeks (|__|__| days): CHM external application granules actual used: |__|__| packages (use theoretically: |__|__| packages), collected |__|__| packages, compliance rate |__|__|%. *compliance rate = actual used / use theoretically *100%

10% Urea cream: |__|__|

Cetirizine hydrochloride tablet: ☐ No ☐ Yes, used |__|__| tablets

8 Severity of psoriasis

Achieve PASI-50:	☐ Yes ☐ No	DLQI: __________	Skindex-16: __________
Achieve PASI-75:	☐ Yes ☐ No	11-point ordinal scale: __________	
Itch VAS:	|__|__|.|__|cm /|__|__|.|__|cm (average/worst)		

9 Static Physician Global Assessment, sPGA

☐0 Clear	No signs of psoriasis (post inflammatory hyperpigmentation may be present)
☐1 Almost clear	Intermediate between mild and clear
☐2 Mild	Slight plaque elevation, scaling, and/or erythema
☐3 Mild to moderate	Intermediate between moderate and mild
☐4 Moderate	Moderate plaque elevation, scaling, and/or erythema
☐5 Moderate to Severe	Marked plaque elevation, scaling, and/or erythema
☐6 Severe	Very marked plaque elevation, scaling, and/or erythema

Signature of the researcher: ____________ Date: ____________________

10 Psoriasis Area and Severity Index, PASI				
	**Head (h)**	**Upper limbs (u)**	**Trunk (t)**	**Lower limbs (l)**
**Erythema (E)**	☐0 ☐3 ☐1 ☐4 ☐2	☐0 ☐3 ☐1 ☐4 ☐2	☐0 ☐3 ☐1 ☐4 ☐2	☐0 ☐3 ☐1 ☐4 ☐2
**Thickness (D)**	☐0 ☐3 ☐1 ☐4 ☐2	☐0 ☐3 ☐1 ☐4 ☐2	☐0 ☐3 ☐1 ☐4 ☐2	☐0 ☐3 ☐1 ☐4 ☐2
**Scaling (I)**	☐0 ☐3 ☐1 ☐4 ☐2	☐0 ☐3 ☐1 ☐4 ☐2	☐0 ☐3 ☐1 ☐4 ☐2	☐0 ☐3 ☐1 ☐4 ☐2
**Lesion Score Sum (A)**	☐0 = 0%<br>☐1 = 1–9%<br>☐2 = 10–29%<br>☐3 = 30–49%<br>☐4 = 50–69%<br>☐5 = 70–89%<br>☐6 = 90–100%	☐0 = 0%<br>☐1 = 1–9%<br>☐2 = 10–29%<br>☐3 = 30–49%<br>☐4 = 50–69%<br>☐5 = 70–89%<br>☐6 = 90–100%	☐0 = 0%<br>☐1 = 1–9%<br>☐2 = 10–29%<br>☐3 = 30–49%<br>☐4 = 50–69%<br>☐5 = 70–89%<br>☐6 = 90–100%	☐0 = 0%<br>☐1 = 1–9%<br>☐2 = 10–29%<br>☐3 = 30–49%<br>☐4 = 50–69%<br>☐5 = 70–89%<br>☐6 = 90–100%
**Palm areas (one palm ≈ 1% of BSA)**	one palm ≈ 11.1% of head and neck area	one palm ≈ 5.6% of the upper extremities	1 palm ≈ 3.7% of the trunk	1 palm ≈ 2.2% of lower extremities
	______Palms	______Palms	______Palms	______Palms
**BSA**	% (palms of h + palms of u + palms of t + palms of l)			
**Subtotals**	0.1 * Ah (Eh + Ih + Dh) =	0.2 * Au (Eu + Iu + Du) =	0.3 * At (Et+ It + Dt) =	0.4 * Al (El + Il + Dl) =
**PASI totals**				
PASI = 0.1 * Ah (Eh + Ih + Dh) + 0.2 * Au (Eu + Iu + Du) + 0.3 * At (Et+ It + Dt) + 0.4 * Al (El + Il + Dl)				
**Signature of scorer:** ______________				

11 Diagnosis and prescription

Diagnosis: Psoriasis vulgaris ☐**Other:**

Diagnosis in Chinese medicine: ***Baibi***

Prescription:

The end of the treatment phases and stop using trial drugs.

Next visit date:_______________ **Signature of the researcher**: _______________

* Collect trial drugs and diary record sheet

* Blood and urine test, fill in itch VAS, DLQI, Skindex-16, and 11-point ordinal scale, take photos for representative skin lesions

* Biological specimens: blood, urine and skin microbiota

Abbreviation of the patient's name |__|__|__|__| Serial number of the enrolment |__|__|__|

Attached results of chemical examinations at the week 8

Signature of the researcher: ____________   Date: ____________________

Abbreviation of the patient's name |__|__|__|__| Serial number of the enrolment |__|__|__|

Week 12

**1 Drop-out**	
Did the participant drop out?	☐ No<br>☐ Yes, please fill in the form of 'Summary of Trial Completion' (see the page 27)
**2 Concomitant medication**	
Did the participant take any concomitant medication during the period?	☐ No<br>☐ Yes, please fill in the form of 'Records of Concomitant medication' (see the page 28)
**3 Adverse events**	
Is there any adverse event during the period?	☐ No<br>☐ Yes, please fill in the form of 'Reports of Adverse Events' (see the page 29)
**4 Severity of psoriasis**	
Relapse:	☐ No<br>☐ Yes, factors: ☐fatigue ☐Diet ☐Infection ☐Menstruation ☐ Drugs ☐Seasons change ☐Trauma surgery ☐Mental factors (e.g., tension, stress, sadness, anxiety) ☐Other:______________
Rebound:	☐ No<br>☐ Yes, factors: ☐fatigue ☐Diet ☐Infection ☐Menstruation ☐ Drugs ☐Seasons change ☐Trauma surgery ☐Mental factors (e.g., tension, stress, sadness, anxiety) ☐Other:______________
Itch VAS:	|__|__|.|__|cm /|__|__|.|__|cm (average/worst)
**5 Static Physician Global Assessment, sPGA**	
☐0 Clear	No signs of psoriasis (post inflammatory hyperpigmentation may be present)
☐1 Almost clear	Intermediate between mild and clear
☐2 Mild	Slight plaque elevation, scaling, and/or erythema
☐3 Mild to moderate	Intermediate between moderate and mild
☐4 Moderate	Moderate plaque elevation, scaling, and/or erythema
☐5 Moderate to Severe	Marked plaque elevation, scaling, and/or erythema
☐6 Severe	Very marked plaque elevation, scaling, and/or erythema

Signature of the researcher: ____________ Date: ____________________

6 Psoriasis Area and Severity Index, PASI				
	**Head (h)**	**Upper limbs (u)**	**Trunk (t)**	**Lower limbs (l)**
**Erythema (E)**	☐0 ☐3 ☐1 ☐4 ☐2	☐0 ☐3 ☐1 ☐4 ☐2	☐0 ☐3 ☐1 ☐4 ☐2	☐0 ☐3 ☐1 ☐4 ☐2
**Thickness (D)**	☐0 ☐3 ☐1 ☐4 ☐2	☐0 ☐3 ☐1 ☐4 ☐2	☐0 ☐3 ☐1 ☐4 ☐2	☐0 ☐3 ☐1 ☐4 ☐2
**Scaling (I)**	☐0 ☐3 ☐1 ☐4 ☐2	☐0 ☐3 ☐1 ☐4 ☐2	☐0 ☐3 ☐1 ☐4 ☐2	☐0 ☐3 ☐1 ☐4 ☐2
**Lesion Score Sum (A)**	☐0 = 0%<br>☐1 = 1–9%<br>☐2 = 10–29%<br>☐3 = 30–49%<br>☐4 = 50–69%<br>☐5 = 70–89%<br>☐6 = 90–100%	☐0 = 0%<br>☐1 = 1–9%<br>☐2 = 10–29%<br>☐3 = 30–49%<br>☐4 = 50–69%<br>☐5 = 70–89%<br>☐6 = 90–100%	☐0 = 0%<br>☐1 = 1–9%<br>☐2 = 10–29%<br>☐3 = 30–49%<br>☐4 = 50–69%<br>☐5 = 70–89%<br>☐6 = 90–100%	☐0 = 0%<br>☐1 = 1–9%<br>☐2 = 10–29%<br>☐3 = 30–49%<br>☐4 = 50–69%<br>☐5 = 70–89%<br>☐6 = 90–100%
**Palm areas (one palm ≈ 1% of BSA)**	one palm ≈ 11.1% of head and neck area	one palm ≈ 5.6% of the upper extremities	1 palm ≈ 3.7% of the trunk	1 palm ≈ 2.2% of lower extremities
	______Palms	______Palms	______Palms	______Palms
**BSA**	% (palms of h + palms of u + palms of t + palms of l)			
**Subtotals**	0.1 * Ah (Eh + Ih + Dh) =	0.2 * Au (Eu + Iu + Du) =	0.3 * At (Et+ It + Dt) =	0.4 * Al (El + Il + Dl) =
**PASI totals**				
PASI = 0.1 * Ah (Eh + Ih + Dh) + 0.2 * Au (Eu + Iu + Du) + 0.3 * At (Et+ It + Dt) + 0.4 * Al (El + Il + Dl)				
**Signature of scorer:** ____________				

7 Diagnosis and prescription
**Diagnosis: Psoriasis vulgaris** ☐**Other:**<br>**Diagnosis in Chinese medicine:** ***Baibi***<br>**Prescription:**<br><br>**Next visit date:**______________ **Signature of the researcher**: ______________<br>* Collect diary record sheet<br>* Fill in itch VAS and take photos for representative skin lesions

Abbreviation of the patient's name |__|__|__|__| Serial number of the enrolment |__|__|__|

Week 16

**1 Drop-out**	
Did the participant drop out?	☐ No<br>☐ Yes, please fill in the form of 'Summary of Trial Completion' (see the page 27)
**2 Concomitant medication**	
Did the participant take any concomitant medication during the period?	☐ No<br>☐ Yes, please fill in the form of 'Records of Concomitant medication' (see the page 28)
**3 Adverse events**	
Is there any adverse event during the period?	☐ No<br>☐ Yes, please fill in the form of 'Reports of Adverse Events' (see the page 29)
**4 Severity of psoriasis**	
Relapse:	☐ No<br>☐ Yes, factors: ☐fatigue ☐Diet ☐Infection ☐Menstruation ☐ Drugs ☐Seasons change ☐Trauma surgery ☐Mental factors (e.g., tension, stress, sadness, anxiety) ☐Other:______________
Rebound:	☐ No<br>☐ Yes, factors: ☐fatigue ☐Diet ☐Infection ☐Menstruation ☐ Drugs ☐Seasons change ☐Trauma surgery ☐Mental factors (e.g., tension, stress, sadness, anxiety) ☐Other:______________
Itch VAS：	|__|__|.|__|cm /|__|__|.|__|cm (average/worst)
**5 Static Physician Global Assessment, sPGA**	
☐0 Clear	No signs of psoriasis (post inflammatory hyperpigmentation may be present)
☐1 Almost clear	Intermediate between mild and clear
☐2 Mild	Slight plaque elevation, scaling, and/or erythema
☐3 Mild to moderate	Intermediate between moderate and mild
☐4 Moderate	Moderate plaque elevation, scaling, and/or erythema
☐5 Moderate to Severe	Marked plaque elevation, scaling, and/or erythema
☐6 Severe	Very marked plaque elevation, scaling, and/or erythema

Signature of the researcher: ____________ Date: ____________________

6 Psoriasis Area and Severity Index, PASI				
	**Head (h)**	**Upper limbs (u)**	**Trunk (t)**	**Lower limbs (l)**
**Erythema (E)**	☐0 ☐3 ☐1 ☐4 ☐2	☐0 ☐3 ☐1 ☐4 ☐2	☐0 ☐3 ☐1 ☐4 ☐2	☐0 ☐3 ☐1 ☐4 ☐2
**Thickness (D)**	☐0 ☐3 ☐1 ☐4 ☐2	☐0 ☐3 ☐1 ☐4 ☐2	☐0 ☐3 ☐1 ☐4 ☐2	☐0 ☐3 ☐1 ☐4 ☐2
**Scaling (I)**	☐0 ☐3 ☐1 ☐4 ☐2	☐0 ☐3 ☐1 ☐4 ☐2	☐0 ☐3 ☐1 ☐4 ☐2	☐0 ☐3 ☐1 ☐4 ☐2
**Lesion Score Sum (A)**	☐0 = 0%<br>☐1 = 1–9%<br>☐2 = 10–29%<br>☐3 = 30–49%<br>☐4 = 50–69%<br>☐5 = 70–89%<br>☐6 = 90–100%	☐0 = 0%<br>☐1 = 1–9%<br>☐2 = 10–29%<br>☐3 = 30–49%<br>☐4 = 50–69%<br>☐5 = 70–89%<br>☐6 = 90–100%	☐0 = 0%<br>☐1 = 1–9%<br>☐2 = 10–29%<br>☐3 = 30–49%<br>☐4 = 50–69%<br>☐5 = 70–89%<br>☐6 = 90–100%	☐0 = 0%<br>☐1 = 1–9%<br>☐2 = 10–29%<br>☐3 = 30–49%<br>☐4 = 50–69%<br>☐5 = 70–89%<br>☐6 = 90–100%
**Palm areas (one palm ≈ 1% of BSA)**	one palm ≈ 11.1% of head and neck area	one palm ≈ 5.6% of the upper extremities	1 palm ≈ 3.7% of the trunk	1 palm ≈ 2.2% of lower extremities
	______Palms	______Palms	______Palms	______Palms
**BSA**	% (palms of h + palms of u + palms of t + palms of l)			
**Subtotals**	0.1 * Ah (Eh + Ih + Dh) =	0.2 * Au (Eu + Iu + Du) =	0.3 * At (Et+ It + Dt) =	0.4 * Al (El + Il + Dl) =
**PASI totals**				
PASI = 0.1 * Ah (Eh + Ih + Dh) + 0.2 * Au (Eu + Iu + Du) + 0.3 * At (Et+ It + Dt) + 0.4 * Al (El + Il + Dl)				
**Signature of scorer:** ____________				

7 Diagnosis and prescription

Diagnosis: Psoriasis vulgaris ☐**Other:**

Diagnosis in Chinese medicine: ***Baibi***

Prescription:

Next visit date:______________   **Signature of the researcher**: ______________

* Collect diary record sheet

* Fill in itch VAS and take photos for representative skin lesions

Abbreviation of the patient's name |__|__|__|__| Serial number of the enrolment |__|__|__|

Week 20

1 Drop-out

Did the participant drop out?	☐ No<br>☐ Yes, please fill in the form of 'Summary of Trial Completion' (see the page 27)

2 Concomitant medication

Did the participant take any concomitant medication during the period?	☐ No<br>☐ Yes, please fill in the form of 'Records of Concomitant medication' (see the page 28)

3 Adverse events

Is there any adverse event during the period?	☐ No<br>☐ Yes, please fill in the form of 'Reports of Adverse Events' (see the page 29)

4 Blinding credibility

Do the participate think the receiving treatment is: ☐ CHM ☐ Placebo ☐ Unsure

5 Vital signs

Body temperature: |__|__|.|__|℃ Respiratory rate: |__|__| times/min

Heart rate: |__|__|__| Beats/min Blood pressure: |__|__|__|mmHg/|__|__|__|mmHg

6 Physical examination

7 Severity of psoriasis

Relapse:	☐ No<br>☐ Yes, factors: ☐fatigue ☐Diet ☐Infection ☐Menstruation ☐ Drugs ☐Seasons change ☐Trauma surgery ☐Mental factors (e.g., tension, stress, sadness, anxiety) ☐Other:______________
Rebound:	☐ No<br>☐ Yes, factors: ☐fatigue ☐Diet ☐Infection ☐Menstruation ☐ Drugs ☐Seasons change ☐Trauma surgery ☐Mental factors (e.g., tension, stress, sadness, anxiety) ☐Other:______________
Itch VAS:	|__|__|.|__|cm /|__|__|.|__|cm (average/worst)

DLQI: _________ Skindex-16: _________ 11-point ordinal scale: _________

8 Static Physician Global Assessment, sPGA

☐0 Clear	No signs of psoriasis (post inflammatory hyperpigmentation may be present)
☐1 Almost clear	Intermediate between mild and clear
☐2 Mild	Slight plaque elevation, scaling, and/or erythema
☐3 Mild to moderate	Intermediate between moderate and mild
☐4 Moderate	Moderate plaque elevation, scaling, and/or erythema
☐5 Moderate to Severe	Marked plaque elevation, scaling, and/or erythema
☐6 Severe	Very marked plaque elevation, scaling, and/or erythema

Signature of the researcher: ___________ Date: ________________

9 Psoriasis Area and Severity Index, PASI

	**Head (h)**	**Upper limbs (u)**	**Trunk (t)**	**Lower limbs (l)**
**Erythema (E)**	☐0<br>☐3<br>☐1<br>☐4<br>☐2	☐0<br>☐3<br>☐1<br>☐4<br>☐2	☐0<br>☐3<br>☐1<br>☐4<br>☐2	☐0<br>☐3<br>☐1<br>☐4<br>☐2
**Thickness (D)**	☐0<br>☐3<br>☐1<br>☐4<br>☐2	☐0<br>☐3<br>☐1<br>☐4<br>☐2	☐0<br>☐3<br>☐1<br>☐4<br>☐2	☐0<br>☐3<br>☐1<br>☐4<br>☐2
**Scaling (I)**	☐0<br>☐3<br>☐1<br>☐4<br>☐2	☐0<br>☐3<br>☐1<br>☐4<br>☐2	☐0<br>☐3<br>☐1<br>☐4<br>☐2	☐0<br>☐3<br>☐1<br>☐4<br>☐2
**Lesion Score Sum (A)**	☐0 = 0%<br>☐1 = 1–9%<br>☐2 = 10–29%<br>☐3 = 30–49%<br>☐4 = 50–69%<br>☐5 = 70–89%<br>☐6 = 90–100%	☐0 = 0%<br>☐1 = 1–9%<br>☐2 = 10–29%<br>☐3 = 30–49%<br>☐4 = 50–69%<br>☐5 = 70–89%<br>☐6 = 90–100%	☐0 = 0%<br>☐1 = 1–9%<br>☐2 = 10–29%<br>☐3 = 30–49%<br>☐4 = 50–69%<br>☐5 = 70–89%<br>☐6 = 90–100%	☐0 = 0%<br>☐1 = 1–9%<br>☐2 = 10–29%<br>☐3 = 30–49%<br>☐4 = 50–69%<br>☐5 = 70–89%<br>☐6 = 90–100%
**Palm areas (one palm ≈ 1% of BSA)**	one palm ≈ 11.1% of head and neck area	one palm ≈ 5.6% of the upper extremities	1 palm ≈ 3.7% of the trunk	1 palm ≈ 2.2% of lower extremities
	______Palms	______Palms	______Palms	______Palms
**BSA**	% (palms of h + palms of u + palms of t + palms of l)			
**Subtotals**	0.1 * Ah (Eh + Ih + Dh) =	0.2 * Au (Eu + Iu + Du) =	0.3 * At (Et+ It + Dt) =	0.4 * Al (El + Il + Dl) =
**PASI totals**				

PASI = 0.1 * Ah (Eh + Ih + Dh) + 0.2 * Au (Eu + Iu + Du) + 0.3 * At (Et+ It + Dt) + 0.4 * Al (El + Il + Dl)

Signature of scorer: ______________

10 Diagnosis and prescription

Diagnosis: Psoriasis vulgaris ☐**Other:**

Diagnosis in Chinese medicine: ***Baibi***

Prescription:

The end of the follow-up phase.

Signature of the researcher: ______________

* Collect diary record sheet

* Blood and urine test, fill in itch VAS, DLQI, Skindex-16, and 11-point ordinal scale, take photos for representative skin lesions

* Biological specimens: blood, urine and skin microbiota

Abbreviation of the patient's name |__|__|__|__| Serial number of the enrolment |__|__|__|

Attached results of chemical examinations at the week 20

Signature of the researcher: ____________ Date: ______________________

Abbreviation of the patient's name |__|__|__|__| Serial number of the enrolment |__|__|__|

Summary of trial completion

1.1 Did the participant complete the treatment phases:	☐ Yes<br>☐ No, please complete the following blanks
1.2 Did the participant complete the follow-up phases:	☐ Yes<br>☐ No, please complete the following blanks
2 Date of withdrawal/termination:	|__|__| Day |__|__| Months 202|__| Year

➢ Withdrawal/termination was first raised by (Please tick only one option):

☐ Investigator ☐ Participant

☐ Other: ____________________

➢ Reason for withdrawal/termination (Please tick only one option):

☐ Adverse events ☐ No or unsatisfied effects

☐ Lost to follow-up ☐ Violate the trial protocol

☐ Others: ____________________

3 Did the participant be broken the randomization code?

☐ No

☐ Yes, date of breaking the randomization code ____________

Reasons: ____________________

Follow-up observation or visits after withdrawal/ termination of adverse events (Please provide details of the process and outcomes of the adverse events):____________________

Signature of the researcher: ____________ Date: ____________

Abbreviation of the patient's name |__|__|__|__| Serial number of the enrolment |__|__|__|

Records of concomitant medication

Did the participant take any concomitant medication during the period?	☐ No<br>☐ Yes, please fill in the form.				
The brand name	Disease	Usage daily	Start date	End date	Ongoing
					☐
					☐
					☐
					☐
					☐
					☐
					☐
					☐
					☐
					☐
					☐
					☐
					☐
					☐
					☐
					☐
					☐
					☐
					☐

Signature of the researcher: ____________ Date: ____________________

Abbreviation of the patient's name |__|__|__|__| Serial number of the enrolment |__|__|__|

Adverse event report form

**Adverse event Report form**	AE: ☐ No ☐ Yes, please fill in the form as below:	
Please record all observed (including directly inquired) adverse events (including symptoms, signs, clinical tests, etc.) and their management in **standard medical terms**, one adverse event per column (attached page).		
Name and Description [1]		
Start date	20______/______/______ ________:________ (a 24-hour system)	20______/______/______ ________:________ (a 24-hour system)
End date [2]	20______/______/______ ________:________ (a 24-hour system)	20______/______/______ ________:________ (a 24-hour system)
Severity [3]	☐ Grade1 ☐ Grade 2 ☐ Grade 3 ☐ Grade 4 ☐ Grade 5	☐ Grade1 ☐ Grade 2 ☐ Grade 3 ☐ Grade 4 ☐ Grade 5
Management	☐ No<br>☐ Yes, please choose:<br>Trial medications: ☐ the same dosage ☐ increase dosage ☐decrease dosage ☐ suspended use ☐ permanently suspended use ☐ Not Applicable ☐ unknown<br>Concomitant medications: ☐ No, ☐ Yes, please fill in the combined drug usage record sheet	☐ No<br>☐ Yes, please choose:<br>Trial medications: ☐ the same dosage ☐ increase dosage ☐decrease dosage ☐ suspended use ☐ permanently suspended use ☐ Not Applicable ☐ unknown<br>Concomitant medications: ☐ No, ☐ Yes, please fill in the combined drug usage record sheet
Causality assessment with study drug	☐ Certain ☐ Probable/ Likely ☐ Possible ☐ Unlikely ☐ Conditional/ Unclassified ☐ Unclassifiable	☐ Certain ☐ Probable/ Likely ☐ Possible ☐ Unlikely ☐ Conditional/ Unclassified ☐ Unclassifiable
Follow-up	☐ Fatal ☐ Persist ☐ Recovered/Resolved ☐ Recovered/Resolved with sequelae ☐ Recovering/Resolving ☐ Unknown	☐ Fatal ☐ Persist ☐ Recovered/Resolved ☐ Recovered/Resolved with sequelae ☐ Recovering/Resolving ☐ Unknown
Breaking the randomization code	☐ No ☐ Yes	☐ No ☐ Yes
Withdrawal from the trial	☐ No ☐ Yes	☐ No ☐ Yes
Severe AEs	☐ No<br>☐ Yes, ☐ Results in death ☐ Is life-threatening ☐ Requires hospitalisation or prolongation of existing hospitalisation<br>☐ Results in disability/incapacity: a substantial disruption of a person's ability to conduct normal life functions<br>☐ Results in a congenital anomaly/birth defect<br>☐ Result in other important medical events	☐ No<br>☐ Yes, ☐ Results in death ☐ Is life-threatening ☐ Requires hospitalisation or prolongation of existing hospitalisation<br>☐ Results in disability/incapacity: a substantial disruption of a person's ability to conduct normal life functions<br>☐ Results in a congenital anomaly/birth defect<br>☐ Result in other important medical events

Note: 1 refers to CTCAE v5.0

2 If adverse events persist, do not specify the end time

3 Grade 1: Mild; asymptomatic or mild symptoms, clinical or diagnostic observations only; intervention not indicated; Grade 2: Moderate; minimal, local, or non-invasive intervention indicated; limiting age-appropriate instrumental activities of daily living; Grade 3: Severe or medically significant but not immediately life-threatening; hospitalization or prolongation of hospitalization indicated; disabling; limiting self-care activities of daily living; Grade 4: Life threatening consequences; urgent intervention indicated; Grade 5: Death related to adverse event

4. If the form is not enough to fill in, please copy and add pages.

Signature of the researcher: ____________   Date: ____________________

Abbreviation of the patient's name |__|__|__|__| Serial number of the enrolment |__|__|__|

Adverse Event Report Form

**Adverse event Report form** AE: ☐ No ☐ Yes, please fill in the form as below：		
Please record all observed (including directly inquired) adverse events (including symptoms, signs, clinical tests, etc.) and their management in **standard medical terms**, one adverse event per column (attached page).		
Name and Description [1]		
Start date	20______/______/______ ________:________ (a 24-hour system)	20______/______/______ ________:________ (a 24-hour system)
End date [2]	20______/______/______ ________:________ (a 24-hour system)	20______/______/______ ________:________ (a 24-hour system)
Severity [3]	☐ Grade1 ☐ Grade 2 ☐ Grade 3 ☐ Grade 4 ☐ Grade 5	☐ Grade1 ☐ Grade 2 ☐ Grade 3 ☐ Grade 4 ☐ Grade 5
Management	☐ No<br>☐ Yes, please choose:<br>Trial medications: ☐ the same dosage ☐ increase dosage ☐decrease dosage ☐ suspended use ☐ permanently suspended use ☐ Not Applicable ☐ unknown<br>Concomitant medications: ☐ No, ☐ Yes, please fill in the combined drug usage record sheet	☐ No<br>☐ Yes, please choose:<br>Trial medications: ☐ the same dosage ☐ increase dosage ☐decrease dosage ☐ suspended use ☐ permanently suspended use ☐ Not Applicable ☐ unknown<br>Concomitant medications: ☐ No, ☐ Yes, please fill in the combined drug usage record sheet
Causality assessment with study drug	☐ Certain ☐ Probable/ Likely ☐ Possible ☐ Unlikely ☐ Conditional/ Unclassified ☐ Unclassifiable	☐ Certain ☐ Probable/ Likely ☐ Possible ☐ Unlikely ☐ Conditional/ Unclassified ☐ Unclassifiable
Follow-up	☐ Fatal ☐ Persist ☐ Recovered/Resolved ☐ Recovered/Resolved with sequelae ☐ Recovering/Resolving ☐ Unknown	☐ Fatal ☐ Persist ☐ Recovered/Resolved ☐ Recovered/Resolved with sequelae ☐ Recovering/Resolving ☐ Unknown
Breaking the randomization code	☐ No ☐ Yes	☐ No ☐ Yes
Withdrawal from the trial	☐ No ☐ Yes	☐ No ☐ Yes
Severe AEs	☐ No<br>☐ Yes, ☐ Results in death ☐ Is life-threatening ☐ Requires hospitalisation or prolongation of existing hospitalisation<br>☐ Results in disability/incapacity: a substantial disruption of a person's ability to conduct normal life functions<br>☐ Results in a congenital anomaly/birth defect<br>☐ Result in other important medical events	☐ No<br>☐ Yes, ☐ Results in death ☐ Is life-threatening ☐ Requires hospitalisation or prolongation of existing hospitalisation<br>☐ Results in disability/incapacity: a substantial disruption of a person's ability to conduct normal life functions<br>☐ Results in a congenital anomaly/birth defect<br>☐ Result in other important medical events

Note: 1 refers to CTCAE v5.0

2 If adverse events persist, do not specify the end time

3 Grade 1: Mild; asymptomatic or mild symptoms, clinical or diagnostic observations only; intervention not indicated; Grade 2: Moderate; minimal, local, or non-invasive intervention indicated; limiting age-appropriate instrumental activities of daily living; Grade 3: Severe or medically significant but not immediately life-threatening; hospitalization or prolongation of hospitalization indicated; disabling; limiting self-care activities of daily living; Grade 4: Life threatening consequences; urgent intervention indicated; Grade 5: Death related to adverse event

4. If the form is not enough to fill in, please copy and add pages.

Signature of the researcher: ____________ Date: ____________________

Review statement of the case report form

As the principal investigator of the trial, I declare that all of research data recorded in the case report form is true and accurate after reviewing.

Signature of the principal investigator:_____________

Date: ____________________

As the monitor of the trial, I declare that all of research data recorded in the case report form is consistent with the medical case, as well as is true and accurate after reviewing.

Signature of the trial monitor:_____________

Date: ____________________

Abbreviation of the patient's name |__|__|__|__| Serial number of the enrolment |__|__|__|

Schedule for the trial

Note: Please mark a position with a cross (×) in the "○" when you complete an item in the trial visit.

	Phase	Treatment					Follow-up		
	**Trial visit**	**Baseline (Week 0)**	**Week 2**	**Week 4**	**Week 6**	**Week 8**	**Week 12**	**Week 16**	**Week 20**
**Research activities**	Informed consent	○							
	Randomisation	○							
	ECG, blood test, urine test	○							
	Photos taken for representative skin lesions	○	○	○	○	○	○	○	○
	Dispense trial medications	○	○	○	○				
	Collect trial medications		○	○	○	○			
	Dispense home diary record sheet	○	○	○	○	○	○	○	
	Collect home diary record sheet		○	○	○	○	○	○	○
**Data collection: general information**	Demographics, medical history	○							
	Concomitant medication	○	○	○	○	○	○	○	○
**Data collection: efficacy assessment**	PASI, BSA, sPGA, Itch VAS	○	○	○	○	○	○	○	○
	DLQI, Skindex-16	○				○			○
**Data collection: safety assessment**	Vital signs, physical examination	○				○			○
	Blood test, urine test					○			○
	Reporting AEs	○	○	○	○	○	○	○	○
**Data collection: feasibility assessment**	Trial medication usage check		○	○	○	○			
	Acceptability assessment					○			○
	Blinding credibility			○		○			○
**Data collection: biological specimens**	Blood, urine, skin microbiota	○				○			○
	Stool and oral cavity microbiota	○							

Note: AE, adverse event(s); BSA, Body Surface Area; DLQI, Dermatology Life Quality Index; ECG, electrocardiograms; PASI, Psoriasis Area and Severity Index; sPGA, static Physician's Global Assessment; VAS, visual analogue scale

Signature of the researcher: ____________   Date: ____________________
