# Supplementary material for: Add-on effects of Chinese herbal medicine external application (FZHFZY) to topical urea for mild-to-moderate psoriasis vulgaris: Protocol for a double-blinded randomized controlled pilot trial embedded with a qualitative study
Source: PLoS One. 2024 Mar 21;19(3):e0297834. doi: 10.1371/journal.pone.0297834 (PMC10956750; doi:10.1371/journal.pone.0297834)
Supplement: S8 File — (PDF) [file pone.0297834.s009.pdf]

S8 File. Patient-reported outcomes form

Add-on effects of Chinese herbal medicine (CHM) external application (FZHFZY) for mild-to-moderate psoriasis vulgaris: a pilot randomised placebo-controlled trial

Patient Report Outcomes Forms

Serial number of the enrolment ： |__|__|__|

Randomisation code ： |__|__|__|

Abbrevation of the patient's name ： |__|__|__|__|

Researcher ： ____________________

Guangdong Provincial Hospital of Chinese Medicine

Table of Contents

Baseline (Week 0) 1
Dermatology Life Quality Index 1
Itch Visual Analogue Scale 2
Skindex-16 Instrument 3
Week 2 4
Itch Visual Analogue Scale 4
Week 4 5
Itch Visual Analogue Scale 5
Blinding credibility 6
Week 6 7
Itch Visual Analogue Scale 7
Week 8 8
Dermatology Life Quality Index 8
Itch Visual Analogue Scale 9
Acceptability of the trial 10
Skindex-16 Instrument 11
Blinding credibility 12
Week 12 13
Itch Visual Analogue Scale 13
Week 16 14
Itch Visual Analogue Scale 14
Week 20 15
Dermatology Life Quality Index 15
Itch Visual Analogue Scale 16
Acceptability of the trial 17
Skindex-16 Instrument 18
Blinding credibility 19

Baseline (Week 0)

Dermatology Life Quality Index

The aim of this questionnaire is to measure how much your skin problem has affected your life OVER THE LAST WEEK. Please tick ☑ one box for each question.

1. Over the last week, how **itchy**, **sore**, **painful** or **stinging** has your skin been?
☐ Very much ☐ A lot ☐ A little ☐ Not at all
2. Over the last week, how **embarrassed** or **self-conscious** have you been because of your skin?
☐ Very much ☐ A lot ☐ A little ☐ Not at all
3. Over the last week, how much has your skin interfered with you going **shopping** or looking after your **home** or **garden**?
☐ Very much ☐ A lot ☐ A little ☐ Not at all ☐ Not relevant
4. Over the last week, how much has your skin influenced the **clothes** you wear?
☐ Very much ☐ A lot ☐ A little ☐ Not at all ☐ Not relevant
5. Over the last week, how much has your skin affected any **social** or **leisure** activities?
☐ Very much ☐ A lot ☐ A little ☐ Not at all ☐ Not relevant
6. Over the last week, how much has your skin made it difficult for you to do any **sport**?
☐ Very much ☐ A lot ☐ A little ☐ Not at all ☐ Not relevant
7. Over the last week, has your skin prevented you from **working** or **studying**?
☐ Yes ☐ No ☐ Not relevant
If "No", over the last week how much has your skin been a problem at **work** or **studying**?
☐ A lot ☐ A little ☐ Not at all
8. Over the last week, how much has your skin created problems with your **partner** or any of your **close friends** or **relatives**?
☐ Very much ☐ A lot ☐ A little ☐ Not at all ☐ Not relevant
9. Over the last week, how much has your skin caused any **sexual difficulties**?
☐ Very much ☐ A lot ☐ A little ☐ Not at all ☐ Not relevant
10. Over the last week, how much of a problem has the **treatment** for your skin been, for example by making your home messy, or by taking up time?
☐ Very much ☐ A lot ☐ A little ☐ Not at all ☐ Not relevant

Please check you have answered EVERY question. Thank you.

©AY Finlay, GK Khan, April 1992. www.dermatology.org.uk. This must not be copied without the permission of the authors.

Signature of the patient: _______________ Date: ________________

Baseline (Week 0)

Itch Visual Analogue Scale

On a scale of "no itch" (left) to "the worst possible itch" (right), how was
Please mark a position between 0 and 10 that best represents your itch with a cross (×) on the line below:
e.g. "0 (no itch) ——×—— 10 (the worst possible itch)"

your itch, on average, in the past 24 hours?

0 (no itch) ———————————————— 10 (the worst possible itch)

your worst itch in the past 24 hours?

0 (no itch) ———————————————— 10 (the worst possible itch)

Validated scales according to IFSI SIG/EADV Task Force Pruritus.

Phan NQ, Blome C, Fritz F, Gerss J, Reich A, Ebata T, Augustin M, Szepietowski JC, Ständer S. Assessment of pruritus intensity: prospective study on validity and reliability of the visual analogue scale, numerical rating scale and verbal rating scale in 471 patients with chronic pruritus. Acta Derm Venereol. 2012 Sep;92(5):502-7.

Verweyen E, Ständer S, Kreitz K, Höben I, Osada N, Gernart M, Riepe C, Pereira M, Blome C, Augustin M, Zeidler C. Validation of a Comprehensive Set of Pruritus Assessment Instruments: The Chronic Pruritus Tools Questionnaire PRURITOOLS. Acta Derm Venereol. 2019 Jun 1;99(7):657-663.

Signature of the patient: _______________ Date: ________________

Baseline (Week 0)

Skindex-16 Instrument

THESE QUESTIONS CONCERN THE SKIN CONDITION WHICH HAS BOTHERED YOU THE MOST DURING THE PAST WEEK.

	**During the past week, how often have you been bothered by:**	Never Bothered ↓			•			Always Bothered ↓
1.	Your skin condition **itching**	☐0	☐1	☐2	☐3	☐4	☐5	☐6
2.	Your skin condition **burning** or **stinging**	☐0	☐1	☐2	☐3	☐4	☐5	☐6
3.	Your skin condition **hurting**	☐0	☐1	☐2	☐3	☐4	☐5	☐6
4.	Your skin condition **being irritated**	☐0	☐1	☐2	☐3	☐4	☐5	☐6
5.	The **persistence/reoccurrence** of your skin condition	☐0	☐1	☐2	☐3	☐4	☐5	☐6
6.	**Worry** about your skin condition (For example: that it will spread, get worse, scar, be unpredictable, etc)	☐0	☐1	☐2	☐3	☐4	☐5	☐6
7.	The **appearance** of your skin condition	☐0	☐1	☐2	☐3	☐4	☐5	☐6
8.	**Frustration** about your skin condition	☐0	☐1	☐2	☐3	☐4	☐5	☐6
9.	**Embarrassment** about your skin condition	☐0	☐1	☐2	☐3	☐4	☐5	☐6
10.	**Being annoyed** about your skin condition	☐0	☐1	☐2	☐3	☐4	☐5	☐6
11.	**Feeling depressed** about your skin condition	☐0	☐1	☐2	☐3	☐4	☐5	☐6
12.	The effects of your skin condition on your **interactions with others** (For example: interactions with family, friends, close relationships, etc)	☐0	☐1	☐2	☐3	☐4	☐5	☐6
13.	The effects of your skin condition on your **desire to be with people**	☐0	☐1	☐2	☐3	☐4	☐5	☐6
14.	Your skin condition making it hard to **show affection**	☐0	☐1	☐2	☐3	☐4	☐5	☐6
15.	The effects of your skin condition on your **daily activities**	☐0	☐1	☐2	☐3	☐4	☐5	☐6
16.	Your skin condition making it hard to **work or do what you enjoy**	☐0	☐1	☐2	☐3	☐4	☐5	☐6

He, Z., et al., Development and psychometric validation of the Chinese version of Skindex-29 and Skindex-16. Health Qual Life Outcomes, 2014. 12: p. 190.

Signature of the patient: ________________ Date: __________________

Week 2

Itch Visual Analogue Scale

On a scale of "no itch" (left) to "the worst possible itch" (right), how was
Please mark a position between 0 and 10 that best represents your itch with a cross (×) on the line below:
e.g. "0 (no itch) ——×—— 10 (the worst possible itch)"

your itch, on average, in the past 24 hours?

0 (no itch) ________________________________ 10 (the worst possible itch)

your worst itch in the past 24 hours?

0 (no itch) ________________________________ 10 (the worst possible itch)

Validated scales according to IFSI SIG/EADV Task Force Pruritus.

Phan NQ, Blome C, Fritz F, Gerss J, Reich A, Ebata T, Augustin M, Szepietowski JC, Ständer S. Assessment of pruritus intensity: prospective study on validity and reliability of the visual analogue scale, numerical rating scale and verbal rating scale in 471 patients with chronic pruritus. Acta Derm Venereol. 2012 Sep;92(5):502-7.

Verweyen E, Ständer S, Kreitz K, Höben I, Osada N, Gernart M, Riepe C, Pereira M, Blome C, Augustin M, Zeidler C. Validation of a Comprehensive Set of Pruritus Assessment Instruments: The Chronic Pruritus Tools Questionnaire PRURITOOLS. Acta Derm Venereol. 2019 Jun 1;99(7):657-663.

Signature of the patient: _______________ Date: _________________

Week 4

Itch Visual Analogue Scale

On a scale of "no itch" (left) to "the worst possible itch" (right), how was
Please mark a position between 0 and 10 that best represents your itch with a cross (×) on the line below:
e.g. "0 (no itch) ———×——— 10 (the worst possible itch)"

your itch, on average, in the past 24 hours?

0 (no itch) ________________________________ 10 (the worst possible itch)

your worst itch in the past 24 hours?

0 (no itch) ________________________________ 10 (the worst possible itch)

Validated scales according to IFSI SIG/EADV Task Force Pruritus.

Phan NQ, Blome C, Fritz F, Gerss J, Reich A, Ebata T, Augustin M, Szepietowski JC, Ständer S. Assessment of pruritus intensity: prospective study on validity and reliability of the visual analogue scale, numerical rating scale and verbal rating scale in 471 patients with chronic pruritus. Acta Derm Venereol. 2012 Sep;92(5):502-7.

Verweyen E, Ständer S, Kreitz K, Höben I, Osada N, Gernart M, Riepe C, Pereira M, Blome C, Augustin M, Zeidler C. Validation of a Comprehensive Set of Pruritus Assessment Instruments: The Chronic Pruritus Tools Questionnaire PRURITOOLS. Acta Derm Venereol. 2019 Jun 1;99(7):657-663.

Signature of the patient: ______________ Date: ________________

Week 4

Blinding credibility

Do you think the treatment you received is: ☐ CHM ☐ Placebo ☐ Unsure

Signature of the patient: ________________ Date: _________________

Week 6

Itch Visual Analogue Scale

On a scale of "no itch" (left) to "the worst possible itch" (right), how was

Please mark a position between 0 and 10 that best represents your itch with a cross (×) on the line below:

e.g. "0 (no itch) ———×——— 10 (the worst possible itch)"

your itch, on average, in the past 24 hours?

0 (no itch) ____ 10 (the worst possible itch)

your worst itch in the past 24 hours?

0 (no itch) ____ 10 (the worst possible itch)

Validated scales according to IFSI SIG/EADV Task Force Pruritus.

Phan NQ, Blome C, Fritz F, Gerss J, Reich A, Ebata T, Augustin M, Szepietowski JC, Ständer S. Assessment of pruritus intensity: prospective study on validity and reliability of the visual analogue scale, numerical rating scale and verbal rating scale in 471 patients with chronic pruritus. Acta Derm Venereol. 2012 Sep;92(5):502-7.

Verweyen E, Ständer S, Kreitz K, Höben I, Osada N, Gernart M, Riepe C, Pereira M, Blome C, Augustin M, Zeidler C. Validation of a Comprehensive Set of Pruritus Assessment Instruments: The Chronic Pruritus Tools Questionnaire PRURITOOLS. Acta Derm Venereol. 2019 Jun 1;99(7):657-663.

Signature of the patient: _______________ Date: ________________

Week 8

Dermatology Life Quality Index

The aim of this questionnaire is to measure how much your skin problem has affected your life OVER THE LAST WEEK. Please tick ☑ one box for each question.

1. Over the last week, how **itchy**, **sore**, **painful** or **stinging** has your skin been?
☐ Very much ☐ A lot ☐ A little ☐ Not at all
2. Over the last week, how **embarrassed** or **self-conscious** have you been because of your skin?
☐ Very much ☐ A lot ☐ A little ☐ Not at all
3. Over the last week, how much has your skin interfered with you going **shopping** or looking after your **home** or **garden**?
☐ Very much ☐ A lot ☐ A little ☐ Not at all ☐ Not relevant
4. Over the last week, how much has your skin influenced the **clothes** you wear?
☐ Very much ☐ A lot ☐ A little ☐ Not at all ☐ Not relevant
5. Over the last week, how much has your skin affected any **social** or **leisure** activities?
☐ Very much ☐ A lot ☐ A little ☐ Not at all ☐ Not relevant
6. Over the last week, how much has your skin made it difficult for you to do any **sport**?
☐ Very much ☐ A lot ☐ A little ☐ Not at all ☐ Not relevant
7. Over the last week, has your skin prevented you from **working** or **studying**?
☐ Yes ☐ No ☐ Not relevant
If "No", over the last week how much has your skin been a problem at **work** or **studying**?
☐ A lot ☐ A little ☐ Not at all
8. Over the last week, how much has your skin created problems with your **partner** or any of your **close friends** or **relatives**?
☐ Very much ☐ A lot ☐ A little ☐ Not at all ☐ Not relevant
9. Over the last week, how much has your skin caused any **sexual difficulties**?
☐ Very much ☐ A lot ☐ A little ☐ Not at all ☐ Not relevant
10. Over the last week, how much of a problem has the **treatment** for your skin been, for example by making your home messy, or by taking up time?
☐ Very much ☐ A lot ☐ A little ☐ Not at all ☐ Not relevant

Please check you have answered EVERY question. Thank you.

©AY Finlay, GK Khan, April 1992. www.dermatology.org.uk. This must not be copied without the permission of the authors.

Signature of the patient: ________________ Date: _________________

Week 8

Itch Visual Analogue Scale

On a scale of "no itch" (left) to "the worst possible itch" (right), how was
Please mark a position between 0 and 10 that best represents your itch with a cross (×) on the line below:
e.g. "0 (no itch) ——×—— 10 (the worst possible itch)"

your itch, on average, in the past 24 hours?

0 (no itch) ________________________________ 10 (the worst possible itch)

your worst itch in the past 24 hours?

0 (no itch) ________________________________ 10 (the worst possible itch)

Validated scales according to IFSI SIG/EADV Task Force Pruritus.

Phan NQ, Blome C, Fritz F, Gerss J, Reich A, Ebata T, Augustin M, Szepietowski JC, Ständer S. Assessment of pruritus intensity: prospective study on validity and reliability of the visual analogue scale, numerical rating scale and verbal rating scale in 471 patients with chronic pruritus. Acta Derm Venereol. 2012 Sep;92(5):502-7.

Verweyen E, Ständer S, Kreitz K, Höben I, Osada N, Gernart M, Riepe C, Pereira M, Blome C, Augustin M, Zeidler C. Validation of a Comprehensive Set of Pruritus Assessment Instruments: The Chronic Pruritus Tools Questionnaire PRURITOOLS. Acta Derm Venereol. 2019 Jun 1;99(7):657-663.

Signature of the patient: _______________ Date: ________________

Week 8

Acceptability of the trial

How helpful was this trial? Please mark a position between 0 and 10 that best represents your overall satisfaction with a circle on the scale below.

E.g.

1	2	3	4	5	6	7	8	9	10

0	1	2	3	4	5	6	7	8	9	10
No satisfied			Could be better		Satisfied		Exception ally satisfied			The most satisfied

If you are unsatisfied with the trial, please give details of the reason: ______________________

____

____

____

Kleiss, I., et al., A Comparison of 4 Single-Question Measures of Patient Satisfaction. Journal of clinical outcomes management: JCOM, 2020. 27: p. 41-48.

Signature of the patient: ______________ Date: ________________

Week 8

Skindex-16 Instrument

THESE QUESTIONS CONCERN THE SKIN CONDITION WHICH HAS BOTHERED YOU THE MOST DURING THE PAST WEEK.

**During the past week, how often have you been bothered by:**		Never Bothered ↓			•			Always Bothered ↓
1.	Your skin condition **itching**	☐0	☐1	☐2	☐3	☐4	☐5	☐6
2.	Your skin condition **burning** or **stinging**	☐0	☐1	☐2	☐3	☐4	☐5	☐6
3.	Your skin condition **hurting**	☐0	☐1	☐2	☐3	☐4	☐5	☐6
4.	Your skin condition **being irritated**	☐0	☐1	☐2	☐3	☐4	☐5	☐6
5.	The **persistence/reoccurrence** of your skin condition	☐0	☐1	☐2	☐3	☐4	☐5	☐6
6.	**Worry** about your skin condition (For example: that it will spread, get worse, scar, be unpredictable, etc)	☐0	☐1	☐2	☐3	☐4	☐5	☐6
7.	The **appearance** of your skin condition	☐0	☐1	☐2	☐3	☐4	☐5	☐6
8.	**Frustration** about your skin condition	☐0	☐1	☐2	☐3	☐4	☐5	☐6
9.	**Embarrassment** about your skin condition	☐0	☐1	☐2	☐3	☐4	☐5	☐6
10.	**Being annoyed** about your skin condition	☐0	☐1	☐2	☐3	☐4	☐5	☐6
11.	**Feeling depressed** about your skin condition	☐0	☐1	☐2	☐3	☐4	☐5	☐6
12.	The effects of your skin condition on your **interactions with others** (For example: interactions with family, friends, close relationships, etc)	☐0	☐1	☐2	☐3	☐4	☐5	☐6
13.	The effects of your skin condition on your **desire to be with people**	☐0	☐1	☐2	☐3	☐4	☐5	☐6
14.	Your skin condition making it hard to **show affection**	☐0	☐1	☐2	☐3	☐4	☐5	☐6
15.	The effects of your skin condition on your **daily activities**	☐0	☐1	☐2	☐3	☐4	☐5	☐6
16.	Your skin condition making it hard to **work or do what you enjoy**	☐0	☐1	☐2	☐3	☐4	☐5	☐6

He, Z., et al., Development and psychometric validation of the Chinese version of Skindex-29 and Skindex-16. Health Qual Life Outcomes, 2014. 12: p. 190.

Signature of the patient: ________________ Date: __________________

Week 8

Blinding credibility

Do you think the treatment you received is: ☐ CHM ☐ Placebo ☐ Unsure

Signature of the patient: ________________ Date: ________________

Week 12

Itch Visual Analogue Scale

On a scale of "no itch" (left) to "the worst possible itch" (right), how was

Please mark a position between 0 and 10 that best represents your itch with a cross (×) on the line below:

e.g. "0 (no itch) ——×—— 10 (the worst possible itch)"

your itch, on average, in the past 24 hours?

0 (no itch) ________________________________ 10 (the worst possible itch)

your worst itch in the past 24 hours?

0 (no itch) ________________________________ 10 (the worst possible itch)

Validated scales according to IFSI SIG/EADV Task Force Pruritus.

Phan NQ, Blome C, Fritz F, Gerss J, Reich A, Ebata T, Augustin M, Szepietowski JC, Ständer S. Assessment of pruritus intensity: prospective study on validity and reliability of the visual analogue scale, numerical rating scale and verbal rating scale in 471 patients with chronic pruritus. Acta Derm Venereol. 2012 Sep;92(5):502-7.

Verweyen E, Ständer S, Kreitz K, Höben I, Osada N, Gernart M, Riepe C, Pereira M, Blome C, Augustin M, Zeidler C. Validation of a Comprehensive Set of Pruritus Assessment Instruments: The Chronic Pruritus Tools Questionnaire PRURITOOLS. Acta Derm Venereol. 2019 Jun 1;99(7):657-663.

Signature of the patient: _______________ Date: ________________

Week 16

Itch Visual Analogue Scale

On a scale of "no itch" (left) to "the worst possible itch" (right), how was
Please mark a position between 0 and 10 that best represents your itch with a cross (×) on the line below:
e.g. "0 (no itch) ——×—— 10 (the worst possible itch)"

your itch, on average, in the past 24 hours?

0 (no itch) ———————————————— 10 (the worst possible itch)

your worst itch in the past 24 hours?

0 (no itch) ———————————————— 10 (the worst possible itch)

Validated scales according to IFSI SIG/EADV Task Force Pruritus.

Phan NQ, Blome C, Fritz F, Gerss J, Reich A, Ebata T, Augustin M, Szepietowski JC, Ständer S. Assessment of pruritus intensity: prospective study on validity and reliability of the visual analogue scale, numerical rating scale and verbal rating scale in 471 patients with chronic pruritus. Acta Derm Venereol. 2012 Sep;92(5):502-7.

Verweyen E, Ständer S, Kreitz K, Höben I, Osada N, Gernart M, Riepe C, Pereira M, Blome C, Augustin M, Zeidler C. Validation of a Comprehensive Set of Pruritus Assessment Instruments: The Chronic Pruritus Tools Questionnaire PRURITOOLS. Acta Derm Venereol. 2019 Jun 1;99(7):657-663.

Signature of the patient: _______________ Date: _________________

Week 20

Dermatology Life Quality Index

The aim of this questionnaire is to measure how much your skin problem has affected your life OVER THE LAST WEEK. Please tick ☑ one box for each question.

1. Over the last week, how **itchy**, **sore**, **painful** or **stinging** has your skin been?
☐ Very much ☐ A lot ☐ A little ☐ Not at all

2. Over the last week, how **embarrassed** or **self-conscious** have you been because of your skin?
☐ Very much ☐ A lot ☐ A little ☐ Not at all

3. Over the last week, how much has your skin interfered with you going **shopping** or looking after your **home** or **garden**?
☐ Very much ☐ A lot ☐ A little ☐ Not at all ☐ Not relevant

4. Over the last week, how much has your skin influenced the **clothes** you wear?
☐ Very much ☐ A lot ☐ A little ☐ Not at all ☐ Not relevant

5. Over the last week, how much has your skin affected any **social** or **leisure** activities?
☐ Very much ☐ A lot ☐ A little ☐ Not at all ☐ Not relevant

6. Over the last week, how much has your skin made it difficult for you to do any **sport**?
☐ Very much ☐ A lot ☐ A little ☐ Not at all ☐ Not relevant

7. Over the last week, has your skin prevented you from **working** or **studying**?
☐ Yes ☐ No ☐ Not relevant

If "No", over the last week how much has your skin been a problem at **work** or **studying**?
☐ A lot ☐ A little ☐ Not at all

8. Over the last week, how much has your skin created problems with your **partner** or any of your **close friends** or **relatives**?
☐ Very much ☐ A lot ☐ A little ☐ Not at all ☐ Not relevant

9. Over the last week, how much has your skin caused any **sexual difficulties**?
☐ Very much ☐ A lot ☐ A little ☐ Not at all ☐ Not relevant

10. Over the last week, how much of a problem has the **treatment** for your skin been, for example by making your home messy, or by taking up time?
☐ Very much ☐ A lot ☐ A little ☐ Not at all ☐ Not relevant

Please check you have answered EVERY question. Thank you.

©AY Finlay, GK Khan, April 1992. www.dermatology.org.uk. This must not be copied without the permission of the authors.

Signature of the patient: _______________ Date: ________________

Week 20

Itch Visual Analogue Scale

On a scale of "no itch" (left) to "the worst possible itch" (right), how was
Please mark a position between 0 and 10 that best represents your itch with a cross (×) on the line below:
e.g. "0 (no itch) ——×—— 10 (the worst possible itch)"

your itch, on average, in the past 24 hours?

0 (no itch) ____ 10 (the worst possible itch)

your worst itch in the past 24 hours?

0 (no itch) ____ 10 (the worst possible itch)

Validated scales according to IFSI SIG/EADV Task Force Pruritus.

Phan NQ, Blome C, Fritz F, Gerss J, Reich A, Ebata T, Augustin M, Szepietowski JC, Ständer S. Assessment of pruritus intensity: prospective study on validity and reliability of the visual analogue scale, numerical rating scale and verbal rating scale in 471 patients with chronic pruritus. Acta Derm Venereol. 2012 Sep;92(5):502-7.

Verweyen E, Ständer S, Kreitz K, Höben I, Osada N, Gernart M, Riepe C, Pereira M, Blome C, Augustin M, Zeidler C. Validation of a Comprehensive Set of Pruritus Assessment Instruments: The Chronic Pruritus Tools Questionnaire PRURITOOLS. Acta Derm Venereol. 2019 Jun 1;99(7):657-663.

Signature of the patient: _______________ Date: _________________

Week 20

Acceptability of the trial

How helpful was this trial? Please mark a position between 0 and 10 that best represents your overall satisfaction with a circle on the scale below.

E.g.

1	2	3	4	(5)	6	7	8	9	10

0	1	2	3	4	5	6	7	8	9	10
No satisfied			Could be better		Satisfied		Exception ally satisfied			The most satisfied

If you are unsatisfied with the trial, please give details of the reason: ______________________

____

____

____

Kleiss, I., et al., A Comparison of 4 Single-Question Measures of Patient Satisfaction. Journal of clinical outcomes management: JCOM, 2020. 27: p. 41-48.

Signature of the patient: _______________ Date: ________________

Week 20

Skindex-16 Instrument

THESE QUESTIONS CONCERN THE SKIN CONDITION WHICH HAS BOTHERED YOU THE MOST DURING THE PAST WEEK.

**During the past week, how often have you been bothered by:**		Never Bothered ↓			•			Always Bothered ↓
1.	Your skin condition **itching**	☐0	☐1	☐2	☐3	☐4	☐5	☐6
2.	Your skin condition **burning** or **stinging**	☐0	☐1	☐2	☐3	☐4	☐5	☐6
3.	Your skin condition **hurting**	☐0	☐1	☐2	☐3	☐4	☐5	☐6
4.	Your skin condition **being irritated**	☐0	☐1	☐2	☐3	☐4	☐5	☐6
5.	The **persistence/reoccurrence** of your skin condition	☐0	☐1	☐2	☐3	☐4	☐5	☐6
6.	**Worry** about your skin condition (For example: that it will spread, get worse, scar, be unpredictable, etc)	☐0	☐1	☐2	☐3	☐4	☐5	☐6
7.	The **appearance** of your skin condition	☐0	☐1	☐2	☐3	☐4	☐5	☐6
8.	**Frustration** about your skin condition	☐0	☐1	☐2	☐3	☐4	☐5	☐6
9.	**Embarrassment** about your skin condition	☐0	☐1	☐2	☐3	☐4	☐5	☐6
10.	**Being annoyed** about your skin condition	☐0	☐1	☐2	☐3	☐4	☐5	☐6
11.	**Feeling depressed** about your skin condition	☐0	☐1	☐2	☐3	☐4	☐5	☐6
12.	The effects of your skin condition on your **interactions with others** (For example: interactions with family, friends, close relationships, etc)	☐0	☐1	☐2	☐3	☐4	☐5	☐6
13.	The effects of your skin condition on your **desire to be with people**	☐0	☐1	☐2	☐3	☐4	☐5	☐6
14.	Your skin condition making it hard to **show affection**	☐0	☐1	☐2	☐3	☐4	☐5	☐6
15.	The effects of your skin condition on your **daily activities**	☐0	☐1	☐2	☐3	☐4	☐5	☐6
16.	Your skin condition making it hard to **work or do what you enjoy**	☐0	☐1	☐2	☐3	☐4	☐5	☐6

He, Z., et al., Development and psychometric validation of the Chinese version of Skindex-29 and Skindex-16. Health Qual Life Outcomes, 2014. 12: p. 190.

Signature of the patient: ________________ Date: ________________

Week 20

Blinding credibility

Do you think the treatment you received is: ☐ CHM ☐ Placebo ☐ Unsure

Signature of the patient: ________________ Date: __________________
