# Supplementary material for: Add-on effects of Chinese herbal medicine external application (FZHFZY) to topical urea for mild-to-moderate psoriasis vulgaris: Protocol for a double-blinded randomized controlled pilot trial embedded with a qualitative study
Source: PLoS One. 2024 Mar 21;19(3):e0297834. doi: 10.1371/journal.pone.0297834 (PMC10956750; doi:10.1371/journal.pone.0297834)
Supplement: S9 File — (PDF) [file pone.0297834.s010.pdf]

S9 File. Patient home diary during the follow-up period

Add-on Chinese herbal medicine (CHM) external application (FZHFZY) for mild-to-moderate psoriasis vulgaris: a pilot randomised placebo-controlled trial

Patient Home Diary (the follow-up period)

Serial number of the enrolment : |__|__|__|

Randomisation code : |__|__|__|

Abbreviation of the patient’s name : |__|__|__|__|

The researcher : ________________________

Guangdong Provincial Hospital of Chinese Medicine

This page is filled in by the researcher.

Patient home diary completing guidelines:

1. Please record the home diary from the date of using trial medications.
2. Please record any symptoms during the follow-up period. If there are severe conditions, please contact with the researcher immediately.
3. The home diary of the patient with psoriasis vulgairs is kept by the patients. **Please bring it back to the hospital during each visit.**
4. Please apply these instructions in your daily life during the trial:
   - Have a good rest, avoid cold, avoid suffering from inflammatory diseases such as acute pharyngitis and tonsillitis
   - Avoid alcohol, ensure enough sleep, and maintain good mood
   - Keep the skin clean and avoid bathing in hot water.

The follow-up period

	Week 1							Week 2						
Date/month														
Did you take any treatment for psoriasis?														
If you took any treatment for psoriasis, please record the name and dosage of the treatment.														
Did you have any other symptoms?														
If you had other symptoms, did you take any treatment? Please specify.														
Psoriasis symptoms aggravation or improvement?	(Record date and details)													
Psoriasis symptoms aggravation or improvement?	(Potential factors)													

- If the date is "1st February", please indicate "1/2" in the table. Please record medication information for two weeks in the form. If you cannot return to the hospital on the date as the appointment, you can return to the hospital within 3 days before and after the appointment.
- If you took any treatment for psoriasis, please record the name and dosage of the treatment, as well as put a " √ " in the corresponding box; if not, put a "×" in the corresponding box.
- If you had any other symptoms than psoriasis, please put a " √ " in the corresponding box, and record your treatment if there is any. Otherwise put a "×" in the box.
- If other diseases require long-term treatment, please fill in the "Concomitant medication form" on the back.
- If your psoriasis symptoms worsened/improved, record the date of occurrence, details, and possible influencing factors in the corresponding box of the table.

Concomitant medication form

Trade name or generic name	Usage daily	Disease	Start date	End date	Ongoing

- If the medication is still in use at the visit, the "end date" box is not required to be filled in, please put a " √ " in the "ongoing" box. If the medication has been discontinued at the visit, please provide the "end date" and put a "×" in the "ongoing" box.

The follow-up period

	Week 3							Week 4						
Date/month	/	/	/	/	/	/	/	/	/	/	/	/	/	/
Did you take any treatment for psoriasis?														
If you took any treatment for psoriasis, please record the name and dosage of the treatment.														
Did you have any other symptoms?														
If you had other symptoms, did you take any treatment? Please specify.														
Psoriasis symptoms aggravation or improvement?	(Record date and details)													
Psoriasis symptoms aggravation or improvement?	(Potential factors)													

- If the date is "1st February", please indicate "1/2" in the table. Please record medication information for two weeks in the form. If you cannot return to the hospital on the date as the appointment, you can return to the hospital within 3 days before and after the appointment.
- If you took any treatment for psoriasis, please record the name and dosage of the treatment, as well as put a " √ " in the corresponding box; if not, put a "×" in the corresponding box.
- If you had any other symptoms than psoriasis, please put a " √ " in the corresponding box, and record your treatment if there is any. Otherwise put a "×" in the box.
- If other diseases require long-term treatment, please fill in the "Concomitant medication form" on the back.
- If your psoriasis symptoms worsened/improved, record the date of occurrence, details, and possible influencing factors in the corresponding box of the table.

Concomitant medication form

Trade name or generic name	Usage daily	Disease	Start date	End date	Ongoing

- If the medication is still in use at the visit, the "end date" box is not required to be filled in, please put a "√" in the "ongoing" box. If the medication has been discontinued at the visit, please provide the "end date" and put a "×" in the "ongoing" box.

The follow-up period

	Week 5							Week 6						
Date/month	/	/	/	/	/	/	/	/	/	/	/	/	/	/
Did you take any treatment for psoriasis?														
If you took any treatment for psoriasis, please record the name and dosage of the treatment.														
Did you have any other symptoms?														
If you had other symptoms, did you take any treatment? Please specify.														
Psoriasis symptoms aggravation or improvement?	(Record date and details)													
Psoriasis symptoms aggravation or improvement?	(Potential factors)													

- If the date is "1st February", please indicate "1/2" in the table. Please record medication information for two weeks in the form. If you cannot return to the hospital on the date as the appointment, you can return to the hospital within 3 days before and after the appointment.
- If you took any treatment for psoriasis, please record the name and dosage of the treatment, as well as put a " √ " in the corresponding box; if not, put a "×" in the corresponding box.
- If you had any other symptoms than psoriasis, please put a " √ " in the corresponding box, and record your treatment if there is any. Otherwise put a "×" in the box.
- If other diseases require long-term treatment, please fill in the "Concomitant medication form" on the back.
- If your psoriasis symptoms worsened/improved, record the date of occurrence, details, and possible influencing factors in the corresponding box of the table.

Abbreviation of the patient's name |__|__|__|__| Serial number of the enrolment |__|__|__|

Concomitant medication form

Trade name or generic name	Usage daily	Disease	Start date	End date	Ongoing

- If the medication is still in use at the visit, the "end date" box is not required to be filled in, please put a "√" in the "ongoing" box. If the medication has been discontinued at the visit, please provide the "end date" and put a "×" in the "ongoing" box.

Header is filled by the researcher.

The follow-up period

	Week 7							Week 8						
Date/month														
Did you take any treatment for psoriasis?														
If you took any treatment for psoriasis, please record the name and dosage of the treatment.														
Did you have any other symptoms?														
If you had other symptoms, did you take any treatment? Please specify.														
Psoriasis symptoms aggravation or improvement?	(Record date and details)													
Psoriasis symptoms aggravation or improvement?	(Potential factors)													

- If the date is "1st February", please indicate "1/2" in the table. Please record medication information for two weeks in the form. If you cannot return to the hospital on the date as the appointment, you can return to the hospital within 3 days before and after the appointment.
- If you took any treatment for psoriasis, please record the name and dosage of the treatment, as well as put a " √ " in the corresponding box; if not, put a "×" in the corresponding box.
- If you had any other symptoms than psoriasis, please put a " √ " in the corresponding box, and record your treatment if there is any. Otherwise put a "×" in the box.
- If other diseases require long-term treatment, please fill in the "Concomitant medication form" on the back.
- If your psoriasis symptoms worsened/improved, record the date of occurrence, details, and possible influencing factors in the corresponding box of the table.

Concomitant medication form

Trade name or generic name	Usage daily	Disease	Start date	End date	Ongoing

- If the medication is still in use at the visit, the "end date" box is not required to be filled in, please put a " √ " in the "ongoing" box. If the medication has been discontinued at the visit, please provide the "end date" and put a "×" in the "ongoing" box.

Abbreviation of the patient’s name |__|__|__|__| Serial number of the enrolment |__|__|__|

The follow-up period

	**Week 9**							**Week 10**						
Date/month	/	/	/	/	/	/	/	/	/	/	/	/	/	/
Did you take any treatment for psoriasis?														
If you took any treatment for psoriasis, please record the name and dosage of the treatment.														
Did you have any other symptoms?														
If you had other symptoms, did you take any treatment? Please specify.														
Psoriasis symptoms aggravation or improvement?	(Record date and details)													
Psoriasis symptoms aggravation or improvement?	(Potential factors)													

- If the date is "1st February", please indicate "1/2" in the table. Please record medication information for two weeks in the form. If you cannot return to the hospital on the date as the appointment, you can return to the hospital within 3 days before and after the appointment.
- If you took any treatment for psoriasis, please record the name and dosage of the treatment, as well as put a " √ " in the corresponding box; if not, put a "×" in the corresponding box.
- If you had any other symptoms than psoriasis, please put a " √ " in the corresponding box, and record your treatment if there is any. Otherwise put a "×" in the box.
- If other diseases require long-term treatment, please fill in the "Concomitant medication form" on the back.
- If your psoriasis symptoms worsened/improved, record the date of occurrence, details, and possible influencing factors in the corresponding box of the table.

Abbreviation of the patient's name |__|__|__|__| Serial number of the enrolment |__|__|__|

Concomitant medication form

Trade name or generic name	Usage daily	Disease	Start date	End date	Ongoing

- If the medication is still in use at the visit, the "end date" box is not required to be filled in, please put a " √ " in the "ongoing" box. If the medication has been discontinued at the visit, please provide the "end date" and put a "×" in the "ongoing" box.

Header is filled by the researcher.

Abbreviation of the patient’s name |__|__|__|__| Serial number of the enrolment |__|__|__|

The follow-up period

	Week 11							Week 12						
Date/month	/	/	/	/	/	/	/	/	/	/	/	/	/	/
Did you take any treatment for psoriasis?														
If you took any treatment for psoriasis, please record the name and dosage of the treatment.														
Did you have any other symptoms?														
If you had other symptoms, did you take any treatment? Please specify.														
Psoriasis symptoms aggravation or improvement?	(Record date and details)													
Psoriasis symptoms aggravation or improvement?	(Potential factors)													

- If the date is "1st February", please indicate "1/2" in the table. Please record medication information for two weeks in the form. If you cannot return to the hospital on the date as the appointment, you can return to the hospital within 3 days before and after the appointment.
- If you took any treatment for psoriasis, please record the name and dosage of the treatment, as well as put a " √ " in the corresponding box; if not, put a "×" in the corresponding box.
- If you had any other symptoms than psoriasis, please put a " √ " in the corresponding box, and record your treatment if there is any. Otherwise put a "×" in the box.
- If other diseases require long-term treatment, please fill in the "Concomitant medication form" on the back.
- If your psoriasis symptoms worsened/improved, record the date of occurrence, details, and possible influencing factors in the corresponding box of the table.

Abbreviation of the patient's name |__|__|__|__| Serial number of the enrolment |__|__|__|

Concomitant medication form

Trade name or generic name	Usage daily	Disease	Start date	End date	Ongoing

- If the medication is still in use at the visit, the "end date" box is not required to be filled in, please put a "√" in the "ongoing" box. If the medication has been discontinued at the visit, please provide the "end date" and put a "×" in the "ongoing" box.
