# Supplementary material for: Add-on effects of Chinese herbal medicine external application (FZHFZY) to topical urea for mild-to-moderate psoriasis vulgaris: Protocol for a double-blinded randomized controlled pilot trial embedded with a qualitative study
Source: PLoS One. 2024 Mar 21;19(3):e0297834. doi: 10.1371/journal.pone.0297834 (PMC10956750; doi:10.1371/journal.pone.0297834)
Supplement: S10 File — (DOCX) [file pone.0297834.s011.docx]

**S10 File. Informed consent of the qualitative study**

**Dear sir/ Madam,**

You are diagnosed with psoriasis vulgaris.

You are invited to take part in this research project entitled “Experience and acceptability of add-on Chinese herbal medicine (CHM) external application (FZHFZY) for mild-to-moderate psoriasis vulgaris: a qualitative study”. The aim of the study is to qualitatively evaluate the feasibility of the protocol of a randomised controlled trial (RCT) of add-on Chinese herbal medicine (CHM) external application (FZHFZY) for mild-to-moderate psoriasis vulgaris, as well as to understand the experience and acceptability of patients who participate in the pilot RCT.

This informed consent form provides details of the research project. Knowing what is involved will help you decide if you want to participate in the research. Please read this information carefully. If there is anything you would like to know more about, please feel free to ask questions. Before making any decision, you may consult with your medical doctor or others.

**Introduction**

1. **Background**

Current conventional therapies for the management of psoriasis vulgaris are often associated with insufficient long-term symptomatic relief, high cost and unwanted side effects. There has been certain evidence from clinical studies and systematic reviews supporting the use of CHM external application for the treatment of psoriasis vulgaris, however, a lack of standardised clinical trials and unknown herbal constituents in CHM formulas make it difficult to interpret and replicate the results. The CHM formula *Fu zheng he fu zhi yang* (FZHFZY) developed by Prof. Chuanjian Lu, an experienced clinician of Chinese medicine in dermatology, has been used as an external application for over decades to manage psoriasis symptoms. The safety and potential effects of this formula has been proved by pre-clinical studies and clinical observational studies prior to this randomised controlled trial.

This will be a qualitative study embedded in a pilot RCT. It aims to obtain detailed feedback from patients who participate in the pilot RCT on individual experience and acceptability of the trial. Results from the qualitative study will help us to optimise the study design.

The research is a part of Dr. Junyue Wang’s PhD project. The project is supervised by Prof. Charlie Changli Xue, Prof. Chuanjian Lu, Prof. Anthony Lin Zhang and Dr. Claire Shuiqing Zhang.

This research has been initiated by the researcher Prof. Charlie Changli Xue and Prof. Chuanjian Lu.

This research is funded by the Department of Science and Technology of Guangdong Province. The PhD scholarship is provided by the School of Health and Biomedical Sciences, RMIT University.

This trial will be conducted in the dermatology outpatient clinic at the Guangdong Provincial Hospital of Chinese Medicine (GPHCM). It will recruit 24 – 30 eligible participants.

The project has been approved by the Ethics Committee of the GPHCM (No. BF2022-189-01) and registered with the RMIT University Human Ethics Advisory Network (No. 2022-25746-18453).

1. **The eligible criteria of the study.**

You may participate in this qualitative study if you meet the inclusion criteria: 1) completing the pilot RCT, 2) being willing to discuss your experience with the research team, and 3) written informed consent provided.

The researcher will assess your condition and let you know if you are eligible for the trial.

1. **If I agree to participate, what will I be required to do?**
   1. You will participate in the qualitative study when you have completed the pilot RCT, provided answers to the acceptability question, and then signed this informed consent form.
   2. If you are involved in the study, you will be required to do the following:

We will make an appointment with you for the interview date. It will be conducted within 4 weeks after completing the pilot RCT. It will be set in a quiet clinic room in GPHCM. The interview will take you one to one and half hours. During the interview period, you will be encouraged to speak freely about your experience and acceptability of the pilot RCT.

Interviews will be audio-recorded. We will make field notes relating to non-language details during the interview, such as gestures. The interview will be conducted in Mandarin.

3.3 What other items you will work on with the researcher?

You will visit the hospital on the date as the appointment. Your real experience and feeling are very important.

1. **What are the potential benefits of taking part in this qualitative study?**

You will learn more about the disease after participating in the study. It will help you understand whether the treatment is appropriate for you after discussion with the researcher. You may find this therapy is convenient and beneficial for your psoriasis and then keep using it in the future. On the other hand, your participation in this trial will make contribution to the development of a promising and effective therapy for psoriasis to benefit other patients.

1. **What are the possible risks of taking part in this qualitative study?**

This study is a face-to-face interview, so there will not be any risk associated with interventions. Some of the questions may cause embarrassment or make you feel uncomfortable. You have the right to decline to answer any question or to withdraw from the study at any time. The information you provide will be confidential and will not be accessible to anyone outside of the research team. It may take you some time when you visit the hospital.

1. **Is there any reimbursement?**

You will be reimbursed for A$60 to cover the cost of travelling to the hospital.

1. **What will happen to the information I provide?**

The audio recordings of interviews will be transferred into a password-protected computer for storage. Labels of the recording file will be coded without identifiable information. Transcriptions of recordings will be completed by researchers using documents that will be stored on a password-protected computer. Transcriptions will be de-identified using the same code numbers as recordings. The demographic information of participants will be retained on a password-protected computer by researchers. The password for the computer will be known only by the researchers in this study. Field notes and related interview files will be kept in a locked filing cabinet at the GPHCM. All research data will be stored for a minimum of seven years at RMIT and five years at GPHCM from the publication date. Only members of the research group and the Ethics committee of the GPCHM will have access to these data. Your personal identity will not be disclosed in any public report. We will protect your privacy unless required by law.

The results of the research will be included in the PhD thesis of Dr. Junyue Wang. Once approved, it will be submitted to the RMIT University Research Repository. The finding from the trial will be published in international peer-reviewed journals and will be presented at national conferences. You will not be identified in any publications from the study. Published research data is displayed as group data rather than data related to specific individuals. If you are interested in the results, we can provide you with published journal articles.

1. **Who should I contact if I have any questions?**

Any questions relating to the study can be directed to Dr. Junyue Wang at XXX.

Any complaints relating to the involvement of the study, please contact the Ethics committee of the GPHCM (Tel. XXX).

If there is any factor that influences your willingness to take part in the study, the researcher will contact you in time.

1. **What are my rights as a participant?**

You have the right to withdraw from the study at any time for any reason. You have the right to request that any recording cease and reject to answer any questions. You have the right to have any unprocessed data withdrawn and destroyed. All these will not impact your relationship with the researcher and the quality of medical care provided by the hospital.

1. **What should I do now?**

Please read this information carefully. If there is anything you would like to know more about, please feel free to ask questions.

Before making any decision, you may consult with your medical doctor or others.

Thank you for reading the above information. If you decide to participate in the trial, please contact Dr. Junyue Wang who will make all the study arrangements for you.

You will be given a copy of this informed consent form to keep.

**Signature page for informed consent**

**Project title:** Experience and acceptability of add-on Chinese herbal medicine (CHM) external application (FZHFZY) for mild-to-moderate psoriasis vulgaris: a qualitative study

**Sponsor:** Department of Science and Technology of Guangdong Province

**No. Ethics Approval:** BF 2022-189-01

**Consent**

I have read the above information and have had an opportunity to ask questions and discuss with the researcher. All my questions have been thoroughly answered.

I understand the potential benefits and risks of the project. I freely agree to participate in the study and ensure I have enough time to think about it. I understand that:

- I can contact the researcher for more information at any time.
- I am free to withdraw from the project at any time. I will not be subject to discrimination or revenge if I do not participate in or withdraw from the study, and the quality of medical care provided by the hospital will not be affected.

I agree that the staff from the Therapeutic Goods Administration and the Ethics committee will have access to my research data.

I understand that I will be given a signed copy of this informed consent form to keep.

Finally, I agree with participating in the study.

Signature of participant: Date: Tel.:

I acknowledge that I have provided details of the project, including the potential benefits and risks. I will give a signed copy of this informed consent form to the participant.

Signature of researcher: Date: Tel.:

Telephone number of the Ethics committee of the GPHCM: XXX.
