# Supplementary material for: Add-on effects of Chinese herbal medicine external application (FZHFZY) to topical urea for mild-to-moderate psoriasis vulgaris: Protocol for a double-blinded randomized controlled pilot trial embedded with a qualitative study
Source: PLoS One. 2024 Mar 21;19(3):e0297834. doi: 10.1371/journal.pone.0297834 (PMC10956750; doi:10.1371/journal.pone.0297834)
Supplement: S11 File — (PDF) [file pone.0297834.s012.pdf]

S11 File. Interview guide and questions

Interview guide	Interview questions
**1. In the pilot RCT, the efficacy evaluation and experience of CHM external application for psoriasis vulgaris**	➢ Why did you participate in the clinical trial?<br>➢ What did you expect from the treatment at the start of the trial?<br>➢ What issue caused by the psoriasis you expect to be resolved through the trial?<br>➢ What is your overall impression about the CHM bath therapy?<br>➢ Anything in particular that you liked or found helpful?<br>➢ Anything you did not like or found less helpful?<br>➢ To what extent did the therapy match your expectations—why (not)/how (not)/in what ways?<br>➢ How well the therapy helped you with the issues that you wanted to be resolved?
**2. In the pilot RCT, the safety evaluation and experience of CHM external application for psoriasis vulgaris**	➢ How to evaluate the overall safety of the trial?<br>➢ In the clinical trial, what safety concerns did you have?
**3. Acceptability of the pilot RCT**	➢ Are you satisfied with the effects of the treatment regimen? If yes, why? If not, why?<br>➢ Will you choose the CHM bath therapy again in the future? If yes, why? If not, why?<br>➢ Will you recommend this therapy to others? If yes, why? If not, why?
**4. Clinical uncertainties associated with a large-scale trial**	➢ What is your experience of administration instructions and precautions of FZHFZY granules?<br>➢ Experience of bath temperature/ CHM bath duration/ frequency/ treatment duration/ visits
