# Supplementary material for: Add-on effects of Chinese herbal medicine external application (FZHFZY) to topical urea for mild-to-moderate psoriasis vulgaris: Protocol for a double-blinded randomized controlled pilot trial embedded with a qualitative study
Source: PLoS One. 2024 Mar 21;19(3):e0297834. doi: 10.1371/journal.pone.0297834 (PMC10956750; doi:10.1371/journal.pone.0297834)
Supplement: S12 File — (PDF) [file pone.0297834.s013.pdf]

RMIT Classification: Trusted

S12 File. Study protocol submitted to the Ethics committee

Project title: Add-on Chinese herbal medicine external application (FZHFZY) to topical urea for mild-to-moderate psoriasis vulgaris: a pilot randomised controlled trial embedded with a qualitative interview

Study protocol

Research organisations:

The Guangdong Provincial Hospital of Chinese Medicine (GPHCM) & RMIT University

Principle investigator: Chuanjian Lu & Charlie Changli Xue

Version: 001

Date: 20220708

Investigators:

Prof. Chuanjian Lu, PhD, GPHCM

Prof. Charlie Changli Xue, PhD, RMIT University

Prof. Anthony Lin Zhang, PhD, RMIT University

Dr. Claire Shuiqing Zhang, PhD, RMIT University

Dr. Jingjie Yu, PhD, GPHCM

Dr. Junyue Wang, PhD candidate, RMIT University & GPHCM

Dr. Hao Deng, MD, GPHCM

Dr. Yuhong Yan, PhD, GPHCM

Dr. Danni Yao, PhD, GPHCM

Ms. Shuyan Ye, MD, GPHCM

RMIT Classification: Trusted

1. Trial synopsis

<table>
<tr><td>Title</td><td>Add-on Chinese herbal medicine external application (FZHFZY) to topical urea for mild-to-moderate psoriasis vulgaris: a pilot randomised controlled trial embedded with a qualitative interview</td></tr>
<tr><td>Aims</td><td>To assess the feasibility of the trial protocol qualitatively and quantitatively, and preliminary estimate the add-on efficacy and safety of CHM formula FZHFZY external application to topical urea cream for patients with mild-to-moderate psoriasis vulgaris</td></tr>
<tr><td>Trial design</td><td>A two-arm, parallel, randomised, placebo-controlled, double-blind, pilot trial embedded with a qualitative interview</td></tr>
<tr><td colspan="2">A pilot Randomised Controlled Trial</td></tr>
<tr><td>Participants</td><td>Inclusion criteria

Clinically diagnosed with psoriasis vulgaris
PASI $<$ 10 or BSA $<$ 10%
Age between 18 and 65 years
Written informed consent is provided

Exclusion criteria

Currently are pregnant and/or lactating
Currently have uncontrolled or severe diseases, such as cardiovascular, respiratory, digestive, urinary, haematological or psychiatric diseases; have any known malignancy or a history of malignancy
Are allergic to the medications used in this study
Currently are participating in or have participated in other clinical trial(s) in the previous month
Not complete wash-out requirements of other psoriasis therapies as listed below:

<table>
<tr><th>Therapies</th><th>Washout requirements</th></tr>
<tr><td>Topical agents: Glucocorticoids, Calcineurin inhibitors (e.g. Tacrolimus, Pimecrolimus), Vitamin D analogues (e.g. Calcipotriol, Tacalcitol), Retinoids (e.g. tazarotene), combination treatments (eg. Compound Clobetasol Propionate, Carpotriol betamethasone), Keratin promoter (e.g. 2% – 5% coal tar, 3% salicylic acid, 0.1% – 0.5% anthralin, 5% ichthammol), Keratolytic (e.g. 5% – 10% salicylic acid, 0.1% Retinoids), anthralin, and so on</td><td>Two weeks</td></tr>
<tr><td>Antimicrobials; Systemic nonbiologic therapies: methotrexate, cyclosporine, acitretin, azathioprine, leflunomide, Mycophenolate Mofetil and so on;</td><td>Four weeks</td></tr>
</table>
</td></tr>
</table>

RMIT Classification: Trusted

	Phototherapies: NB-UVB, PUVA, 308 Excimer laser and so on<br>Biologics and their half-lives approved by China now: Etanercept 3.5 days, Infliximab 10 days, Adalimumab 14 days, Ustekinumab 21 days, Guselkumab 18 days, Secukinumab 27 days, Ixekizumab 13 days	Five times of the half-life period of biologics
Intervention group	CHM granules plus 10% urea cream	
Control group	Placebo granules plus 10% urea cream	
Outcomes	Trial feasibility<br>• The proportion of involvement by the number of screened, eligible and consenting participants, as well as the time requirement to recruit eligible participants will be used to assess the feasibility of the study setting, eligible criteria and recruitment strategies<br>• The compliance rate of using trial medications and the acceptability question will be used to quantitatively evaluate the feasibility and acceptability of the trial<br>• Blinding credibility<br>• Determine the primary outcome and provide data for sample size calculation in the full-scale trial<br>Efficacy outcomes<br>• Primary outcome<br>✓ The change in the PASI from baseline to week 8<br>• Secondary efficacy outcomes<br>✓ The PGA response, defined as a PGA score of 0 (clear) or 1 (almost clear) on the 7-point static PGA at week 8<br>✓ The percentage of patients who achieve PASI-75 at week 8<br>✓ The percentage of patients who achieve PASI-50 at week 8<br>✓ The change in the percentage of total BSA affected by psoriasis from baseline to week 8<br>✓ The change in the itch VAS from baseline to week 8<br>✓ The change in the DLQI from baseline to week 8<br>✓ The change in the Skindex-16 from baseline to week 8 and week 20<br>✓ The relapse rate at week 20, defined as a loss of 50% of PASI improvement from baseline in patients who achieve PASI-50 at week 8<br>Safety outcomes<br>✓ The incidence and frequency of adverse events (AEs) and serious AEs during the trial period	

RMIT Classification: Trusted

	✓ Chemical examinations (complete blood count, urinalysis, hepatic and renal functions), vital signs (heart rate, body temperature, respiratory rate and blood pressure), and physical examinations at week 0, week 8 and week 20
Sample size	60 participants
Trial duration	Visit 1: The screening date<br>Visit 2: Baseline (Week 0)<br>An 8-week treatment period: Visit 3 – Visit 6 (Week 2, 4, 6, 8)<br>A 12-week follow-up period: Visit 7 – Visit 9 (Week 12, 16, 20)
Qualitative study	
Participants	Eligible criteria<br>• Completing the pilot RCT<br>• Willing to participate in qualitative interviews<br>• Written informed consent is provided
Outcomes	Obtain detailed qualitative feedback on individual therapeutic evaluation and acceptability of the intervention, as well as their experience with the whole trial
Sample size	24 – 30 eligible participants
Trial duration	Within 4 weeks after the end of the pilot RCT, visit 1

2. Background

Psoriasis is a chronic, recurrent inflammatory condition which is caused by immune stimulation of epidermal keratinocytes [1]. It has been reported that a global cumulative prevalence of psoriasis was between 0.09% and 11.4% in 2016 by WHO [2]. The life-long psoriasis symptoms and the associated comorbidities can significantly impair patients' quality of life (QoL) and cause substantial economic burden [2, 3]. Psoriasis vulgaris is the most common form of the disease accounting for more than 80% of total psoriasis cases [4], which is characterised by raised, well-demarcated, erythematous oval plaques with adherent silvery scales. In addition, psoriasis vulgaris ranked as the tenth most prevalent skin disease (0.9%) and the second largest contributor to all combined skin disability-adjusted life years (0.2%) as reported by the Global Burden of Disease Study in 2017 [5]. Most of psoriasis vulgaris patients are mild-to-moderate severity [6].

For the management of mild-to-moderate psoriasis patients, both topical corticosteroids and vitamin D analogues are recommended with Grade A strength [7]. However, there are certain concerns of adverse effects associated with these medications. For instance, the most common adverse effects of corticosteroids include skin atrophy, striae, folliculitis, telangiectasia, purpura, tachyphylaxis and rebound [8]; 35% of patients may experience burning, pruritus, oedema, peeling, dryness, and erythema using topical vitamin D analogue [7]. Narrowband ultraviolet light B (NB-UVB) phototherapy is also recommended for adult moderate psoriasis patients as monotherapy [9-12]. While the potential concern of NB-UVB is skin cancer and cataracts [13]. Therefore, current conventional therapies for managing patients with mild-to-moderate psoriasis vulgaris are often associated with

RMIT Classification: Trusted

insufficient long-term symptomatic relief and unwanted side effects. And there is a scope to test new potentially effective treatments.

Chinese herbal medicine (CHM) for oral or external application has been practised for thousands of years and with a well-developed theoretical framework [14]. There have been certain research evidence supporting the use of CHM to manage psoriasis vulgaris [15-21]. For the CHM external application, several systematic reviews have reported that this treatment combined with conventional therapies showed promising therapeutic effects for psoriasis vulgaris [22-27]. Additionally, evidence from clinical studies suggests that CHM external application could promote the healing of skin lesions and regulate immune responses [28, 29]. In the latest clinical guidelines, it mentioned that CHM external application as a bath therapy appeared to improve response to conventional therapies for psoriasis, however, it is difficult to interpret and replicate the results because the majority clinical trials lacks standardisation and herbal constituents in CHM formulars are unknown [7].

The CHM formula *Fu zheng he fu zhi yang* (FZHFZY) was developed by Prof. Chuanjian Lu, an experienced clinician of Chinese medicine in dermatology, and has been used as an external application for over decades to manage psoriasis symptoms [30, 31]. The formula has been patented by China National Intellectual Property Administration (No. ZL 2018 1 0749464.0) [32]. As observed in clinical practice, the CHM ingredients of FZHFZY exert the clinical benefit of enhancing the overall health, promoting the healing of skin lesions and relieving pruritus. The pre-clinical study has revealed that 19 potential bioactive compounds were discovered and 13 of them were identified in FZHFZY by three cell lines fishing combined with liquid chromatography-mass spectrometry analysis [30]. The FZHFZY formula regulates epidermal differentiation through inhibition of the Akt/mTORC1/S6K1 pathways [33], which offers important insight on how the FZHFZY formula may benefit patients with psoriasis. In addition, the contents of eight active compounds of the CHM formulation were determined by ultra-performance liquid chromatography, which can be helpful for the quality control of FZHFZY [34]. However, the effect and safety of the formula has not been evaluated by a rigorous randomised controlled trial (RCT). Therefore, we designed the study protocol of a double-blinded, placebo-controlled RCT and will conduct a pilot RCT to explore the feasibility of the protocol. Additionally, the experience and acceptability of patients who participate in the trial will be collected with an embedded qualitative interview [35, 36]. Results from the study will optimise a full-scale study design.

3. Aims and objectives

3.1 Aims

The study will assess the feasibility of the trial protocol qualitatively and quantitatively, and preliminary estimate the add-on efficacy and safety of CHM formula FZHFZY external application to topical urea cream for patients with mild-to-moderate psoriasis vulgaris. Results from the pilot study will optimise the study design.

3.2 Objectives

The pilot trial will have objectives to:

- Assess the feasibility of study setting, eligible criteria, intervention, outcome measurements, participant timeline and recruitment strategies

- Explore the blinding credibility and the acceptability of the trial
- Determine the adequate sample size for a definitive full-scale RCT
- Preliminary estimate of the add-on efficacy and safety in the treatment group (FZHFZY+ urea cream) compared to that in the control group (Placebo+ urea cream).
- Obtain detailed qualitative feedback on the experience and acceptability of patients who complete the pilot RCT.

4. Trial design

The FZHFZY Trial consists of two main parts. For the quantitative and qualitative components, we will collect data concurrently and analyse data sequentially, with quantitative data informing our sampling of qualitative interviews for analysis.

- Part 1: This will be a two-arm, parallel, randomised, placebo-controlled, double-blind, pilot trial. In all, sixty eligible participants will be randomised at a 1:1 ratio to receive eight weeks' treatment of either CHM FZHFZY granules as a bath therapy plus 10% urea cream, or placebo granules plus 10% urea cream, with a 12-week follow-up phase.

- Part 2: This will be an embedded qualitative study with the above-mentioned pilot RCT. The primary source of data collection will be semi-structured interviews via face to face. The data will be analysed through thematic analysis.

5. Part 1: The pilot RCT

The procedure of the pilot RCT is presented in Figure 1 and the schedule of events is provided in Table 1.

RMIT Classification: Trusted

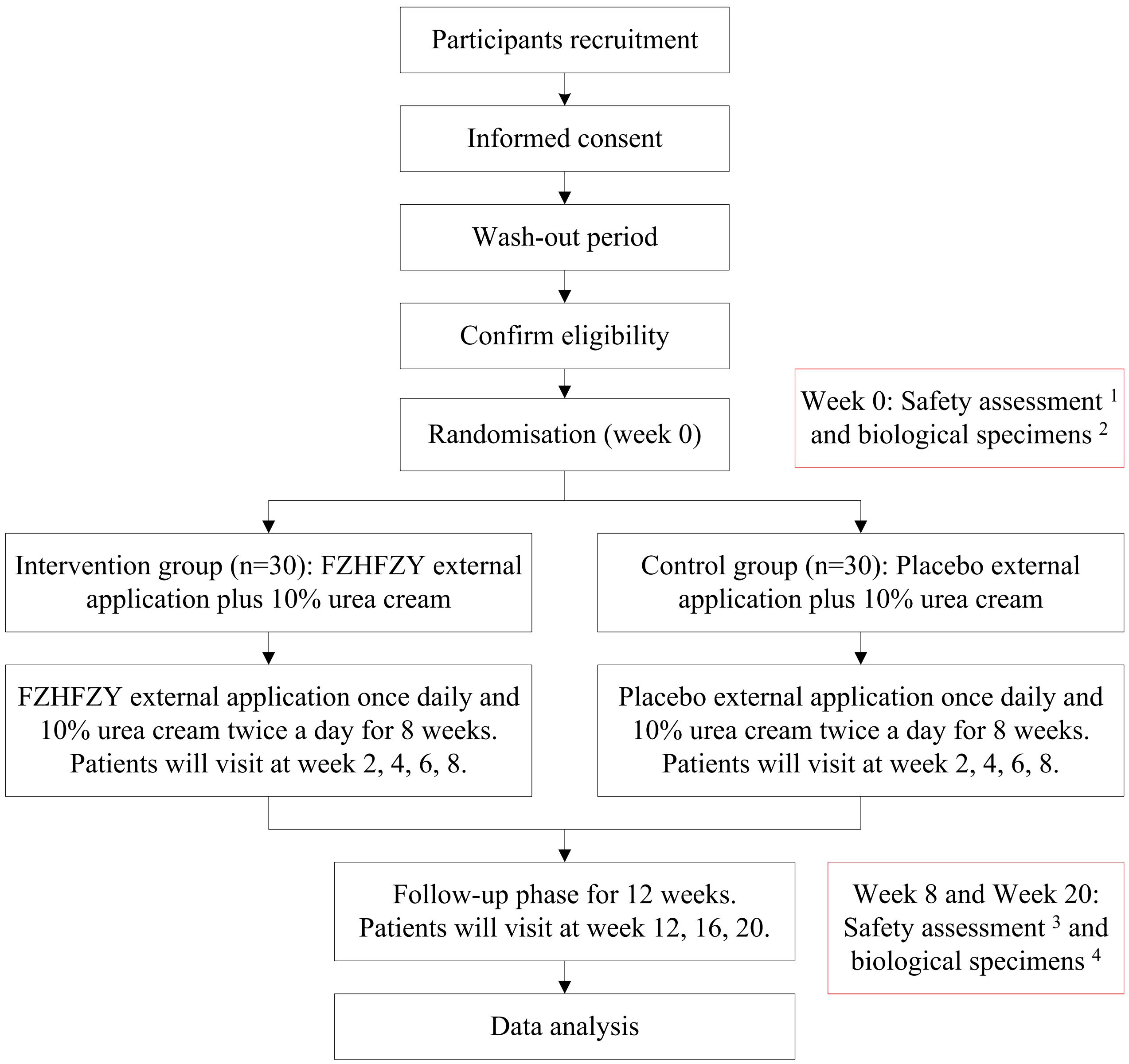

1 Safety assessment: Electrocardiograms, blood (two 4 ml-EDTA tubes and two drying tubes) and urine (one 10 ml-urine tube)
2 biological specimens: blood (three 4 ml-EDTA tubes, three 4 ml-procoagulant tubes, and one 2.5 ml-BD tube), urine (two 10 ml-urine tubes), skin flora (four microtubes), feces (three feces tubes) and oral flora (two microtubes)
3 Safety assessment: blood (two 4 ml-EDTA tubes and two drying tubes) and urine (one 10 ml-urine tube)
4 biological specimens: blood (three 4 ml-EDTA tubes, three 4 ml-procoagulant tubes, and one 2.5 ml-BD tube), urine (two 10 ml-urine tubes), and skin flora (four microtubes)

Figure 1 Flowchart of the trial procedure

RMIT Classification: Trusted

Table 1 Schedule for research activities, interventions and data collection.

	Before Week 0 (wash-out)	Week 0 (Baseline)	Week 2	Week 4	Week 6	Week 8	Week 12	Week 16	Week 20
**Research activities**									
Informed consent	X								
Assess eligibility	X	X							
ECG, blood test, urine test for eligibility assessment		X							
Randomisation		X							
Treatment: CHM/placebo plus urea cream		—	—	—	—	—			
Follow-up							—	—	—
Photos taken for representative skin lesions		X	X	X	X	X	X	X	X
Dispense (D)/Collect (C) trial drugs		D	C/D	C/D	C/D	C			
Dispense (D)/Collect (C) home diary record sheet		D	C/D	C/D	C/D	C/D	C/D	C/D	C
**Data collection: general information**									
Demographics	X	X							
Medical history	X	X							
Concomitant medication	X	X	X	X	X	X	X	X	X
**Data collection: efficacy assessment**									
PASI	X	X	X	X	X	X	X	X	X
BSA	X	X	X	X	X	X	X	X	X
7-point sPGA		X	X	X	X	X	X	X	X

RMIT Classification: Trusted

Itch VAS		X	X	X	X	X	X	X	X
DLQI		X				X			X
Skindex-16		X				X			X
**Data collection: safety assessment**									
Vital signs		X				X			X
Physical examination		X				X			X
Blood test, Urine test						X			X
Reporting AEs		◆—	—	—	—	—	—	—	—◆
**Data collection: feasibility assessment**									
Trial medication usage check			X	X	X	X			
Blinding credibility				X		X			X
Acceptability						X			X
**Data collection: biological specimens**									
Blood, urine, Skin microbiota		X				X			X
Stool and Oral cavity microbiota		X							

Note: AE, adverse event(s); BSA, Body Surface Area; DLQI, Dermatology Life Quality Index; ECG, electrocardiograms; PASI, Psoriasis Area and Severity Index; sPGA, static Physician's Global Assessment; VAS, visual analogue scale.

RMIT Classification: Trusted

5.1 Setting and participants

This RCT will be conducted at the Guangdong Provincial Hospital of Chinese Medicine (GPHCM), Guangzhou, China. The GPHCM is one of the largest Chines medicine hospital with the largest number of annual outpatients and the most powerful hospital in China. There are over 2,000 patients with psoriasis vulgaris visiting the dermatology outpatient clinic at the GPHCM annually. Patients with mild-to-moderate psoriasis vulgaris will be recruited through the poster advertisement and face-to-face consultation. Potential participants may also be recommended to contact the researcher by their physicians (general practitioner, dermatologist or immunologist). Eligible participants will be included in the study after providing written informed consent. The selection criteria are as follows.

5.1.1 Inclusion criteria

- Clinically diagnosed as psoriasis vulgaris [37]
- PASI $<$ 10 or BSA $<$ 10% [7, 38]
- Age between 18 and 65 years
- Written informed consent is provided.

5.1.2 Exclusion criteria

- Currently are pregnant and lactating patients
- Currently have uncontrolled or severe diseases, such as cardiovascular, respiratory, digestive, urinary, haematological or psychiatric diseases; have any known malignancy or a history of malignancy
- Are allergic to the medications used in this study
- Currently are participating in or have participated in other clinical trial(s) in the previous month
- Not complete a wash-out period of psoriasis therapies (see Table 2 for detailed washout requirements of each therapy).

Table 2 Psoriasis therapies and washout requirements.

Therapies	Washout requirements
Topical agents: Glucocorticoids, Calcineurin inhibitors (e.g. Tacrolimus, Pimecrolimus), Vitamin D analogues (e.g. Calcipotriol, Tacalcitol), Retinoids (e.g. tazarotene), combination treatments (eg. Compound Clobetasol Propionate, Carpotriol betamethasone), Keratin promoter (e.g. 2% – 5% coal tar, 3% salicylic acid, 0.1% – 0.5% anthralin, 5% ichthammol), Keratolytic (e.g. 5% – 10% salicylic acid, 0.1% Retinoids), anthralin, and so on [7]	Two weeks
Antimicrobials; Systemic nonbiologic therapies: methotrexate, cyclosporine, acitretin, azathioprine, leflunomide, Mycophenolate Mofetil and so on; Phototherapies: NB-UVB, PUVA, 308	Four weeks

Excimer laser and so on [13, 39, 40]	
Biologics and their half-lives approved by China now [41]: Etanercept 3.5 days, Infliximab 10 days, Adalimumab 14 days, Ustekinumab 21 days, Guselkumab 18 days, Secukinumab 27 days, Ixekizumab 13 days	Five times half-life [42]

5.2 Randomisation and Blinding

A total of 60 eligible participants will be randomly assigned at a 1:1 ratio to one of the two groups at the baseline visit. The equal randomisation will be conducted by the staff at the Key Unit of Methodology in Clinical Research (KUMCR) of the GPHCM who is independent of the trial investigators. Only the person has access to the results of randomised allocation. The random allocation sequence will be generated through the blocked randomisation method using the SAS software (version 9.2, SAS Institute, Inc., Cary, NC, USA). Then the randomisation code corresponding to the trial medications will be released by the Interactive Web Response System for Chinese Medicine Trials which is a verified online randomisation facility established by the KUMCR (http://59.42.21.236:8088/randomisation/portal/portalLogin). Therefore, the allocation concealment will be ensured.

The participants, trial investigators, outcome assessors, statisticians, and the pharmacist who dispenses medications will be blinded to the allocation. The randomisation code can be broken if the appropriate management of the patient in medical emergencies necessitates knowledge of the treatment randomisation. It should be applied by the trial investigator through the online randomisation system and then the principal investigator will be notified to approve of breaking the code. Therefore, the type of trial medications (CHM or placebo) will be identified. In addition, the reason, date, and result of breaking the randomisation code will be recorded in the patient's case report form. Any condition resulting in an unblinding event will be reported to the ethics committee of the GPHCM. Breaking the randomisation code under medical emergencies should not spread to ensure the clinical trial continues. If a patient's treatment is unblinded by the investigator, the patient will be withdrawn from the study.

5.3 Intervention and control

5.3.1 FZHFZY formula

FZHFZY includes seven herbs: *Cynanchum paniculatum* (*Bge.*) *Kitag*. (*xu chang qing*), *Dictamnus dasycarpus Turcz*. (*bai xian pi*), *Cnidium monnieri* (*L.*) *Cuss*. (*she chuang zi*), *Smilax glabra Roxb*. (*tu fu ling*), *Rehmannia glutinosa Libosch*. (*shu di huang*), *Angelica sinensis* (*Oliv.*) *Diels*. (*dang gui*) and *Punica granatum L*. (*shi liu pi*) [30, 31], the dosage ratio of seven herbs is 3:3:2:3:3:2:3 respectively. FZHFZY will be produced into granules by Tianjiang Pharmaceutical Co., Ltd. (Jiangyin, Jiangsu Province, China), a manufacturer holding a Chinese Good Manufacturing Practice certificate. These herbs will be mixed, boiled, filtered and pressure spray-dried to form granules. Then will be packaged in a single-dose sachet (11.0 cm× 15.5 cm), weighing 100 g each. The administration instructions and precautions of the intervention are listed as follows.

5.3.1.1 Administration instructions

One package of FZHFZY granules will dissolve in warm water at 35°C to 38°C. The amount of

water will be adjusted according to the size of the bathtub and the location of the skin lesion. Participants will be instructed to adjust their bathing position to ensure the psoriasis lesions will be soaked by the liquid. The height of the liquid should not exceed the chest when a patient is in a semi-recumbent or sitting position if a whole-body bath is required. Where a whole-body bath is not needed, patients may reduce the amount of CHM/placebo granule and water according to the doctor's instructions. The bathing duration is between 15 and 20 minutes once a day for eight weeks at home.

5.3.1.2 Administration precautions

- The external application as a whole-body bath or topical bath, as well as the amount of water and dosage will depend on the size of the bathtub and the location of the skin lesion, as well as the acceptability of patients.
- Take a bath after cleansing the skin. The right liquid temperature is between 35°C to 38°C according to individual preference.
- The height of liquid should not exceed the chest when a patient is in a semi-recumbent or sitting position if a whole-body bath is required.
- Pay attention to ventilation when bathing the whole body, it is not suitable for closed spaces.
- Avoid getting cold and drinking alcohol and prevent fall injury before and after bathing.
- Please suspend the bath with cold and fever.
- If patients have observed any allergic reaction, please stop the bath therapy and contact the researcher immediately for further instruction.
- If there is any skin damage caused by injuries, please stop the bath therapy and contact the researcher immediately for further instruction.
- If patients feel unwell during the bath, e.g. skin irritation, chest tightness and shortness of breath, please discontinue it.

5.3.2 Placebo

Placebo consisting of starch with no active ingredients will be made by the same manufacturer to match the FZHFZY granules as closely as possible, in terms of their colour, appearance, and smell. The colour will be made identical by adding artificial pigment whilst the taste will be adjusted by adding a medicine intermediate. The administration instructions and precautions for the FZHFZY formula and placebo will be identical.

5.3.3 Co-intervention

Patients in each group will be required to apply 10% urea cream topically on their psoriasis lesions twice a day following the fingertip unit (FTU) method [43]. One FTU is measured as the amount (about 0.5 grams) of medication that covers from the tip of an adult finger to the first crease and it is sufficient to cover both sides of an adult. Participants will be instructed to apply the cream immediately after CHM bath therapy when the skin is still moist. The dosage can be adjusted from its initial dose at the participants' discretion and as their symptoms change. Participants will stop using the cream for 12 hours before collecting Skin microbiota.

5.3.4 Rescue therapy

Cetirizine hydrochloride tablets will be provided as an additional management for unbearable itch caused by psoriasis.

5.3.5 Trial duration

The trial period is 20 weeks, including an 8-week treatment phase and a 12-week follow-up phase.

5.4 Data collection

Eligible participants will be asked to attend an in-person assessment appointment at the dermatology outpatient clinic of GPHCM at eight points: week 0, week 2, week 4, week 6, week 8, week 12, week 16 and week 20. The detailed events and schedule can refer to Table 1.

5.4.1 Clinical data collection

Data will be collected and recorded in the case report forms (CRF). Participants will be required to record a home diary.

5.4.1.1 Baseline data

- Demographics: Date of birth, age, sex, ethnicity, nationality, marital status, education level, height, abdominal circumference, weight, occupation, long-term residence
- Vital signs: Heart rate, body temperature, respiratory rate and blood pressure
- History of allergy: History of allergy to drugs, food or contact
- Diseases and treatment history: Psoriasis incidence and family history, past treatment history and other diseases and treatment history
- Chemical examinations: Complete blood count, urinalysis, hepatic and renal functions, and Electrocardiograms
- PASI, BSA, 7-point sPGA, itch VAS, DLQI and Skindex-16
- Physical examinations

5.4.1.2 Outcome measures and visits

- PASI, BSA, sPGA and itch VAS: Participants will be evaluated these measures at week 0, 2, 4, 6, 8, 12, 16 and 20
- DLQI and Skindex-16: Evaluate these measures at week 0, 8, and 20
- 11-point ordinal scale: Evaluate it at week 8 and 20
- Blinding credibility: Evaluate it at week 8 and 20
- Chemical examinations: Evaluate electrocardiograms at the baseline, and collect blood (two 4 ml-EDTA tubes and two drying tubes) and urine (one 10 ml-urine tube) at week 0, 8 and 20
- Photos taken for representative skin lesions: Conduct it at each visit
- Concomitant medication, and AEs: Record it during the whole trial phase
- Dispense and collect trial drugs: Dispense trial drugs at week 0, 2, 4, and 6; collect trial drugs

RMIT Classification: Trusted

at week 2, 4, 6 and 8

- Dispense and collect home diary record sheet: Dispense it at week 0, 2, 4, 6, 8, 12, and 16; collect it at week 2, 4, 6, 8, 12, 16 and 20

5.4.2 Data collection of biological specimens

After obtaining informed consent from patients, we will collect the biological specimens including blood, urine and stool, skin microbiota and oral cavity microbiota during the trial. All collected specimens will be de-identified to protect patients' privacy unless required by law. These specimens will be stored in the GPHCM for 10 years after the end of the study and will be disposed after the preservation period. Patients have the right to request to dispose the unused biological specimens during the preservation period. These specimens may be used to explore the mechanism of psoriasis in the future and tested in different laboratories as needed. If we want to use these specimens, we will re-submit the ethics application to the Ethics Committee of GPHCM. These specimens can be used after getting ethics approval.

5.4.2.1 Time to collect biological specimens

- Biological specimens: collect blood (two 5 ml-EDTA tubes and two 5 ml-procoagulant tubes), urine (one 10 ml-urine tubes) and skin microbiota (four microtubes) at week 0, 8 and 20; collect blood (one 2.5 ml-BD tube), stool (two stool tubes) and oral cavity microbiota (two microtubes) at week 0.

Table 3 Collecting and storing biological specimens

Biological specimens	Type	Tubes	Time	T/℃	Storage	Objectives
Blood	Serum	10	Week 0, 8 and 20	-80	Refrigerator	Proteomics and metabolomics
	PBMC	5		From -160 to -196	Liquid nitrogen container	Single-cell flow mass spectrometry
Urine	Urine supernatant	10		-80	Refrigerator	Metabolomics
Skin microbiota	Skin microbiota	4		From -160 to -196	Liquid nitrogen container	Skin microflora
Blood	RNA of whole blood	1	Week 0	-80	Refrigerator	Immune omics
Stool	Stool	2		-80	Refrigerator	Gut microbiota
Oral cavity microbiota	Oral cavity microbiota	2		From -160 to -196	Liquid nitrogen container	Oral microflora

5.5 Termination and withdrawal

If participants are diagnosed with other types of psoriasis (erythrodermic psoriasis, psoriatic arthritis, or pustular psoriasis) or suffer from serious adverse events during the trial, the study will be terminated. In addition, participants have the right to withdraw from the study at any time for any reason. Trial investigators can receive feedback on trial medications from these participants and ask them to complete the relevant examinations, which will help the researcher collect data and benefit the care of participants' health.

5.6 Outcome measures

5.6.1 Trial feasibility

- The proportion of involvement by the number of screened, eligible and consenting participants, as well as the time requirement to recruit eligible participants will be used to assess the feasibility of the study setting, eligible criteria and recruitment strategies
- The compliance rate of using trial medications and the acceptability question will be used to quantitatively evaluate the feasibility and acceptability of the trial [44]
- Blinding credibility at week 4, week 8 and week 20
- Determine the primary outcome and provide data for sample size calculation in the full-scale trial

5.6.2 Efficacy outcomes

5.6.2.1 Primary outcome

- Change in the PASI total score from baseline to week 8 [45]

5.6.2.2 Secondary efficacy outcomes

- The PGA response, defined as a PGA score of 0 (clear) or 1 (almost clear) on the 7-point static PGA at week 8 [46]
- The percentage of patients who achieve at least 75% improvement in PASI score from baseline (PASI-75) to week 8 [47]
- The percentage of patients who achieve at least 50% improvement in PASI score from baseline (pasi-50) to week 8 [47]
- The change in the percent of total BSA affected by psoriasis from baseline to week 8 [45]
- The change in the total itch VAS from baseline to week 8 [48]
- The change in the DLQI total score from baseline to week 8 [49]
- The change in the Skindex-16 total score from baseline to week 8 [50]
- The relapse rate at week 20, defined as a loss of 50% of PASI improvement from baseline in patients who achieve PASI-50 at week 8 [51].

5.6.3 Safety outcomes

Safety assessments will consist of incidence and frequency of adverse events (AEs) and serious AEs during the trial period, the evaluation of application site tolerability, clinical laboratory parameters

(complete blood count, urinalysis, hepatic and renal functions), vital signs (heart rate, body temperature, respiratory rate and blood pressure), and physical examinations at week 0, week 8 and week 20. Each patient with an adverse event will be counted only once for each Medical Dictionary for Regulatory Activities (MedDRA), version 22.0, preferred term [52]. The severity of AEs will be based on the Common Terminology Criteria for Adverse Events, version 5.0 [53]. The WHO-UMC system will be used for standardised case causality assessment [54]. Serious AEs should be reported to the GPHCM Ethics administration within 24 hours and be recorded in the serious AEs form.

5.7 Pre-assessment and disposal plan of relevant risks

5.7.1 Adverse reaction of CHM bath therapy

The common adverse reaction of CHM bath therapy is skin irritation (e.g. skin erythema, itching), chest tightness and shortness of breath. These symptoms may disappear if participants stop bathing immediately. If participants suffer from any unexpected condition no matter whether it is related to the trial medications or not, they can contact the researcher immediately for further suggestions.

5.7.2 Other types of psoriasis

If participants in the trial are diagnosed with other types of psoriasis (erythrodermic psoriasis, psoriatic arthritis, or pustular psoriasis) by researchers, the study will be terminated. They will be treated according to psoriasis clinical guidelines.

5.8 Data management

All physicians, assessors and research assistants will attend training workshops before the conduction of the trial. Investigators will be provided with a written protocol and required to follow the standard operating procedures. The quality controllers from the Guangdong International Clinical Research Centre of Chinese Medicine (Guangzhou, China) will undertake the monitoring tasks of the trial. All data will be entered into a pre-designed, password-protected dataset by personnel blinded to group allocation. Data entry will be performed continuously throughout the study using the double-check method, with any correction or changes of written data in participants' case report forms documented and dated. Any information obtained in connection with this research project will be de-identified using unique study codes. A reference table linking the study code to the participant's details will be stored separately in a secured filing cabinet. Only study investigators will have access to the information. Research data will be kept secure at the GPHCM and the RMIT University for a minimum of 15 years after publication before being destroyed [55].

5.9 Statistical methods

5.9.1 Sample size calculation

Since this is a pilot study, a sample size calculation is not performed. The pilot study aims for 60 participants because a pilot study with at least n = 50 is advisable in many circumstances at a high level of confidence [56], considering a 15% loss to follow-up.

5.9.2 Quantitative data analysis

Statistical analysis will be performed in a blinded manner by qualified statisticians using PASW Statistics 25.0 (IBM SPSS Inc., Armonk, New York, USA). All statistical tests are two-sided with a *P* value of <0.05 being considered statistically significant.

Efficacy analyses will be based on the intention-to-treat (ITT) population and safety analyses will be based on the safety analyses set population, both of them defined as all randomised patients who received at least one dose of study treatment and the data of at least one visit. Missing data will be replaced by a last observation carried forward approach for ITT analysis. For the outcome variables, continuous data (e.g. mean change in PASI score, BSA, itch VAS, and DLQI) will be summarised as mean and standard deviation (data with normal distribution) or mean and interquartile range (data without normal distribution), and categorical data (e.g. PASI-50, PASI-75, and relapse rate) will be summarised as counts and percentages. The primary analyses for primary and secondary outcome variables will be based on the ITT population approach, using data collected at baseline, week 8 and week 20.

When outcome variables are continuous data, using an independent t-test (data with normal distribution) or Mann-Whitney U test (data without normal distribution) compares the difference between two groups. When outcomes variables are categorical data, using the Chi-squared test (total samples $\geqslant$ 40 and expectation $\geqslant$ 5) or Fisher's exact test (total samples $<$40 or expectation $<$1) compares the difference between the two groups. The efficacy outcomes will be assessed by a generalised linear mixed model for repeated measures.

6. Part 2: The nested qualitative study

6.1 Study setting

The study will be set in a quiet clinic room of the GPHCM. Participants who have completed the Pilot RCT will participate in the qualitative study. Eligible participants will be provided additional informed consent.

6.2 Participant selection

An interview invitation will be provided when participants complete the pilot RCT of the FZHFZY Trial and fill out the acceptability survey. Acceptability will be measured on an ordinal scale from 0 to 10, where 0 equals very dissatisfied with the pilot RCT and 10 equals very satisfied with the pilot RCT. It is satisfactory for patients if the score is over five points. When the score is less than five points, it means the patient is unsatisfactory with the pilot RCT [44]. We will seek to increase the diversity by purposively recruiting a maximum variation sample of participants according to age, gender, history and severity of psoriasis vulgaris, address, education level, working status, treatment adherence, response to treatment, and the preliminary results of the pilot study. Within this context, we seek to achieve diversity in the sample to ensure that a broad range of views, characteristics and experiences of participation will be obtained [57, 58].

6.3 Data collection

Data collection will be performed through qualitative, semi-structured interviews with an interview guide of open-ended questions. It will be conducted within 4 weeks after completing the pilot RCT through teleconference or face-to-face in a quiet clinic room at GPHCM, depending on participants' preference. During the interviews, participants will be encouraged to speak freely about their efficacy and safety evaluation and experience with CHM external application for psoriasis vulgaris. Subsequently, they will primarily be asked about their experiences with and perspectives on the entire pilot FZHFZY RCT, and the acceptability of the pilot RCT. We will use the interview guide

and questions flexibly to respond to the participant's pace and experience. It means each participant will be encouraged to tell the story in the best suitable way. The duration of the interviews will range from one to one and a half hours. We will make field notes during each interview and summarise these at interview completion. With participants' permission, interviews will be audio-recorded and transcribed verbatim.

An interview guide (Table 4) will be developed based on a review of the relevant academic literature addressing similar questions and psoriasis patients' experience of using Chinese herbal medicine [59-63].

Table 4 Interview guide and interview questions

Interview guide	Interview questions
**1. In the pilot RCT, the efficacy evaluation and experience of CHM external application for psoriasis vulgaris**	➢ Why did you participate in the clinical trial?<br>➢ What did you expect from the treatment at the start of the trial?<br>➢ What issue caused by the psoriasis you expect to be resolved through the trial?<br>➢ What is your overall impression about the CHM bath therapy?<br>➢ Anything in particular that you liked or found helpful?<br>➢ Anything you did not like or found less helpful?<br>➢ To what extent did the therapy match your expectations—why (not)/how (not)/in what ways?<br>➢ How well the therapy helped you with the issues that you wanted to be resolved?
**2. In the pilot RCT, the safety evaluation and experience of CHM external application for psoriasis vulgaris**	➢ How to evaluate the overall safety of the trial?<br>➢ In the clinical trial, what safety concerns did you have?
**3. Acceptability of the pilot RCT**	➢ Are you satisfied with the effects of the treatment regimen? If yes, why? If not, why?<br>➢ Will you choose the CHM bath therapy again in the future? If yes, why? If not, why?<br>➢ Will you recommend this therapy to others? If yes, why? If not, why?
**4. Clinical uncertainties associated with a large-scale trial**	➢ What is your experience of administration instructions and precautions of FZHFZY granules?

	➢ Experience of bath temperature/ CHM bath duration/ frequency/ treatment duration/ visits

6.4 Sample size

The sample size will be expected to be between 24 and 30. In addition, we will estimate the number of participants by data saturation which is defined as no new information having been obtained from the further interview [64]. Therefore, the final sample size will be determined by data saturation.

6.5 Qualitative data analysis

For analysing qualitative data, thematic analysis based on an inductive approach will be conducted [65, 66]. Thematic analysis is described as a flexible approach to analysis that has theoretical freedom, so it is possible to apply a constructivist paradigm and an inductive approach to arrive at a detailed interpretive account of the data [66]. There are six phases of the analytical approach [67]: 1) Familiarization: read and re-read transcripts and field notes to become familiar with the data; 2) generate codes; 3) construct candidate themes; 4) revise themes; 5) define themes; and (6) produce the report. A series of themes and sub-themes will be generated following this process based on grounded theory. All the interviews will be transcribed in full and verbatim by using the original language (Chinese). Transcripts will be de-identified, coded and uploaded to NVivo 12 software (QSR International Version 12, 2018) for data management and data coding [68]. Transcripts will be coded line by line to label themes. Preliminary data analysis will be conducted to determine if the interview guide needs to be revised after every interview.

6.6 Pilot interview

A pilot interview of two participants (one male, one female) will be conducted separately from the main data collection, using the same inclusion criteria. The pilot interview will allow the researcher to develop an interview technique and test the interview guide for validity.

6.7 Data security

The audio recordings of interviews will be transferred to a password-protected computer for storage. Labels of the recording file will be code numbers without identifiable information. Transcriptions of the recording will be completed by researchers using a document which will be stored on a password-protected computer. Transcriptions will be de-identified by using the same code numbers consistent with the recording stored on the computer. The demographic information of participants will be retained on a password-protected computer by researchers. The password for the computer will be known only among the researchers in this study. Field notes and related interview files will be kept in a locked filing cabinet at the GPHCM and RMIT University. Research data will be stored securely for a minimum of seven years at the RMIT and five years at the GPHCM [55]. Only members of the research group and the Ethics committee of the GPCHM will have access to these data. The personal identity of patients will not be disclosed in any public report. We will protect patients' privacy unless required by law.

7. Ethics and dissemination

Ethics approval will be obtained from the Ethics Committee of the GPHCM. The protocol will be registered with the RMIT University Human Ethics Advisory Network. The informed consent form provides details of the research project, it will be signed by the participant and researcher. The

participant will be given a copy of the document to keep.

The results of the clinical trial will be published in peer-reviewed journals. All subjects will be required to obtain permission to publish the findings of the trial and to ensure anonymity and confidentiality. All data will be processed under the rules of the government and law. All researchers will guarantee the anonymity of patients and will not disclose the names of patients unless required by law. Only authorized individuals can obtain the patient's information.

RMIT Classification: Trusted

References

1. Nestle, F.O., D.H. Kaplan, and J. Barker, *Psoriasis.* N Engl J Med, 2009. **361**(5): p. 496-509.
2. World Health Organization, *Global report on psoriasis*. 2016, Geneva: World Health Organization.
3. Elmets, C.A., et al., *Joint AAD-NPF guidelines of care for the management and treatment of psoriasis with awareness and attention to comorbidities.* J Am Acad Dermatol, 2019. **80**(4): p. 1073-1113.
4. Armstrong, A.W. and C. Read, *Pathophysiology, Clinical Presentation, and Treatment of Psoriasis: A Review.* JAMA, 2020. **323**(19): p. 1945-1960.
5. Mehrmal, S., et al., *Identifying the prevalence and disability-adjusted life years of the most common dermatoses worldwide.* J Am Acad Dermatol, 2020. **82**(1): p. 258-259.
6. Yeung, H., et al., *Psoriasis severity and the prevalence of major medical comorbidity: a population-based study.* JAMA Dermatol, 2013. **149**(10): p. 1173-9.
7. Elmets, C.A., et al., *Joint AAD-NPF Guidelines of care for the management and treatment of psoriasis with topical therapy and alternative medicine modalities for psoriasis severity measures.* J Am Acad Dermatol, 2021. **84**(2): p. 432-470.
8. Abraham, A. and G. Roga, *Topical steroid-damaged skin.* Indian J Dermatol, 2014. **59**(5): p. 456-459.
9. Rim, J.H., Y.B. Choe, and J.I. Youn, *Positive effect of using calcipotriol ointment with narrow-band ultraviolet B phototherapy in psoriatic patients.* Photodermatol Photoimmunol Photomed, 2002. **18**(3): p. 131-134.
10. Woo, W.K. and K.E. McKenna, *Combination TL01 ultraviolet B phototherapy and topical calcipotriol for psoriasis: a prospective randomized placebo-controlled clinical trial.* Br J Dermatol, 2003. **149**(1): p. 146-150.
11. El-Saie, L.T., et al., *Effect of narrowband ultraviolet B phototherapy on serum folic acid levels in patients with psoriasis.* Lasers Med Sci, 2011. **26**(4): p. 481-485.
12. Vun, Y.Y., et al., *Generalized pustular psoriasis of pregnancy treated with narrowband UVB and topical steroids.* J Am Acad Dermatol, 2006. **54**(2 Suppl): p. S28-30.
13. Elmets, C.A., et al., *Joint American Academy of Dermatology-National Psoriasis Foundation guidelines of care for the management and treatment of psoriasis with phototherapy.* J Am Acad Dermatol, 2019. **81**(3): p. 775-804.
14. Zhang, C.S. and J. Yu, *Psoriasis Vulgaris*. Evidence-based Clinial Chinese Medicine, ed. Charlie Changli Xue and C. Lu. Vol. 2. 2017: World Scientific.
15. Dai, D., et al., *Evidence and potential mechanisms of traditional Chinese medicine for the treatment of psoriasis vulgaris: a systematic review and meta-analysis.* J Dermatolog Treat, 2022. **33**(2): p. 671-681.
16. Luo, Y., et al., *Chinese Herbal Medicine for Psoriasis: Evidence From 11 High-Quality Randomized Controlled Trials.* Front Pharmacol, 2020. **11**: p. 599433.
17. Parker, S., et al., *Oral Chinese herbal medicine versus placebo for psoriasis vulgaris: A systematic review.* J Dermatolog Treat, 2017. **28**(1): p. 21-31.
18. Zhang, C.S., et al., *Is Oral Chinese Herbal Medicine Beneficial for Psoriasis Vulgaris? A Meta-Analysis of Comparisons with Acitretin.* J Altern Complement Med, 2016. **22**(3): p. 174-88.
19. Yang, L., et al., *Efficacy of combining oral Chinese herbal medicine and NB-UVB in treating psoriasis vulgaris: a systematic review and meta-analysis.* Chin Med, 2015. **10**: p. 27.
20. Zhang, C.S., et al., *Oral Chinese herbal medicine combined with pharmacotherapy for psoriasis vulgaris: a systematic review.* Int J Dermatol, 2014. **53**(11): p. 1305-18.
21. May, B.H., et al., *Oral herbal medicines for psoriasis: a review of clinical studies.* Chin J Integr Med, 2012. **18**(3): p. 172-8.

RMIT Classification: Trusted

22. Duan X, Cheng Y, and Chu L, *Efficacy and safety of Chinese herb bath plus narrow-band UVB for psoriasis vulgaris: a systematic review.* Chin J Dermatovenereol, 2013. **27**: p. 192–195.
23. Guan, J., et al., *Effectiveness and safety of traditional Chinese medical bath therapy combined with ultraviolet irradiation in the treatment of psoriasis: A systematic review and meta-analysis of randomized controlled trials.* PLoS One, 2017. **12**(3): p. e0173276.
24. Wu Y, Gao Y, and Zhu X, *Efficacy and safety evaluation of traditional Chinese medicine bath combined with NB-UVB in the treatment of psoriasis vulgaris.* Asia Pac Tradit Med, 2019. **15**: p. 167-172.
25. Xu J, Yang M, and Chen L, *A meta-analysis of the efficacy and quality of life of Chinese herb bath in the treatment of psoriasis vulgaris.* Lishizhen Med Mater Med Res, 2019. **30**: p. 2028-2032.
26. Yu, J.J., et al., *Add-on effect of chinese herbal medicine bath to phototherapy for psoriasis vulgaris: a systematic review.* Evid Based Complement Alternat Med, 2013. **2013**: p. 673078.
27. Lei, H., et al., *A systematic review and meta-analysis on the efficacy and safety of traditional Chinese medicine bath in the treatment of psoriasis vulgaris.* Ann Palliat Med, 2021. **10**(10): p. 10674-10683.
28. Ying Xi, et al., *The Effect of Traditional Chinese Medicinal Bath Combined with Halometasone Cream on Ceramide and Barrier Function of Stratum Corneum in Patients with Psoriasis.* Progress in Morden Biomedicine, 2021. **21**(23): p. 4593-4597.
29. Li, T., et al., *Effect of Traditional Chinese Medicine plus narrow-band medium-wave ultraviolet B radiation on moderate-to-severe psoriasis vulgaris in a case series.* J Tradit Chin Med, 2019. **39**(5): p. 692-699.
30. Chen, L., et al., *Decoding active components in a formulation of multiple herbs for treatment of psoriasis based on three cell lines fishing and liquid chromatography-mass spectrometry analysis.* J Pharm Biomed Anal, 2020. **186**: p. 113331.
31. The Chinese Medicine Board of Australia (CMBA) and The Australian Health Practitioner Regulation Agency (Ahpra). *USER GUIDER: NOMENCLATURE COMPENDIUM OF COMMONLY USED HERBS AND OTHER INGREDIENTS IN CHINESE MEDICINE.* June 2020; Available from: https://www.chinesemedicineboard.gov.au/documents/default.aspx?record=WD15%2F18882&dbid=AP&chksum=EU9evdZxFjnGJZH0UJFEJQ%3D%3D.
32. Chuanjian Lu, et al. *External application of Chinese herbal medicine for psoriasis* 2021; Available from: http://epub.cnipa.gov.cn/Sw/SwDetail.
33. Lu, Y., et al., *Fuzhenghefuzhiyang Formula (FZHFZY) Improves Epidermal Differentiation via Suppression of the Akt/mTORC1/S6K1 Signalling Pathway in Psoriatic Models.* Front Pharmacol, 2021. **12**: p. 650816.
34. Liu Lijuan, et al., *Establishment of UPLC Fingerprint of Hefu Zhiyang Decoction and Content Determination of 8 Components.* China Pharmacy, 2021. **32**(16): p. 1982-1987.
35. Lewin, S., C. Glenton, and A.D. Oxman, *Use of qualitative methods alongside randomised controlled trials of complex healthcare interventions: methodological study.* Bmj, 2009. **339**: p. b3496.
36. Soliman, E.Z., et al., *A Polypill for primary prevention of cardiovascular disease: a feasibility study of the World Health Organization.* Trials, 2011. **12**: p. 3.
37. Scottish Intercollegiate Guidelines Network. *Diagnosis and management of psoriasis and psoriatic arthritis in adults*. 2010; Available from: http://www.sign.ac.uk.
38. Committee on Psoriasis and Chinese Society of Dermatology, *Guideline for the diagnosis and treatment of psoriasis in China (2018 complete edition).* Chin J Dermatol, 2019(10): p. 667-710.
39. Liu, Y., et al., *Skin microbiota analysis-inspired development of novel anti-infectives.* Microbiome, 2020. **8**(1): p. 85.

RMIT Classification: Trusted

40. Menter, A., et al., *Joint American Academy of Dermatology-National Psoriasis Foundation guidelines of care for the management of psoriasis with systemic nonbiologic therapies.* J Am Acad Dermatol, 2020. **82**(6): p. 1445-1486.
41. Chinese Society of Dermatology, China Dermatologist Association, and Dermatology and Venereology Specialized Committee of Chinese Association of Integrative Medicine, *Guidelines for the treatment of psoriasis with biologic agents in China (2021).* Chin J Dermatol, 2021. **54**(12): p. 1033-1047.
42. Warren, R.B., et al., *An intensified dosing schedule of subcutaneous methotrexate in patients with moderate to severe plaque-type psoriasis (METOP): a 52 week, multicentre, randomised, double-blind, placebo-controlled, phase 3 trial.* Lancet, 2017. **389**(10068): p. 528-537.
43. PAAS National. *Topical medication days' sipply*. 2018; Available from: http://www.ncpa.co/pdf/topical-medication-chart.pdf.
44. Kleiss, I., et al., *A Comparison of 4 Single-Question Measures of Patient Satisfaction.* Journal of clinical outcomes management: JCOM, 2020. **27**: p. 41-48.
45. Fredriksson, T. and U. Pettersson, *Severe psoriasis--oral therapy with a new retinoid.* Dermatologica, 1978. **157**(4): p. 238-44.
46. Langley, R.G. and C.N. Ellis, *Evaluating psoriasis with Psoriasis Area and Severity Index, Psoriasis Global Assessment, and Lattice System Physician's Global Assessment.* J Am Acad Dermatol, 2004. **51**(4): p. 563-569.
47. Nast, A., et al., *S3 - Guidelines on the treatment of psoriasis vulgaris (English version). Update.* J Dtsch Dermatol Ges, 2012. **10 Suppl 2**: p. S1-95.
48. Fron Stratton Hill C. *Guidelines for Treatment of Cancer Pain: The Revised Pocket Edition of the Final Report of the Texas Cancer Council's Workgroup on Pain Control in Cancer Patients, 2nd Edition*. 1997; Available from: https://www.texascancer.info/gftocp/appendixa1.html.
49. AY Finlay and GK Khan. *Dermatology Life Quality Index*. 1992; Available from: https://www.cardiff.ac.uk/medicine/resources/quality-of-life-questionnaires/dermatology-life-quality-index.
50. Chren, M.M., *The Skindex instruments to measure the effects of skin disease on quality of life.* Dermatol Clin, 2012. **30**(2): p. 231-6, xiii.
51. Carey, W., et al., *Relapse, rebound, and psoriasis adverse events: an advisory group report.* J Am Acad Dermatol, 2006. **54**(4 Suppl 1): p. S171-181.
52. The International Council for Harmonisation of Technical Requirements for Pharmaceuticals for Human Use (ICH) organisation. *The Medical Dictionary for Regulatory Activities (MedDRA) system organ classes*. 2016; Available from: https://www.meddra.org/About%20MedDRA%20/%20Evolution%20/%2027th-system-organ-class.
53. U.S. DEPARTMENT OF HEALTH AND HUMAN SERVICES. *Common Terminology Criteria for Adverse Events (CTCAE) Version 5.0*. November 27, 2017; Available from: https://ctep.cancer.gov/protocoldevelopment/electronic_applications/docs/ctcae_v5_quick_reference_5x7.pdf.
54. World Health Organization. *The use of the WHO-UMC system for standardised case causality assessment*. 2013; Available from: https://www.who.int/publications/m/item/WHO-causality-assessment.
55. National Health and Medical Research Council, Australian Research Council, and Universities Australia. *Australian code for the responsible conduct of research Australia*. 2018; Available from: https://www.nhmrc.gov.au/about-us/publications/australian-code-responsible-conduct-research-2018.
56. Sim, J. and M. Lewis, *The size of a pilot study for a clinical trial should be calculated in relation to considerations of precision and efficiency.* J Clin Epidemiol, 2012. **65**(3): p. 301-8.
57. Ritchie, J., et al., *Qualitative research practice : a guide for social science students and*

RMIT Classification: Trusted

researchers. Second edition. ed. 2014, Los Angeles: SAGE.
58. Teddlie, C. and F. Yu, *Mixed Methods Sampling: A Typology With Examples.* Journal of mixed methods research, 2007. **1**(1): p. 77-100.
59. Sugg, H.A.-O., J.A.-O. Frost, and D.A.-O. Richards, *Morita Therapy for depression (Morita Trial): an embedded qualitative study of acceptability.* (2044-6055 (Electronic)).
60. Devall, A.A.-O.X., et al., *Mifepristone and misoprostol versus placebo and misoprostol for resolution of miscarriage in women diagnosed with missed miscarriage: the MifeMiso RCT.* (2046-4924 (Electronic)).
61. Chen, X.A.-O., et al., *Effect of an exercise-based cardiac rehabilitation program "Baduanjin Eight-Silken-Movements with self-efficacy building" for heart failure (BESMILE-HF study): study protocol for a randomized controlled trial.* (1745-6215 (Electronic)).
62. Bobbink, P.A.-O.X., P.J. Larkin, and S.A.-O. Probst, *Experiences of Venous Leg Ulcer persons following an individualised nurse-led education: protocol for a qualitative study using a constructivist grounded theory approach.* (2044-6055 (Electronic)).
63. Coyle, M.E., et al., *Patient experiences of using Chinese herbal medicine for psoriasis vulgaris and chronic urticaria: a qualitative study.* J Dermatolog Treat, 2020. **31**(4): p. 352-358.
64. Morse, J.M., *The Significance of Saturation.* Qualitative health research, 1995. **5**(2): p. 147-149.
65. Chapman, A.L., M. Hadfield, and C.J. Chapman, *Qualitative research in healthcare: an introduction to grounded theory using thematic analysis.* The Journal of the Royal College of Physicians of Edinburgh, 2015. **45**(3): p. 201-205.
66. Braun, V. and V. Clarke, *Using thematic analysis in psychology.* Qualitative research in psychology, 2006. **3**(2): p. 77-101.
67. Liamputtong, P., *Handbook of research methods in health social sciences*. 2019, Singapore: Springer.
68. Bazeley, P. and K. Jackson, *Qualitative data analysis with NVivo*. Second Edition. ed. 2013, Los Angeles: SAGE.
